# Dynamic Pseudorotaxane Crystals Containing Metallocene Complexes

**DOI:** 10.1038/s41598-017-14505-7

**Published:** 2017-10-27

**Authors:** Kai-Jen Chen, Pei-Lin Chen, Masaki Horie

**Affiliations:** 1 Department of Chemical Engineering, National Tsing Hua University, 101, Sec. 2, Kuang-Fu Road, Hsinchu, 30013 Taiwan; 2 Instrumentation Center, National Tsing Hua University, 101, Sec. 2, Kuang-Fu Road, Hsinchu, 30013 Taiwan

## Abstract

Molecular machines and switches composed of flexible pseudorotaxanes respond to external stimuli, transducing incident energy into mechanical motions. This study presents thermo- and photoresponsive dynamic pseudorotaxane crystals composed of axle molecules containing ferrocene or ruthenocene groups threaded through dibenzo[24]crown-8 ether rings. The ruthenocene-containing pseudorotaxane exhibits a crystal-to-crystal thermal phase transition at 86 °C, which is much lower than that of the ferrocene-containing pseudorotaxane (128 °C). Single-crystal X-ray crystallography at various temperatures reveals the details of the structural changes, and shows that the bulky ruthenocene provides distortion in the pseudorotaxane structure to facilitate twisting of the axle molecule. A mixed ferrocene and ruthenocene pseudorotaxane crystal is applied to photomechanical conversion under 405 nm laser irradiation at 85 °C and provides a lifting force 6,400-times the weight of the crystal itself upon phase transition.

## Introduction

Molecular machines and switches are of significant interest because they can be specifically designed to transduce chemical, electrical and photochemical energy into controllable mechanical motion^1–10^. In this context, rotaxanes, which comprise a molecular axle threaded through a ring molecule, undergo changes to the interactions between the axle and ring components under the influence of external stimuli, and operate as one-dimensional molecular switches^7–10^. To enhance the action of these molecular switches to the observable macroscopic level, structural control in the solid state is a key issue. For example, molecular muscles have been achieved using highly-oriented rotaxane films on substrates^11^, and a rotaxane-based dry-type molecular muscle has been demonstrated as a photoresponsive actuator^12^. However, despite the rigid order of the solid state being advantageous for the integration and transmission of molecular motion, there are few reports in the literature on the structural control of rotaxanes in the crystal state^13–16^.

Recently, stimuli-responsive dynamic molecular crystals have been gaining considerable research attention. The dynamics of these crystals include bending, twisting, rotation and salient effects such as hopping and flipping^17–26^, allowing their potential application as actuators^23–32^. These dynamics are accompanied by molecular structural changes induced by thermal stimulus^17–19,33,34^ or photochemical reactions such as photoaddition^35–37^, photoisomerisation^22,38,39^, or photocyclisation^40^ in the crystal state. Some of these molecular structures and their stimuli-responsive structural changes have been precisely determined by single-crystal X-ray crystallography^18–21,31,33,36–38^. Such detailed structural characterisation is crucial for investigating the relationships between the molecular-level motion and the observable motion of crystals at the macroscopic scale.

Most of these dynamic crystals comprise a single molecule with reactive functional groups. However, in order to tailor molecular motions and provide multifunctionality, the use of interlocked molecular systems such as pseudorotaxanes is promising because they are composed of different functional molecules, i.e. a ring molecule with a flexible structure and host functionality, and a guest axle molecule that threads into the host. Adjusting and optimising the functionality of these components is a possible strategy for endowing pseudorotaxanes with controllable dynamics in the solid state.

**Figure 1:**
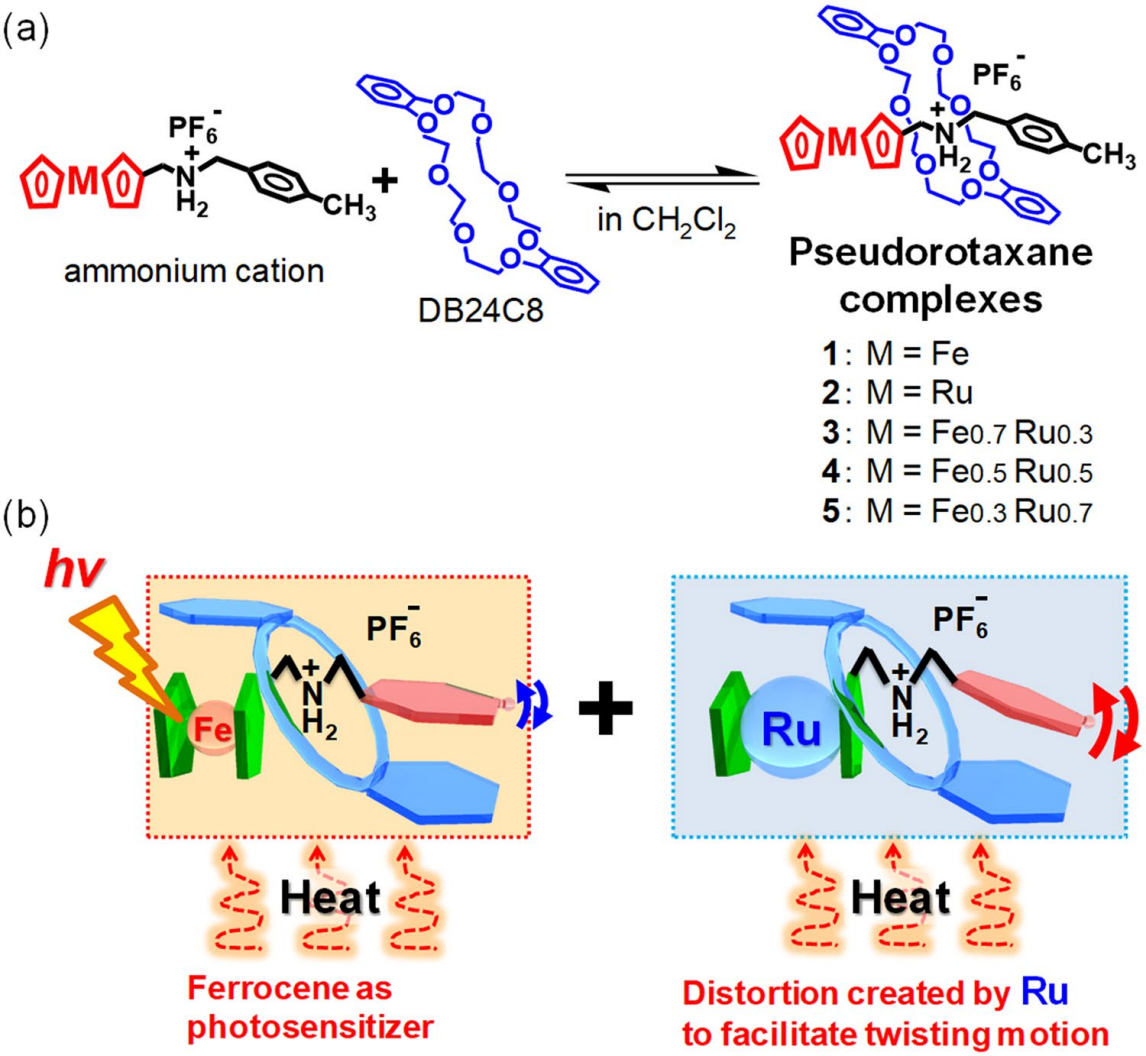
Pseudorotaxanes composed of Fe and Ru complexes. (**a**) Formation of pseudorotaxane molecules with different Fe and Ru ratios in dichloromethane solutions. (**b**) Illustration of stimuli-responsive dynamic pseudorotaxanes in the crystal state.

Recently, we reported the thermal and photoinduced crystal-to-crystal phase transitions of a pseudorotaxane composed of a ferrocenylmethyl(4-methylphenyl)ammonium axle and a dibenzo[24]crown-8 ether (DB24C8) ring (complex **1** in Fig. 1)^41–43^. This unique crystal behaviour was exploited for the remote control of microparticle transport and microswitching in an electric circuit^43^. During photomechanical energy conversion, the ferrocenyl group in the axle molecule of the pseudorotaxane acts as a photosensitiser. However, this ferrocene-containing pseudorotaxane is the only example reported to date of a pseudorotaxane capable of reversible switching accompanied by crystal-to-crystal phase transitions as directly observed by single-crystal X-ray crystallography^41–43^. Therefore, it is essential to develop further unique pseudorotaxanes with different functional groups that allow significant dynamic motion in the single-crystal state.

Consequently, herein we report thermo- and photoresponsive dynamic pseudorotaxane crystals containing a ruthenocene moiety (complex **2**) as well as mixed crystals with various ferrocene and ruthenocene ratios (complexes **3–5** in Fig. 1a). Ru has a larger atomic radius than Fe (132.5 and 124.1 pm, respectively). Therefore, it elongates the axle molecule to provide a highly-strained structure, which is expected to facilitate significant dynamic motion in the single-crystal state (Fig. 1b).

## Results

### Thermally induced phase transition

Complex **2** was synthesised by mixing the ruthenocene-containing axle molecule and DB24C8 in dichloromethane, followed by crystallisation by diffusion of diethyl ether vapour into the pseudorotaxane solution (Fig. 1, Supplementary Figs S1–S9). Mixed crystals of complexes **3–5** containing Fe and Ru at different ratios (Fe:Ru = 7:3 for **3**, Fe:Ru = 5:5 for **4**, Fe:Ru = 3:7 for **5**) were also prepared by mixing complexes **1** and **2** at controlled molar ratios in dichloromethane solutions followed by crystallisation. Fe:Ru ratios were estimated by single-crystal X-ray crystallography based on a substitutional disorder provided by occupation by different types of atoms at the same site. These values were consistent with the mixing ratios in the synthesis.

**Figure 2:**
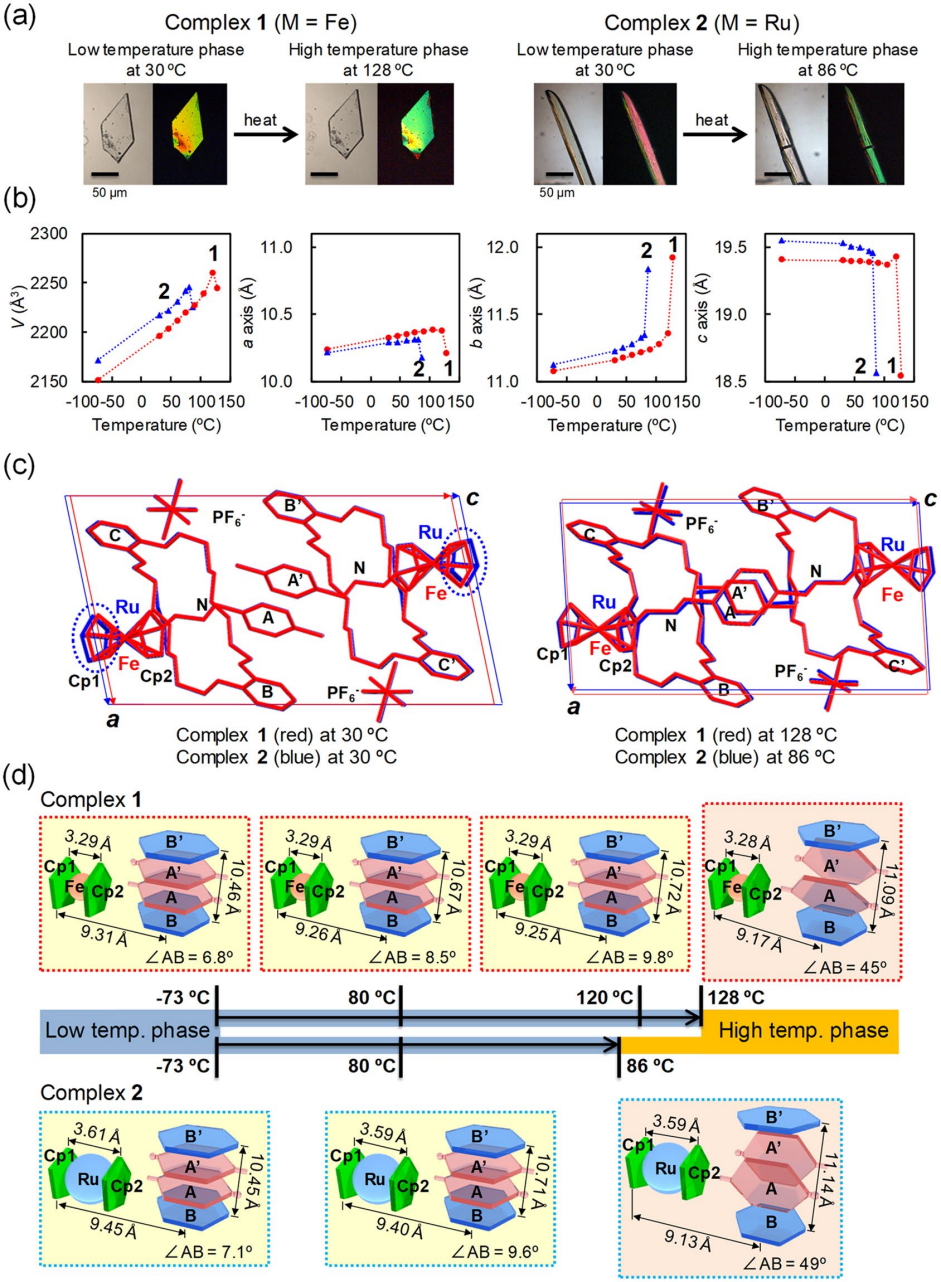
Thermal phase transition of pseudorotaxane crystals. (**a**) Optical micrographs of single crystals of complexes **1** and **2** without/with cross polarised light. (**b**) Volume and unit cell parameters of complexes **1** and **2** at different temperatures. (**c**) Overlays of the molecular structures of complexes **1** and **2** obtained by single-crystal X-ray crystallography at 30 °C, 128 °C (for complex **1**) and 86 °C (for complex **2**). See also the Supplementary Movie. (**d**) Schematic illustration of the molecular structural changes of complexes **1** and **2** on heating. Specific distances and angles between aromatic rings are depicted.

Figure 2 compares the thermally induced crystal-to-crystal phase transition behaviours of single crystals of complexes **1** ^41,42^ and **2**. Both crystals show dramatic interference colour change upon heating to 128 °C for complex **1** and 86 °C for complex **2**, accompanied by a crystal-to-crystal thermal phase transition (Fig. 2a). To investigate the structural changes, single-crystal X-ray crystallography was conducted for these complexes at different temperatures (Supplementary Tables S1–S7). Figure 2b shows the temperature dependence of the unit cell volume *V* and the lengths of the *a*, *b* and *c* axes for crystals of complexes **1** ^43^ and **2** (Supplementary Table S1). Both crystals show expansion upon heating from −73 °C to just below their phase transition temperatures with the volumetric thermal expansion coefficients *α*_v_ = 263 × 10^−6^ Å^3^ K^−1^ for complex **1** and *α*_v_ = 223 × 10^−6^ Å^3^ K^−1^ for complex **2**. These values are some of the highest values for crystals of organic materials and metal-organic complexes reported to date^44–46^. Such significant thermal expansion is likely due to their flexible interlocked structures, which are expected to provide significant stimuli-responsive mechanical effects.

Upon heating beyond the phase transition temperatures, discontinuous changes in the unit cell parameters are observed (Fig. 2b). Complex **2** has similar *a* and *b* axis lengths to complex **1** both in the low and high temperature phases, whereas the *c* axis of complex **2** is significantly longer than that of complex **1** at −73 °C and becomes similar to that of complex **1** in the high temperature phase. The longer *c* axis for complex **2** in the low temperature phase is due to the presence of the bulky ruthenocene group.

Figure 2c presents overlay drawings of the molecular structures in the unit cells for complexes **1** and **2** obtained by single-crystal X-ray crystallography, and the specific distances and angles are summarised in Fig. 2d and Supplementary Figs. S10 and S11. At low temperatures, both pseudorotaxane molecules are stabilised by an intramolecular π–π interaction between the tolyl group (aromatic ring A) of the axle molecule and the catechol group (aromatic ring B) of the cyclic molecule, and by an intermolecular π–π interaction between rings A and A’ of two axle molecules. A significant difference is observed along the *c* axis due to the presence of different metallocene groups that span the *c* axis, where the distance between the two cyclopentadienyl (Cp) groups for complexes **1** and **2** are estimated to be 3.29 and 3.61 Å, respectively, at −73 °C. Therefore, in complex **2**, the bulky ruthenocene elongates the intramolecular distance along the *c* axis, i.e. the distance between Cp1 and ring B at −73 °C is 9.45 Å, which is longer than that in complex **1** (Cp1-B = 9.31 Å) (Fig. 2d). Thus, the bulky ruthenocene distorts π–π interactions, effecting a slightly higher plane angle between rings A and B (7.1° at −73 °C and 9.6° at 80 °C) compared to that in complex **1** (6.8° at −73 °C and 8.5° at 80 °C). This distortion is one of the main reasons for the significantly lower phase transition temperature for complex **2**.

Upon heating, the plane angle between rings A and B for both complexes gradually increases, accompanied by an expansion of the crown ether cavity between rings B and B’ (Fig. 2d). These results indicate that the A-A’ pair of rings of the axle molecules are gradually activated on heating. Above the phase transition temperature, the A-A′ pair of rings eventually twist together maintaining their intermolecular π–π interaction. The plane angle between rings A and B for complex **2** significantly increases to 49°, which is higher than that of complex **1** (45°), whereas the Cp1-B distance for complex **2** (9.13 Å) becomes similar to that of complex **1** (9.17 Å) (Fig. 2d and Supplementary Fig. S10). These results suggest that the distortion along the *c* axis in the low temperature phase of complex **2** is released to provide a new distortion in the aromatic rings A, A’, B and B’ to form the more twisted axle pair A-A’ in the high temperature phase.

### Thermal properties of mixed crystals

**Figure 3:**
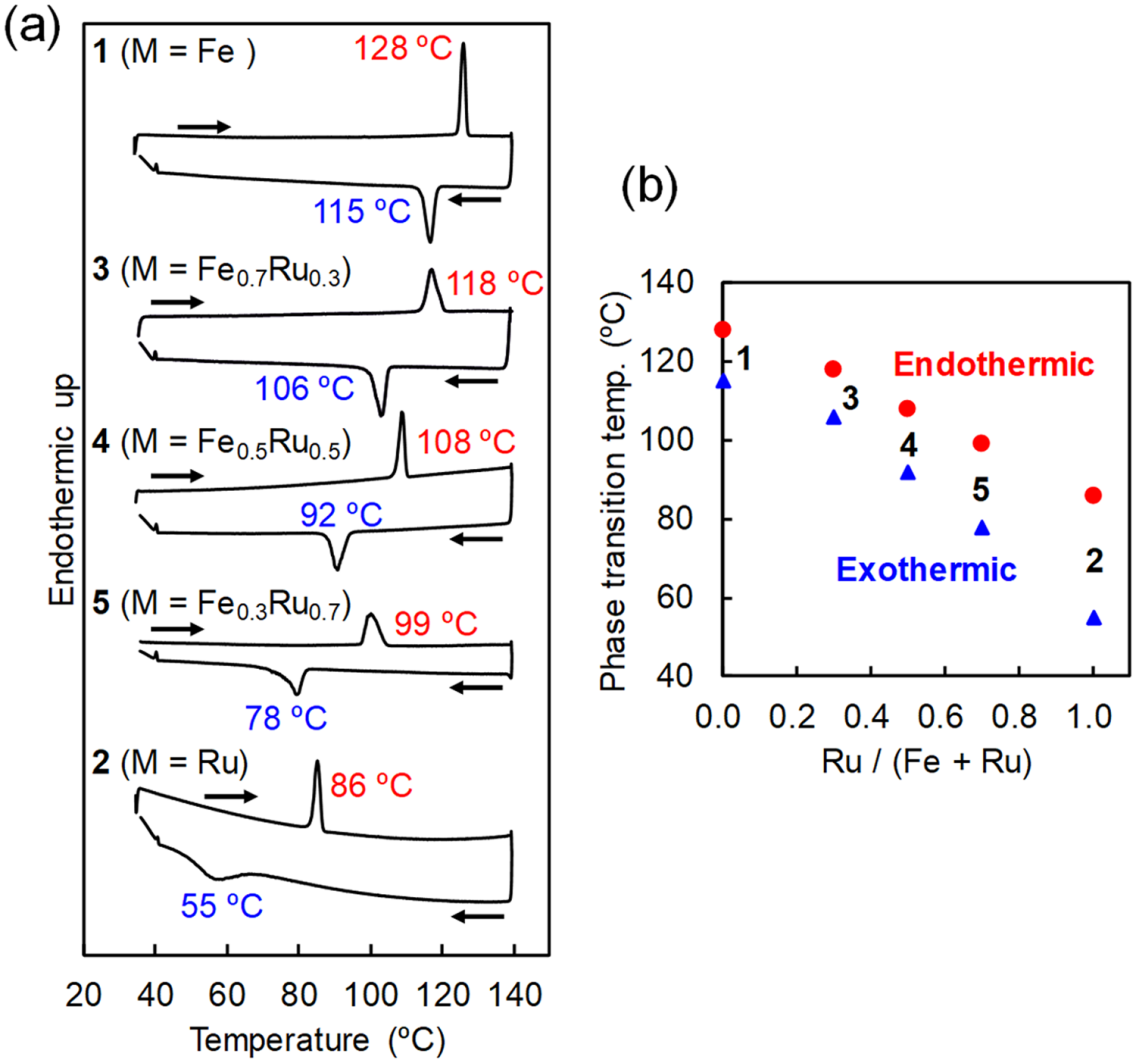
Thermal properties of mixed crystals. (**a**) DSC heating and cooling scans for complexes **1–5** at a scan rate of 5 °C min^−1^. (**b**) Plots of crystal-to-crystal phase transition temperatures vs. molar fraction, i.e. Ru/(Fe + Ru).

The large difference in the phase transition temperatures of complexes **1** and **2** is advantageous for tailoring the thermal properties of these pseudorotaxane complexes. Consequently, mixed crystals were prepared using various Fe and Ru ratios. Figure 3a shows the differential scanning calorimetry (DSC) results for complexes **1**–**5**. All crystals show endo- and exothermic peaks upon heating and cooling, respectively, accompanied by crystal-to-crystal phase transitions. The thermodynamic parameters enthalpy (*ΔH*) and entropy (*ΔS*) were estimated from the peak areas and temperatures (Supplementary Table S8). As the fraction of Ru increases, these peaks shift to lower temperatures (Fig. 3b) with slightly lower *ΔH* and *ΔS* values.

**Figure 4:**
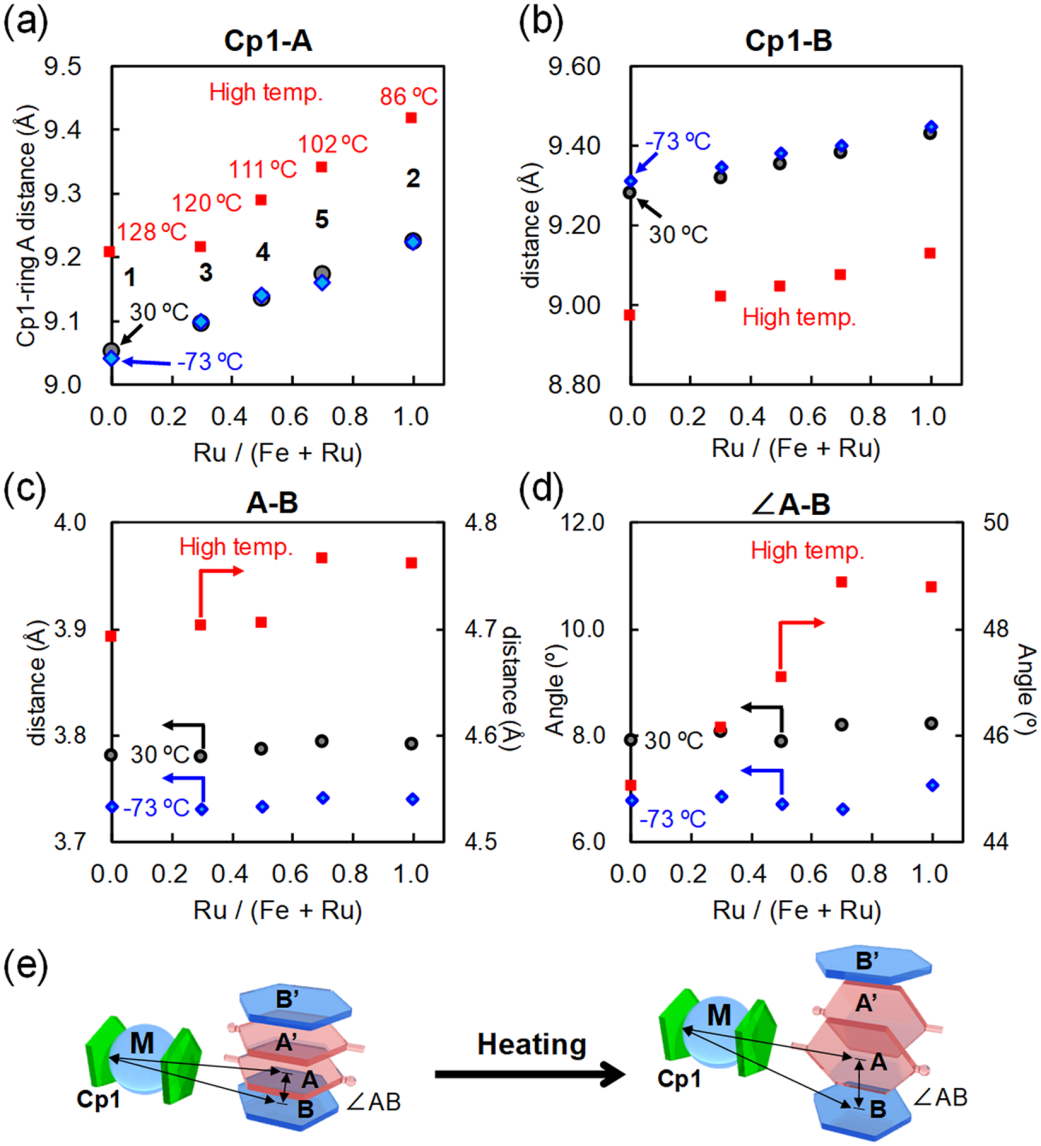
Plots of intra-/intermolecular distances and angles vs. Ru/(Fe + Ru). Distances between (**a**) Cp1 and aromatic ring A, (**b**) Cp1 and aromatic ring B, (**c**) aromatic rings A and B. (**d**) Angle between aromatic rings A and B. (**e**) Schematic illustration of the molecular structural changes of complexes **1**–**5** on heating.

This phenomenon can be explained by considering the molecular structures obtained by the single-crystal X-ray crystallography for complexes **1–5** (Supplementary Figs S12–S14 and Supplementary Tables S2–S7). Intra- and intramolecular distances and angles are plotted with the molar fraction Ru/(Fe + Ru) at −73 °C, 30 °C and above the phase transition temperatures in Fig. 4. As the fraction of Ru increases, the distances Cp1-ring A and Cp1-ring B are elongated at all temperatures due to the presence of bulky Ru (Fig. 4a and b). Upon heating above the phase transition temperatures, significant increase in Cp1-ring A and decrease in Cp1-ring B are observed accompanied by the twisting of the pair of axle molecules. Similarly, as the fraction of Ru increases, the distance and angle between aromatic rings A and B are significantly increased above the phase transition temperatures (Fig. 4c and d). Consequently, the bulky ruthenocene provides distortion in the rotaxane structure to facilitate twisting of the axle molecules, resulting in the lower phase transition temperatures, as observed in DSC (Fig. 3).

**Figure 5:**
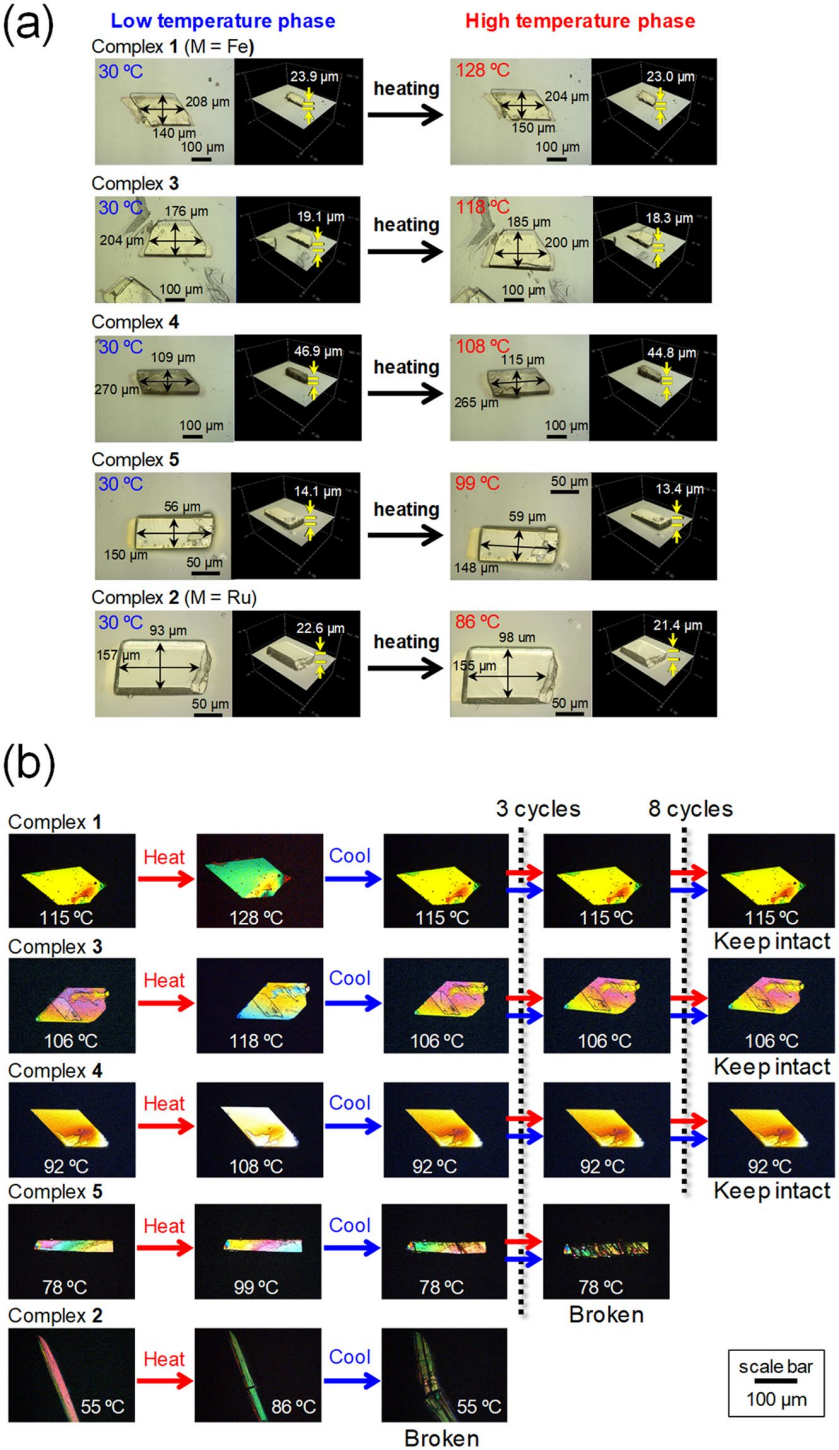
(**a**) Reflection micrographic images of crystals of complexes **1–5**. These images were observed using a confocal laser microscope. (**b**) Optical micrographs of single crystals of complexes **1**–**5** under cross polarised light. Crystals were repeatedly heated beyond the phase transition temperatures and cooled.

To investigate macroscopic changes of the crystals accompanied by the phase transition, reflection micrographic images for crystals of complexes **1–5** were observed using a confocal laser microscope at 30 °C and above their phase transition temperatures (Fig. 5a). The crystal of complex **2** shows significant anisotropic deformation upon phase transition. The horizontal length, vertical length and thickness are changed from 157 to 155 μm (−1%), from 93 to 98 μm (+5%) and from 22.6 to 21.4 μm (−6%), respectively. This macroscopic crystal deformation is clearly related to the changes at the molecular level as observed by single-crystal X-ray crystallography, i.e. the *a*, *b* and *c* axes for complex **2** change by −1%,+5% and −5%, respectively (Fig. 2b and Supplementary Table S1).

Crystals of all the complexes undergo similar crystal deformations upon thermal phase transition. However, the crystals of complexes **2** and **5** are fractured by the phase transition, particularly in the cooling process (Fig. 5b and supplementary Fig. S15). This is probably because of the relatively weak intermolecular interaction along the fracture direction formed by stacking of pseudorotaxane molecules. This interaction is not durable to the dramatic size change during the phase transition, leading to quasi-reversibility. In terms of the reversibility of the phase transition in pseudorotaxane crystals for switching operations, the use of complex **1** is beneficial to the preparation of mixed crystals due to its durability to the phase transition. Indeed, the reversibility of the thermal phase transition of the crystals is substantially improved in the mixed crystals, particularly in complexes **3** and **4** (Fig. 5b).

### Photoinduced structural changes

**Figure 6:**
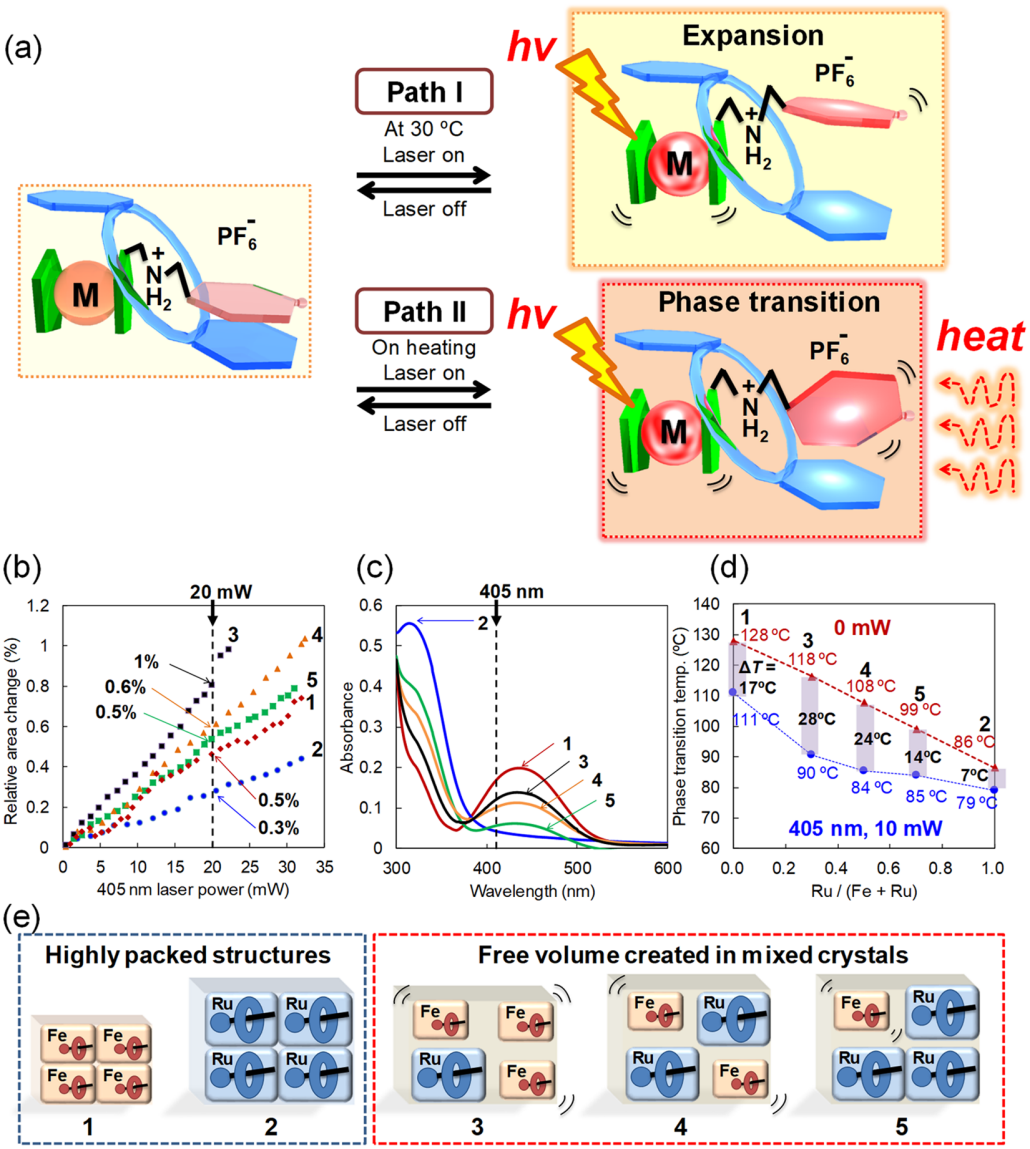
Photoresponsive behaviours of pseudorotaxane crystals. (**a**) Schematic of photoinduced structural changes. Pseudorotaxane is activated by laser irradiation, leading to expansion of the crystal at 30 °C (Path I) and crystal-to-crystal phase transition at high temperatures (Path II). (**b**) Laser-power dependence of the change in the relative area of the (001) facet of the single crystal induced by focused 405 nm laser irradiation. (**c**) UV-visible absorption spectra of mixed crystals in chloroform solutions. (**d**) Plots of phase transition temperature vs. Ru/(Fe + Ru) with/without 10 mW 405 nm laser irradiation. (**e**) Illustration of packing structures for pseudorotaxanes in the crystal state. Free space can be created by the presence of ruthenocene in the mixed crystals. See also the Supplementary Movie.

Photoinduced switching of pseudorotaxane crystals was investigated. As shown in the schematic illustration in Fig. 6a, expansion/contraction of the crystals can be controlled by turning on/off laser irradiation at 405 nm or 445 nm at 30 °C (Path I). Furthermore, crystal-to-crystal thermal phase transition can be triggered by laser irradiation upon heating (Path II). These macroscopic crystal behaviours were observed using an optical microscope with/without cross polarised light under controlled temperatures.

Figure 6b and Supplementary Fig. S16a show the laser-power dependence of the change in the relative area of the (001) facet, which is the top face of the single crystals, at 405 nm and 445 nm, respectively (Fig. 6a Path I). The magnitude of crystal expansion is significantly dependent on the power and wavelength of the laser. The crystal of complex **3** shows the highest magnitude of expansion, i.e.+1%, followed by **4** (+0.6%), **5** (+0.5%), **1** (+0.5%) and **2** (+0.3%), under 20 mW of 405 nm laser irradiation (Fig. 6b), whereas the order for 20 mW of 445 nm laser irradiation is **3** (+0.8%), **1** (+0.7%), **4** (+0.5%), **5** (+0.5%) and **2** (<+0.1%) (Supplementary Fig. S16a).

Figure 6c shows the UV-visible spectra of complexes **1**–**5** in chloroform solutions. Complexes **1** and **2** exhibit absorption maxima at 440 and 320 nm, respectively, which are assigned to metal-to-ligand charge transfer (MLCT) transitions of the ferrocenyl and ruthenocenyl group, respectively. Hence, the order of absorption intensity at 405 nm and 445 nm is **1**, **3**, **4**, **5** and **2**, which is also the order of ferrocenyl group content. Interestingly, this order is inconsistent with the magnitude of the expansion of the crystals, suggesting that the expansion efficiency is related to not only the photo absorption coefficient but also other parameters such as molecular packing, as discussed later.

Furthermore, the crystal-to-crystal phase transition temperatures were estimated with/without 10 mW 405 or 445 nm laser irradiation, based on observations using a cross polarised microscope, and these temperatures are plotted vs. Ru/(Fe + Ru) (Fig. 6a Path II, Fig. 6d and Supplementary Fig. S16b). The transition temperatures of all the complexes decrease upon laser irradiation. Among them, the mixed crystals of complexes **3** and **4** exhibit particularly large temperature drops (Δ*T* = 28 °C for complex **3** and Δ*T* = 24 °C for complex **4**) under 405 nm laser irradiation.

The photoinduced expansion/contraction and the decrease in the phase transition temperatures can be accounted for by the photo-thermal conversion of the crystals. In our previous study, the effect of laser irradiation on the surface temperature of the pseudorotaxane crystals was analysed through thermographic measurement, which showed that the crystal temperature rapidly increased by 10–15 °C by 445 nm laser irradiation^43^. This causes the photoactivation of the ferrocenyl group in the pseudorotaxanes, leading to the elongation of the crystal lattice in 3D as well as the photo-assisted thermal phase transitions.

To rationalise the higher dynamics of the mixed crystals than those of the pure crystals, an illustration of the simple packing models of the pseudorotaxanes is proposed in Fig. 6e. The crystals of complexes **1** and **2** have highly packed structures compared to those of the mixed crystals. Therefore, the motions of the pseudorotaxane molecules therein are lessened. Conversely, the addition of the bulky ruthenocene complex disrupts the packing in the mixed crystals of complexes **3–5**, and as a result, the mixed crystals exhibit relatively flexible packing and larger free volumes. Hence, the pseudorotaxane molecules with the less bulky ferrocenyl group exhibit higher dynamics. In addition, because the ferrocenyl group exhibits high absorbance at 405 and 445 nm, crystals of complex **3** with the high ferrocenyl composition ratio show the most significant dynamics upon laser irradiation at 405 and 445 nm in all pseudorotaxanes.

### Photomechanical conversion

**Figure 7:**
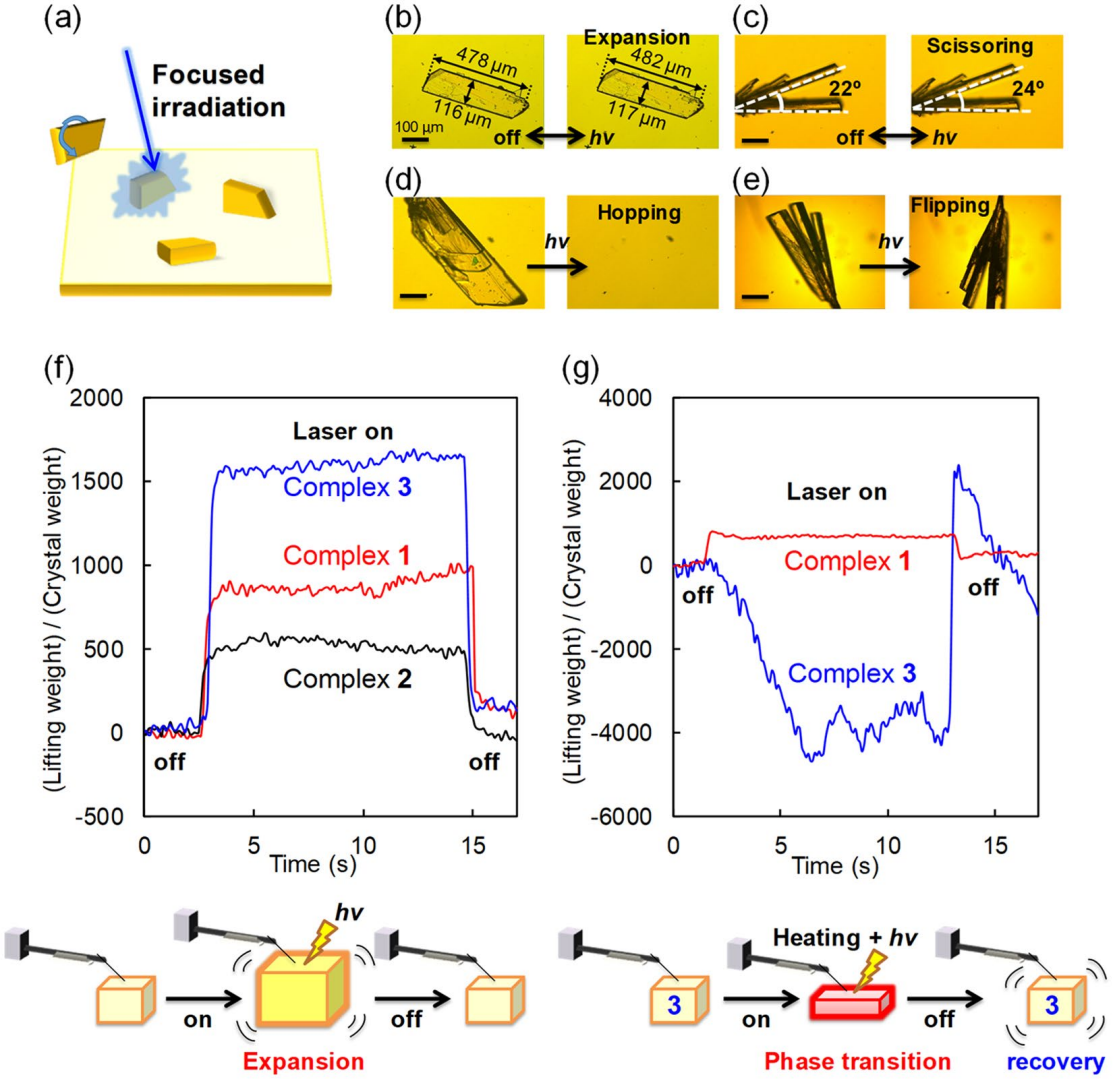
Photosalient effects. (**a**) Illustration of photosalient effect triggered by focused laser irradiation. (**b**) Photoinduced expansion of a crystal of complex **3** controlled by 405 nm laser irradiation (14 mW) at 30 °C. (**c**) Photoinduced scissoring motion of crystals of complex **3** controlled by 405 nm laser irradiation (16 mW) at 30 °C. (**d**) Hopping of a crystal of complex **3** triggered by 405 nm laser irradiation (25 mW) at 30 °C. (**e**) Flipping of crystals of complex **3** triggered by 405 nm laser irradiation (25 mW) at 30 °C. (**f**) Time dependence of lifting/crystal weight ratio of complexes **1–3** controlled by 405 nm laser irradiation (50 mW) at 30 °C. (**g**) Time dependence of lifting/crystal weight ratio of complexes **1** and **3** controlled by 405 nm laser irradiation (12 mW) at 85 °C. Illustration of the force detection is depicted. Scale bars represent 100 μm. See also the Supplementary Movie.

Next, the photoinduced deformations of the pseudorotaxane crystals are used for demonstrations of photomechanical conversions (Fig. 7 and Supplementary Fig. S17). Figure 7a–e shows illustrations and optical micrographs of various photo-triggered salient effects of crystals of complex **3**. Crystals of complex **3** were selected because it shows the highest sensitivity and good reversibility in the switching operations discussed above. The plate-shaped crystal shows reversible size expansion/contraction controlled by 405 nm irradiation with a weak 14 mW laser at 30 °C (Fig. 7b). In addition, a cluster of rod-shaped crystals exhibits a reversible scissoring motion controlled by 405 nm laser irradiation at a power of 16 mW (Fig. 7c). These motions are regular and the crystal integrity is completely retained. Furthermore, hopping of the crystals over a long distance and flipping over a short distance are triggered by 405 nm laser irradiation at a relatively strong laser power of 25 mW at 30 °C (Fig. 7d and e). These motions are drastic and irregular (i.e. stochastic). Therefore, they are probably induced by the crystal-to-crystal phase transition. In fact, complex **3** undergoes crystal-to-crystal phase transition even at 30 °C under 405 nm irradiation at a high laser power of 25 mW (Supplementary Fig. S18). After the hopping and flipping, the crystals still preserve their macroscopic integrity.

The forces accompanied by the photoinduced crystal expansion and phase transition were measured at 30 and 85 °C for crystals of complexes **1–3** using a microforce analyser comprising two gauges on a cantilever (Fig. 7f and g and Supplementary Fig. S19). A 13 μg crystal of complex **3** provides 20 mg force when irradiated by 50 mW of 405 nm laser at 30 °C. This value corresponds to 1,600-times the crystal weight itself. Under the same observation condition, complex **3** provides the highest lifting weight ratio of the complexes analysed, followed by complexes **1** and **2**. This order is consistent with the expansion sensitivity to 405 nm laser irradiation (Fig. 6b).

Conversely, upon heating at 85 °C under 12 mW of 405 nm laser irradiation, a negative force is observed for 2.5 μg of a crystal of complex **3**. This is due to a decrease in the crystal thickness (*c* axis, Fig. 2b) upon phase transition, which is completed within only 4 ms (Supplementary Fig. S20). When the laser is turned off, the crystal immediately returns to its original form on the same timescale (4 ms, Supplementary Fig. S20), providing a high lifting weight of 16 mg that corresponds to a weight ratio of 6,400-times the crystal weight. For comparison, complex **1** was also irradiated under the same conditions. However, complex **1** exhibits expansion only because it has a much higher phase transition temperature than complex **3**. The observed weight ratio for the phase transition of complex **3** is 4 times of magnitude higher than that previously observed for the expansion of complex **1** (a lifting/crystal weight ratio of ca. 1600) under 445 nm laser irradiation^43^, and 1–2 orders of magnitude higher than those previously reported for photochromic azobenzene polymer films^47,48^, diarylethene crystals^23,49^ or hydrogels with electro-triggered mobility^50^ and polymer composite actuators driven by hydro gradients^51^.

## Conclusion

We have developed metallocene-containing dynamic pseudorotaxane crystals that respond to thermo- and photostimuli, transducing the incident energy into mechanical motions. These pseudorotaxanes are composed of multiple functional moieties: the ring molecule of the dibenzo crown ether acts as a flexible host with size and motion adjustability; the aromatic ring at one end of the axle molecule exhibits mechanical twisting dynamics, and the ferrocene at the other end of the axle molecule acts as a photosensitiser under 405 nm laser irradiation. Furthermore, substituting the ferrocene moiety with a bulky ruthenocene complex facilitates the twisting of the aromatic ring of the axle molecule. The structural changes of the pseudorotaxanes have been precisely observed by single-crystal X-ray crystallography, providing a detailed rationale and a strategy for the molecular design of interlocked molecules that accumulate molecular motion and express them as macroscopic dynamics in the crystal state. We expect that such unique dynamic interlocked molecules in the crystal state can be applied as molecular actuators and switches in stimuli-responsive mechanical components.

## Methods

### General methods

Synthesis and crystallization of the pseudorotaxane were performed on the basis of the method described in the literature^52^. Ruthenocene, sodium borohydride (NaBH_4_) and DB24C8 were purchased from Sigma-Aldrich. Anhydrous DMF, toluene, MeOH and THF were purchased from Aldrich in Sure/Seal container and were stored under N_2_ gas. 4-Methylbenzylamine was purchased from Alfa Aesar. ^1^H NMR spectra were record on Varian-Unity INOVA-500 spectrometer. The chemical shifts were referenced with respect to CHCl_3_ (δ_H_ = 7.24) and CD_2_HCN (δ_H_ = 1.93) for ^1^H as internal standards. Elemental analysis was carried out with a CHN-O-Rapid elemental analyzer (Foss. Heraeus, Germany). Fast atom bombardment mass spectra (FAB-MS) were obtained with Micromass Trio 2000 using 3-nitrobenzyl alcohol as the matrix. DSC curves were recorded by a Perkin Elmer Diamond DSC instrument using heating and cooling rates of 10 °C min^−1^. UV-visible absorption spectra were measured using a JASCO V-630 spectrophotometer.

### Synthesis of ruthenocenylmethyl(4-methylphenyl)ammonium axle molecule

*p*-Xylylamino-methylruthenocene (163.6 mg, 0.45 mmol) in 6 N HCl aq. (10 mL) was treated with ultrasonication for a few minutes to prepare a suspension. This was then stirred at a room temperature for 1 h. The residual brown powder was washed with water (20 mL) then dried in vacuum. To a suspension of [ruthenocenylmethyl(4-methylphenyl) ammonium]^+^ Cl^−^ in acetone (150 mL), NH_4_PF_6_ (93.5 mg, 0.58 mmol) was added, and the mixture was stirred at a room temperature for 1 h. The precipitate was collected by filtration and dried in vacuum to give [ruthenocenylmethyl(4-methylphenyl) ammonium]^+^(PF_6_)^−^ (133.4 mg, 58%) as a light brown powder. ^1^H NMR spectrum (500 MHz, CD_3_CN, r.t.): δ 2.32 (s, 3 H, Me), 3.78 (br, 2 H, CH_2_), 4.10 (br, 2 H, CH_2_), 4.58 (s, 5 H, C_5_H_5_), 4.62 (m, 2 H, C_5_H_4_), 4.75 (m, 2 H, C_5_H_4_), 7.26 (d, 2 H, C_6_H_4_, *J* = 7.5 Hz), 7.32 (d, 2 H, C_6_H_4_, *J* = 7.5 Hz) (Supplementary Fig. S2). ^13^CNMR (500 MHz, CD_3_CN): δ 21.25 (Me), 47.76, 51.83 (CH_2_), 72.14 (C_5_H_5_), 72.31, 73.33, 80.89 (C_5_H_4_), 128.45, 130.70, 131.01, 140.93 (C_6_H4) (Supplementary Fig. S3). HRMS (FD) calcd. for C_19_H_22_NRu [M + H]^+^ (*m*/*z*): 366.0790, found: 366.0780 (error 2.7 ppm) (Supplementary Fig. S5). Anal. Calcd. for C_19_H_22_NF_6_RuP: C, 44.70; H, 4.30; N, 2.75. Found: C, 44.53; H, 4.75; N, 2.85.

### Preparation of [ruthenocenylmethyl(4-methylphenyl)ammonium·DB24C8]^+^(PF_6_)^−^ rotaxane crystal

[Ruthenocenylmethyl(4-methylphenyl)ammonium]^+^(PF_6_)^−^ (0.028 mmol, 14.4 mg) and DB24C8 (0.028 mmol, 14.3 mg) were dissolved in CH_2_Cl_2_. This solution was then placed in Et_2_O vapour atmosphere at a room temperature for 24 h to give light yellow crystals of [ruthenocenylmethyl(4-methylphenyl)ammonium ·DB24C8]^+^(PF_6_)^−^ (19.5 mg, 0.020 mmol, 73% yield). ^1^H NMR spectrum (500 MHz, CD_3_CN, r.t.): δ 2.11–2.13* (s, 3 H, Me), 3.55–3.70* (m, 8 H, OCH_2_), 3.70–3.90* (m, 8 H, OCH_2_), 4.04–4.21* (m, 8 H, OCH_2_), 4.19 (m, 2 H, NCH_2_), 4.33 (s, 5 H, Cp), 4.38 (s, 2 H, Cp), 4.58 (m, 2 H, NCH_2_), 6.87–6.95* (m, 8 H, C_6_H_4_ (host)), 7.15 (d, 2 H, C_6_H_4_, *J* = 8 Hz) (Supplementary Fig. S8). The peaks with asterisks are significantly overlapped with other signals. The complex/uncomplex ratio in CD_3_CN solution is determined to be 1.29 by the comparison of the integrated peak area of cyclopentadienyl protons and methylene protons. ^13^CNMR (500 MHz, CD_3_CN): δ 21.24 (uc, Me), 30.88 (c, Me) 47.71, 51.77 (uc, CH_2_), 49.24, 52.77 (c, CH_2_), 71.94 (uc, C_5_H_5_), 71.85 (c, C_5_H_5_), 72.27, 73.30, 80.94 (uc, C_5_H_4_), 72.12, 73.22, 81.10 (c, C_5_H_4_), 129.98, 130.22, 130.96, 140.84 (uc, C_6_H4), 128.95, 130.22, 130.67, 139.83 (c, C_6_H4), 69.06, 69.75, 70.27, 71.18, 71.27, 71.56 (OCH_2_), 113.67, 115.33, 122.28, 122.52, 148.72, 149.77 (C_6_H_4_) (Supplementary Fig. S9). HRMS (FD) calcd. for C_43_H_54_NO_8_Ru [M + H]^+^ (*m*/*z*): 814.2887, found: 814.29091 (error 1.7 ppm) (Supplementary Fig. S7). Anal. Calcd. for C_43_H_54_NO_8_F_6_RuP: C, 53.80; H, 5.63; N, 1.46. Found: C, 53.82; H, 5.67; N, 1.34.

### Single-crystal X-ray crystallography

Single-crystal X-ray crystallography was performed for the pseudorotaxane crystals (Bruker, APEX DUO, Dual Wavelength System, 60 Hz). An APEX II 4 K CCD Detector was used for data collection, and the corresponding Bruker APEX II software was used for data collection and reduction. An OXFORD, Cryostream 800 Plus seamless sample cooling and heating system was used to control the measurement temperature. The structures were solved using SHELXL-2014/6.

### Optical microscopy

A microscope (Olympus, BX51) equipped with a temperature-controlled stage (TMS 92 and HMS600, both from Linkam Co.) was used to monitor the crystal-to-crystal phase transitions. A crystal was placed on the stage, and the stage was heated or cooled a rate of 2 °C min^−1^. The Imagesource DFK 51AU02 charge-coupled device (CCD) camera with a video rate of 12 fps was used to capture the images and movies of a crystal. A confocal laser microscope with a 50X objective lens (Keyence VK-9500) was used to measure the crystal dimensions.

### Photomechanical measurement

Continuous-wave 405-nm and 445-nm diode pump solid state lasers (TAN-YU, T405F200 and LSR445FP-1W) were used in this experiment. A multiple-optical-fibre cable was connected as an output light source and the light illuminated from the source was aligned by a SMA 905 collimator. The power of the 405 nm or 445 nm laser beam passing through the objective lens was tuned by adjusting the amperage from the controller, and was measured using a power meter (OPHIR, Nova II P/N7Z01550). After passing through a 20X objective lens with numerical aperture (NA) = 0.25, the focused laser beam spot diameter was approximately 1 μm. The crystals were observed using the optical microscope (Olympus, BX51) equipped with the Image source DFK 51AU02 CCD camera or pco.dimax S1 high speed CMOS camera (1008^2^ pixel, 1000 fps) with CamWare V3.17 software to capture the images and movies of the crystals.

### Force measurement

A microforce detector composed of a cantilever attached to two strain gauge on both sides (CHIEF SI, μ-force) and the Bridge DAQ software were used to measure the lifting weight of the single crystals of pseudorotaxane complexes. During the measurement, all equipments were placed on a vibration isolation table to prevent any possible vibration. A continuous-wave 405-nm laser (TAN-YU, T405F200) was used with an optical microscope (Olympus, BX51) with a 4X objective lens (NA = 0.1). Temperature of crystals were controlled using TMS 92 and HMS600 (Linkam Co.).

### Data availability

Data supporting the findings of this study are available within the article (and its Supplementary Information files) and from the corresponding author on reasonable request. Crystallographic data have been deposited at the CCDC and copies can be obtained on request, free of charge, by quoting the publication citation and the deposition numbers 1546042-1546057 and 1551312.

## Electronic supplementary material

Supplement CIF files

Supplmentary Information

Supplmentary Movie
