# Supplementary material for: Dynamic Pseudorotaxane Crystals Containing Metallocene Complexes
Source: Sci Rep. 2017 Oct 27;7:14195. doi: 10.1038/s41598-017-14505-7 (PMC5660174; doi:10.1038/s41598-017-14505-7)
Supplement: Supplementary file 1 — Supplement CIF files [file 41598_2017_14505_MOESM1_ESM.zip › 168049_1_supp_5173054_rwjhhr.pdf]

Supplementary Information for

Dynamic Pseudorotaxane Crystals Containing Metallocene Complexes

Kai-Jen Chen[†], Pei-Lin Chen[‡] and Masaki Horie *[†]

†*Department of Chemical Engineering, National Tsing Hua University, 101, Sec. 2, Kuang-Fu Road, Hsinchu, 30013,*

Taiwan

‡*Instrumentation Center, National Tsing Hua University, 101, Sec. 2, Kuang-Fu Road, Hsinchu, 30013, Taiwan*

Correspondence Address
Dr. Masaki Horie
Department of Chemical Engineering, National Tsing-Hua University
101, Sec. 2, Kuang-Fu Road, Hsin-Chu, 30013 Taiwan
E-mail: mhorie@mx.nthu.edu.tw

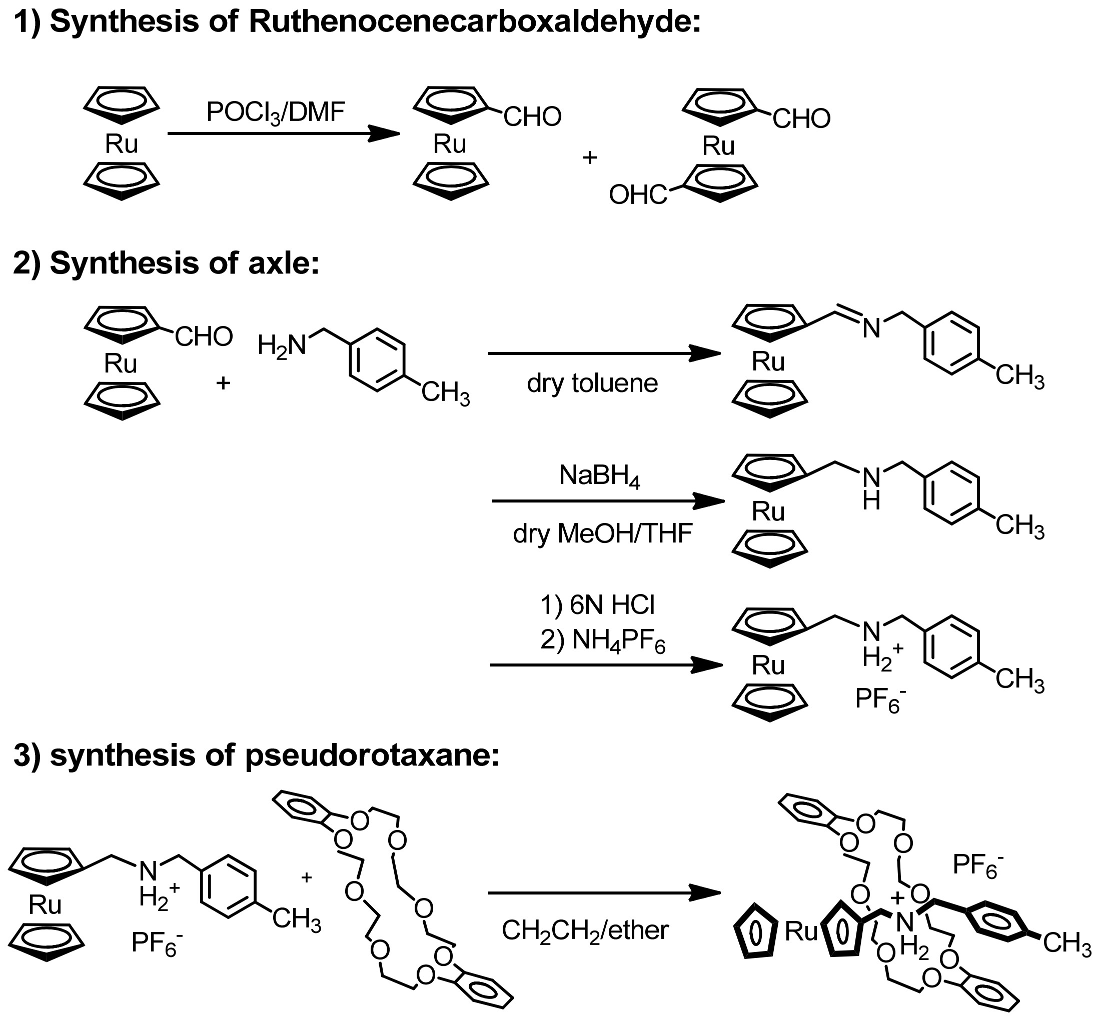

Supplementary Figure S1. Synthesis of complex **2**

Synthesis of Ruthenocenecarboxaldehyde:

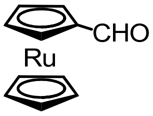

Vilsmeier–Haack reaction was used to synthesise ruthenocenecarboxaldehyde. In a Schlenk tube, $POCl_3$ (1.86 mL, 19.8 mmol) was added into DMF (1.55 mL, 19.6 mmol) at 0 ºC and stirred for 30 min. $POCl_3$/DMF solution was slowly added into ruthenocene (800 mg, 3.45 mmol) in 10 mL CH_2Cl_2 and the mixture was stirred at 80 ºC for 2 h. The mixture was cooled to a room temperature, and was slowly poured into 80 mL water. After stirring for 30 min, the mixture was partitioned between 1N NH_4Cl aq. (100 mL) and CH_2Cl_2 (100 mL). The organic extract was dried over $MgSO_4$, filtered, and concentrated under reduced pressure to give crude product. This was then purified by column chromatography (SiO_2/CH_2Cl_2) and dried in vacuum to give ruthenocene carboxaldehyde (170 mg, 19%) as a golden yellow solid. ^{1}H NMR spectrum (500 MHz, $CDCl_3$, r.t.): δ 4.63 (s, 5H, C_5H_5), 4.84 (m, 2H, C_5H_4), 5.07 (m, 2H, C_5H_4), 9.71 (d, 1H, CHO, J = 7.5 Hz).

Synthesis of *p*-xylyliminomethylruthenocene:

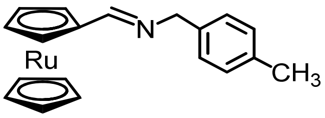

Ruthenocenecarboxaldehyde (300 mg, 1.154 mmol) and 4-ethylbenzylamine (0.147 mL, 1.154 mmol) were dissolved in dry toluene (17.5 mL) and stirred at a room temperature for 18 h. The solvent was removed under reduced pressure to provide *p*-xylyliminomethylruthenocene (414 mg, 99%) as a light brown solid. ^{1}H NMR spectrum (500 MHz, $CDCl_3$, r.t.): δ 2.31 (s, 3H, Me), 4.54 (s, 5H, C_5H_5), 4.53 (m, 2H, C_5H_4), 4.67 (s, 2H, NCH_2), 5.01 (m, 2H, C_5H_4), 7.10 (d, 2H, C_6H_4, J = 7.5 Hz), 7.11 (d, 2H, C_6H_4, J = 7.5 Hz), 8.03 (s, 1H, CH).

Synthesis of *p*-xylylaminomethylruthenocene:

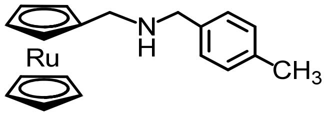

p-Xylyliminomethylruthenocene (386 mg, 1.06 mmol) was dissolved in dry THF/MeOH (24 mL:40 mL). $NaBH_4$ (40.9 mg, 1.08 mmol) was added to the solution, and stirred at a room temperature for 2 h. Further portion of $NaBH_4$ (40.9 mg, 1.08 mmol) was added to the reaction mixture. After stirring for 12 h, the reaction was quenched by addition of 1N HCl aq. (2 mL). The solvent was partitioned between 1N KOH aq. (10 mL) and CH_2Cl_2 (10 mL). The organic extract was dried over $MgSO_4$, filtered, and concentrated under reduced pressure to give *p*-xylylaminomethylruthenocene (376.6 mg, 96%) as dark brown oil. ^{1}H NMR spectrum (500 MHz, $CDCl_3$, r.t.): δ 2.28 (s, 3H, Me), 3.61 (br, 2H, CH_2), 3.89 (br, 2H, CH_2), 4.49 (s, 5H, C_5H_5), 4.53 (m, 2H, C_5H_4), 4.80 (m, 2H, C_5H_4), 7.16 (d, 2H, C_6H_4, J = 7.5 Hz), 7.40 (d, 2H, C_6H_4, J = 7.5 Hz).

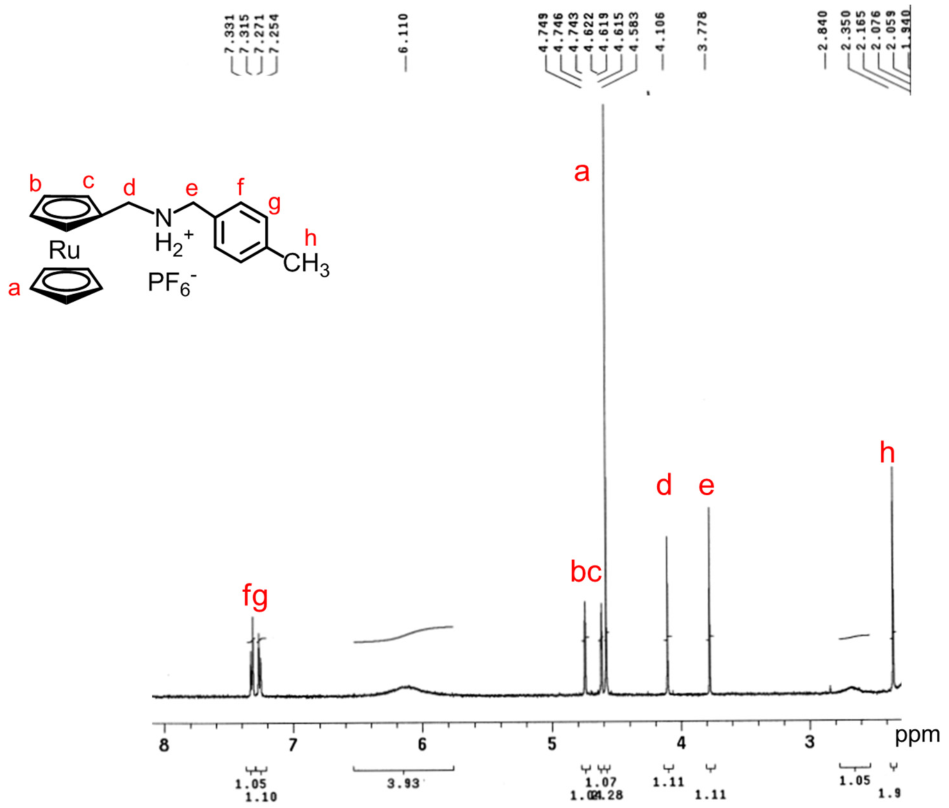

Supplementary Figure S2. ^{1}H NMR spectrum (500 MHz, CD_3CN) of [ruthenocenylmethyl (4-methylphenyl) ammonium]$^+$$(PF_6)^-$.

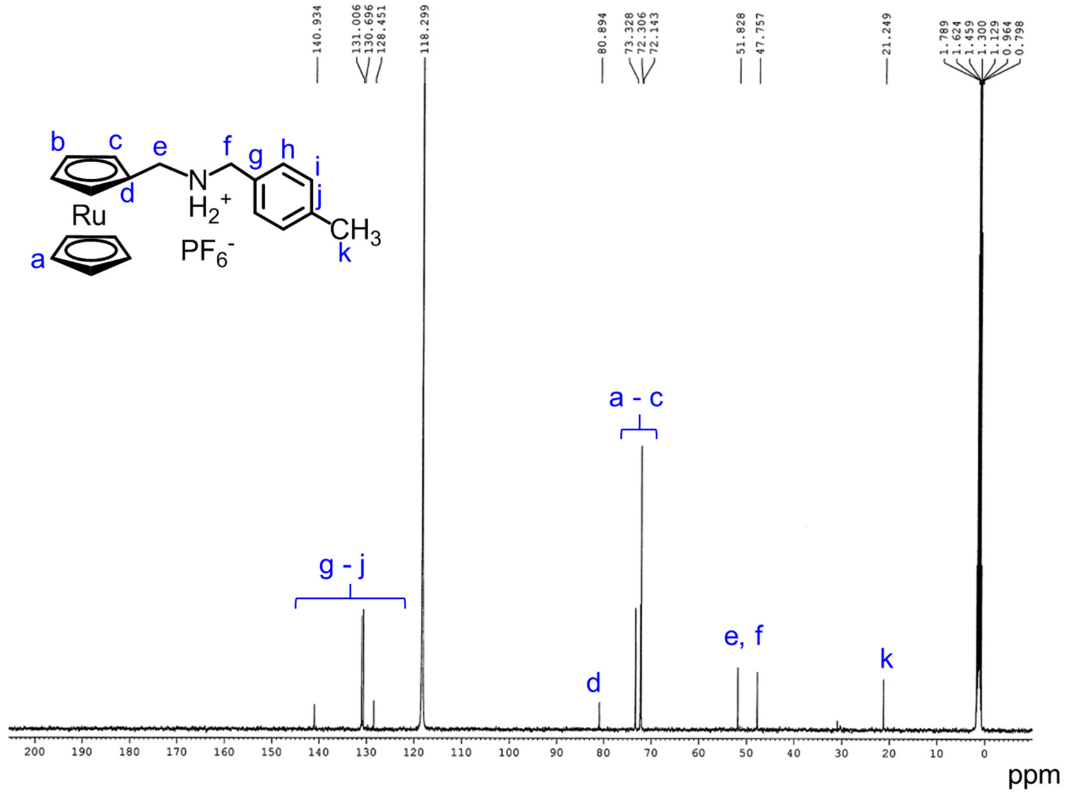

Supplementary Figure S3. ^{13}C NMR spectrum (125 MHz, CD_3CN) of [ruthenocenylmethyl (4-methylphenyl) ammonium]$^+$$(PF_6)^-$.

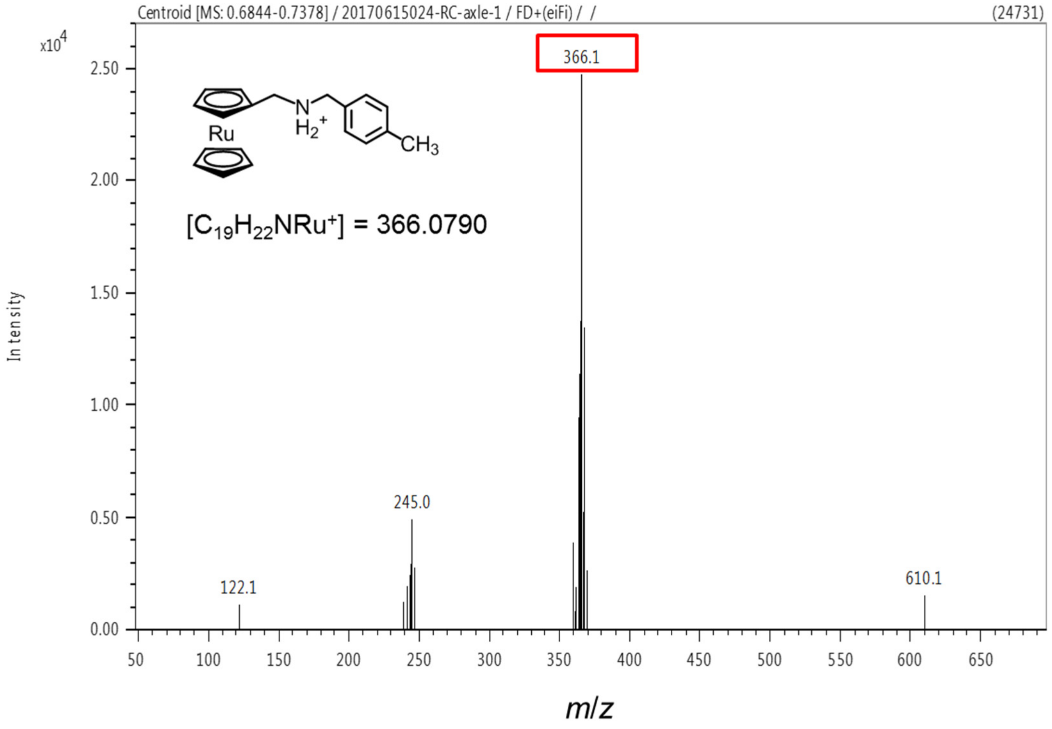

Supplementary Figure S4. LRFD mass spectra of [ruthenocenylmethyl(4methylphenyl) ammonium]$^+$(PF$_6$)$^-$. The main peak at m/z = 366.1 corresponds to [ruthenocenylmethyl(4-methylphenyl) ammonium]$^+$ ([$C_{19}H_{22}NRu^+$] =366.0790, error 57 ppm).

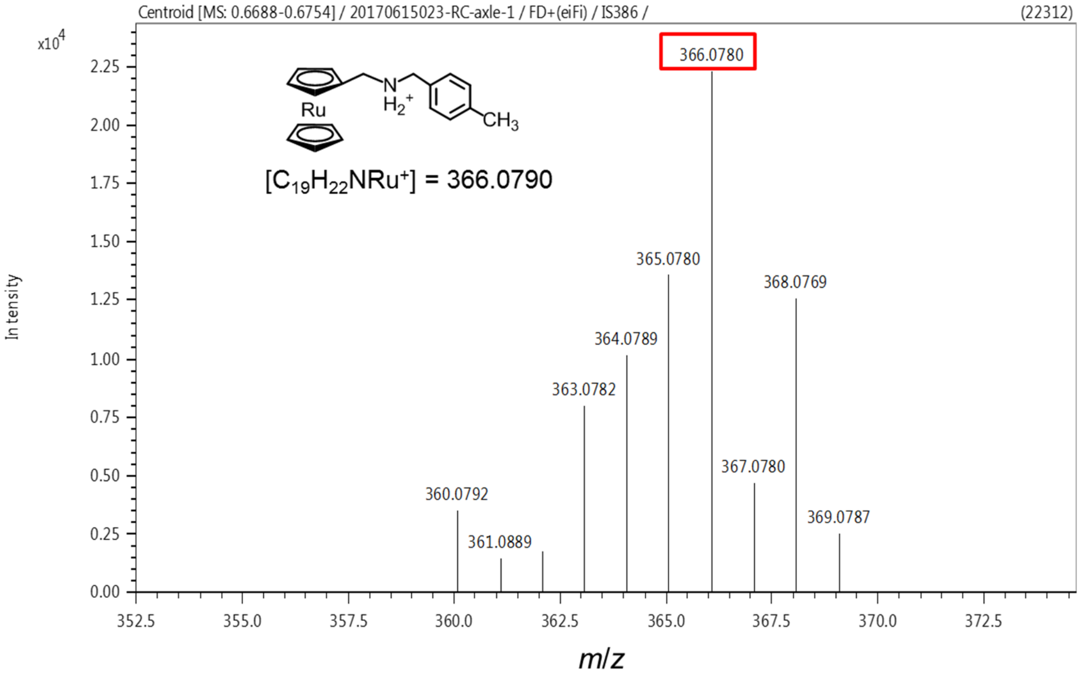

Supplementary Figure S5. HRFD mass spectra of [ruthenocenylmethyl(4methylphenyl) ammonium]$^+$(PF$_6$)$^-$. The main peak at m/z = 366.0780 corresponds to [ruthenocenylmethyl(4-methylphenyl)ammonium]$^+$ ([$C_{19}H_{22}NRu^+$] =366.0790, error 2.7 ppm).

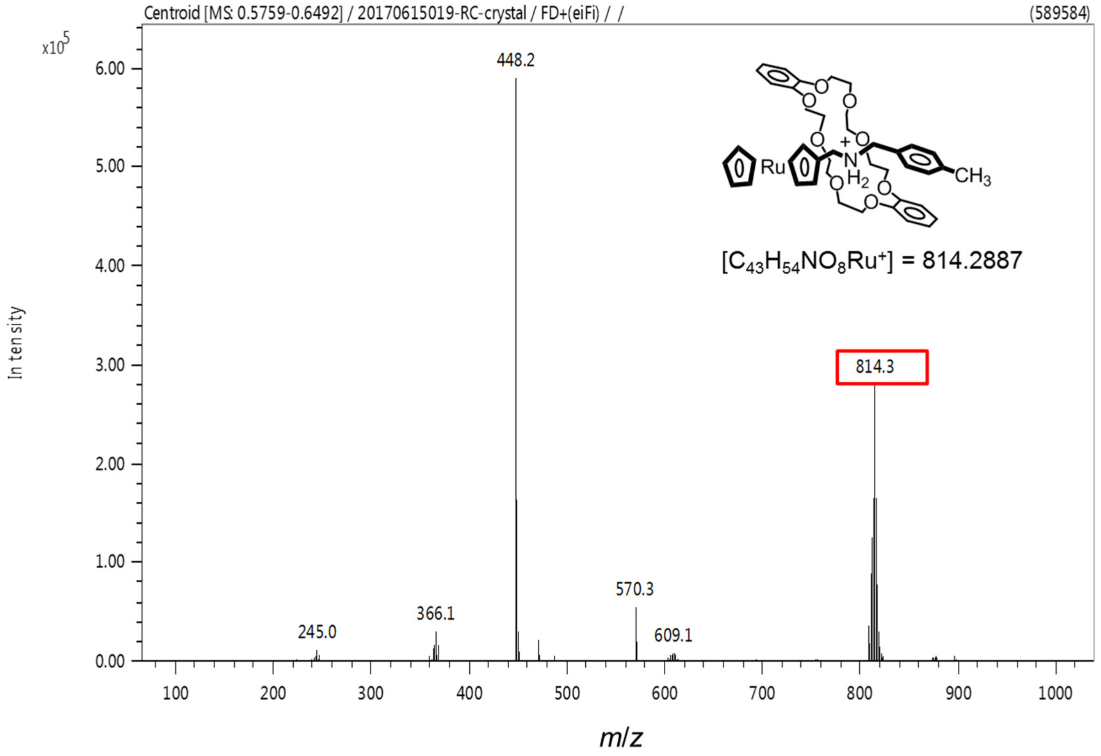

Supplementary Figure S6. LRFD mass spectra of $[Rc{\cdot}DB24C8]^+(PF_6)^-$. The main peak at *m*/*z* = 814.3 corresponds to $[Rc{\cdot}DB24C8]^+(PF_6)^-$. ($[C_{43}H_{54}NO_8Ru^+]$ =814.2887, error 14 ppm).

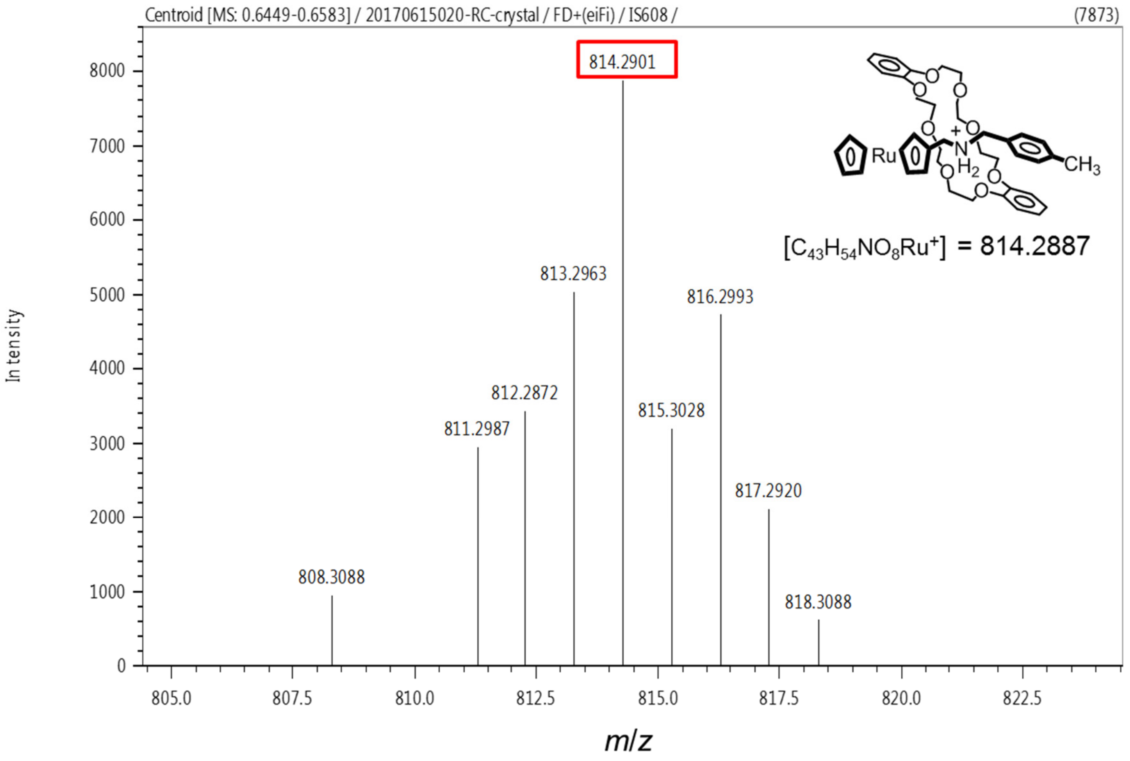

Supplementary Figure S7. HRFD mass spectra of $[Rc{\cdot}DB24C8]^+(PF_6)^-$. The main peak at *m*/*z* = 814.2901 corresponds to $[Rc{\cdot}DB24C8]^+(PF_6)^-$. ($[C_{43}H_{54}NO_8Ru^+]$ =814.2887, error 1.7 ppm).

The isolated crystals of [ruthenocenylmethyl(4-methylphenyl)ammonium·DB24C8]$^+$ $(PF_6)^-$ were redissolved in CD_3CN. ^1H NMR spectra of DB24C8, [ruthenocenylmethyl(4-methylphenyl)ammonium·DB24C8]$^+$$(PF_6)^-$ and [ruthenocenylmethyl(4-methylphenyl) ammonium]$^+$$(PF_6)^-$ in CD_3CN are shown in Supplementary Figure S8. In the pseudorotaxane complex, the signals at 4.19 and 4.58 ppm are assigned to $-NCH_2-$ in the complex. The signals associated with the aromatic protons H^f and H^g of [ruthenocenylmethyl(4-methylphenyl)ammonium]$^+$$(PF_6)^-$ shift from 7.32 and 7.26 ppm to 7.17 and 7.15 ppm upon complexation. Peaks of protons of ruthenocenyl group (H^a, H^b, H^c) and peaks around ammonium cation (H^d, H^e) in the axle molecule also show upfield shift. The molar ratio of complex/uncomplex for ruthenocene-containing pseudorotaxane in CD_3CN solution is estimated to be 0.72, which is 1.7 times lower than ferrocene-containing pseudorotaxane (1.25). This comparison indicates that replacement of the metal centre from Fe to Ru reduces the association between ring and ammonium axle molecules.

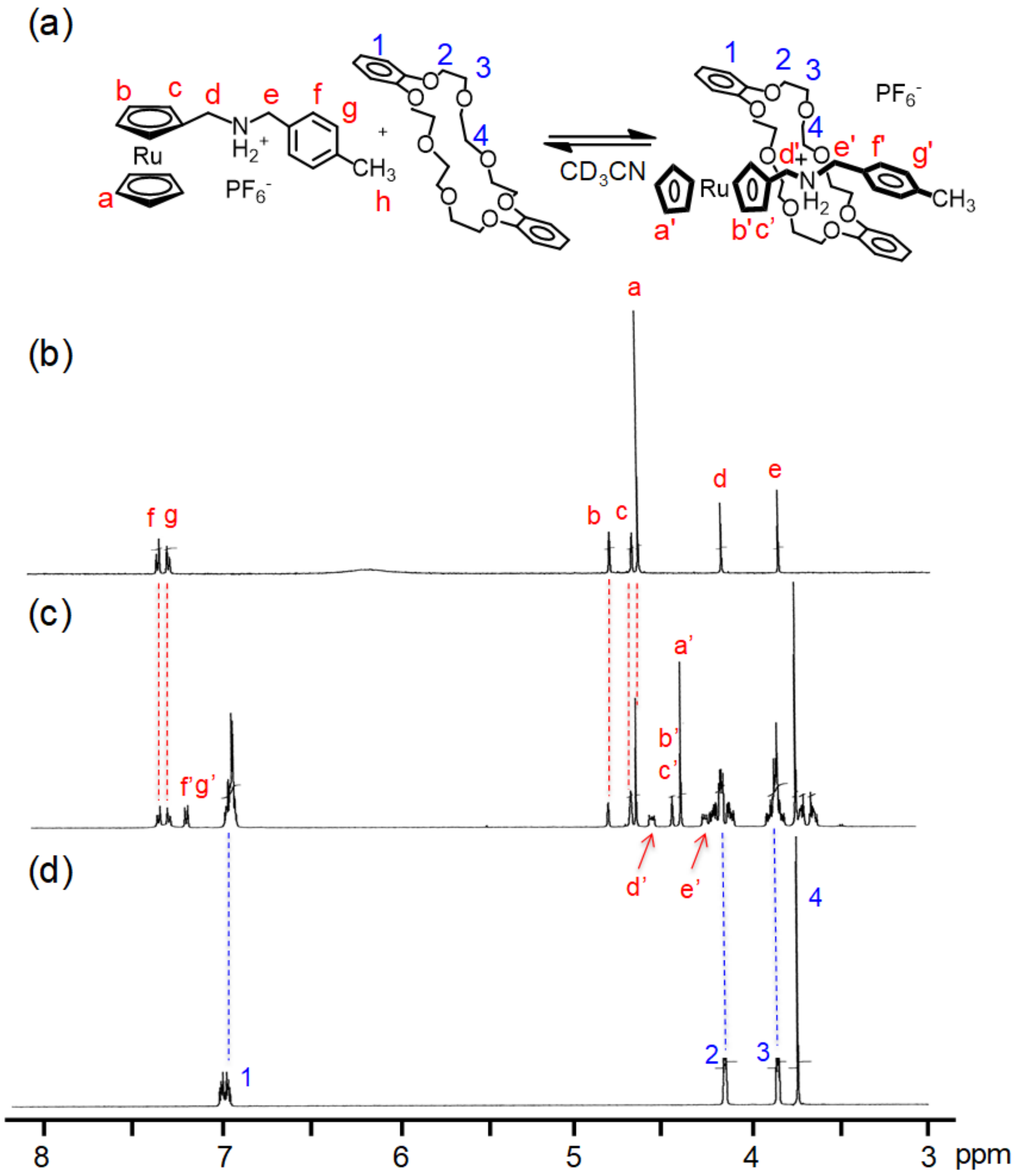

Supplementary Figure S8. ^{1}H NMR spectra (500 MHz, r.t, CD_3CN) of ruthenocene-containing pseudorotaxane and its components. (a) Dissociation/association of the pseudorotaxane in a solution. (b) ^{1}H NMR spectrum of [ruthenocenylmethyl(4-methylphenyl)-ammonium]$^+$(PF_6)$^-$, (c) [ruthenocenylmethyl(4-methylphenyl)ammonium·DB24C8]$^+$(PF_6)$^-$, (d) DB24C8.

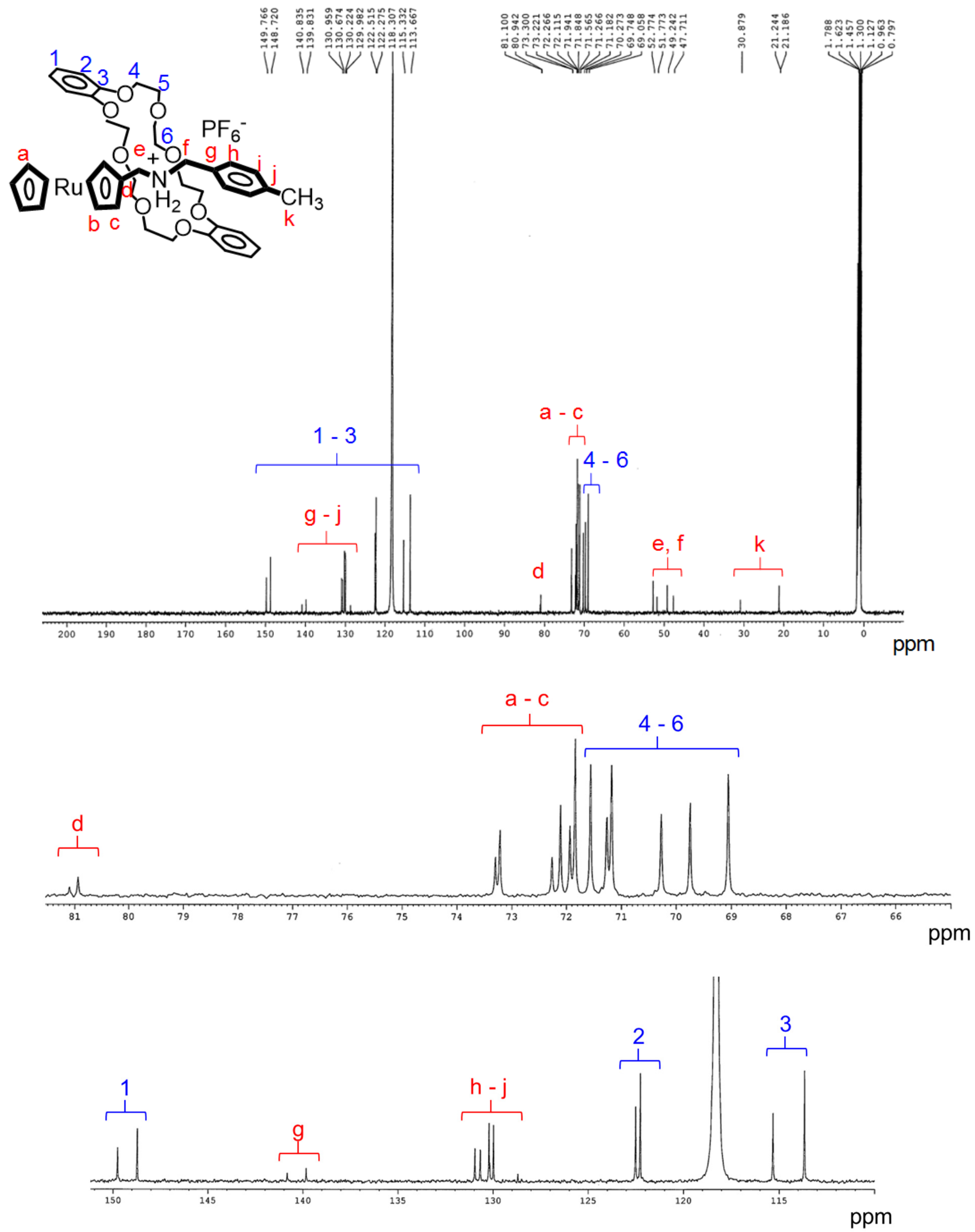

Supplementary Figure S9. ^{13}C NMR spectra (500 MHz, CD_3CN) of [ruthenocenylmethyl(4-methylphenyl) ammonium·DB24C8]$^+$$(PF_6)^-$. Signals involve the pseudorotaxane and uncomplex components in CD_3CN solution.

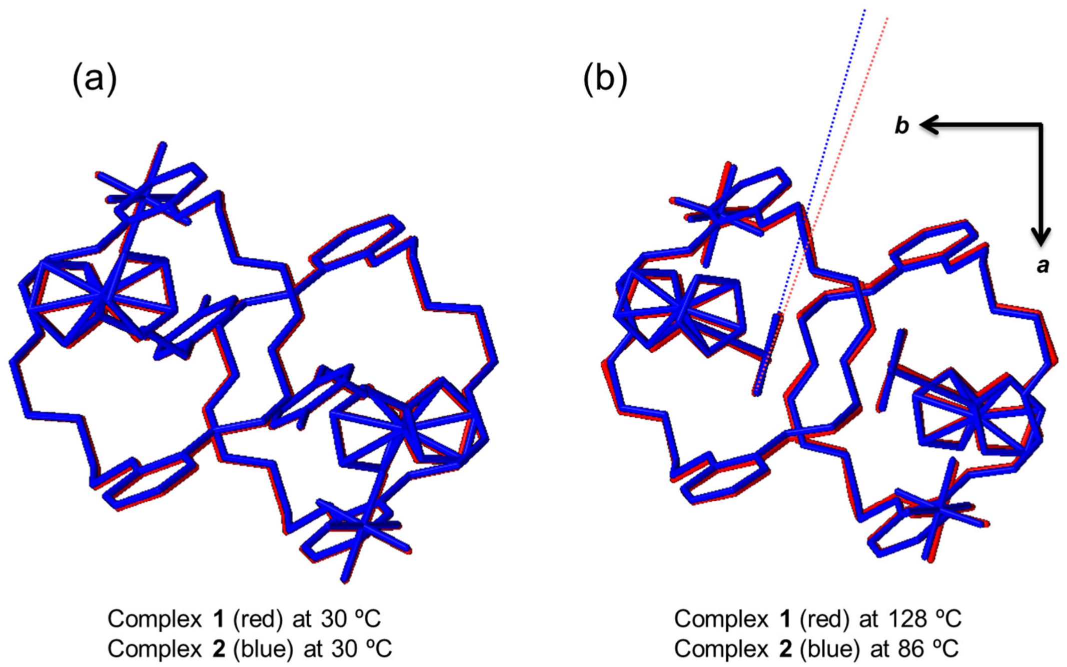

Supplementary Figure S10. Overlay drawings of molecular structures. A pair of packing structure of complex **1** and **2** obtained from single crystal X-ray crystallography (a) at 30 ºC and (b)**,** at high temperature phase.

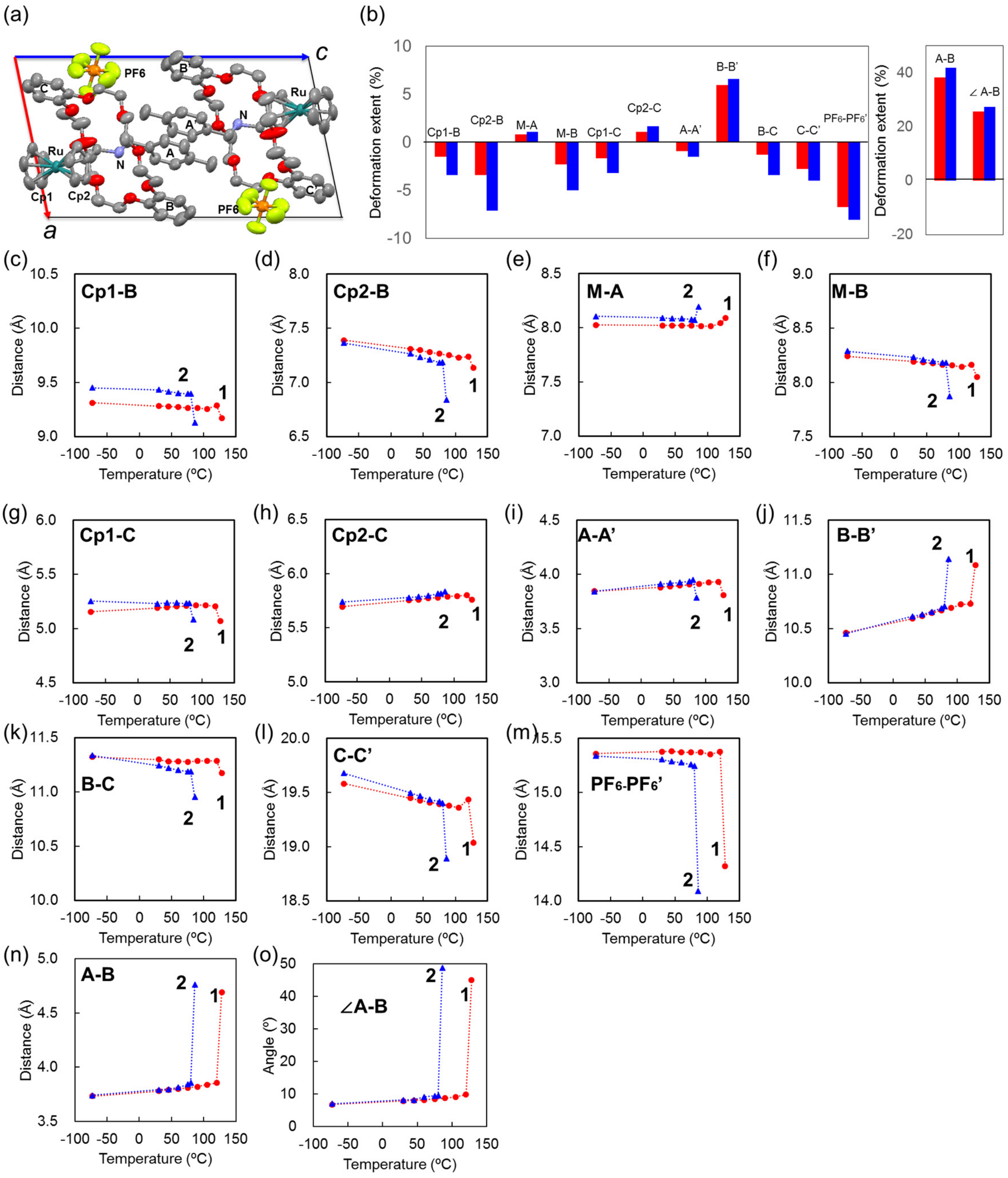

Supplementary Figure S11. Single-crystal X-ray crystallographic results of complexes **1** and **2** at various temperatures. (a) ORTEP representation of a pair of complex **2** with 30 % probability. (b) bar chart for deformation extent. (c)-(o) specific distances and angles of complex **1** (red) and **2** (blue).

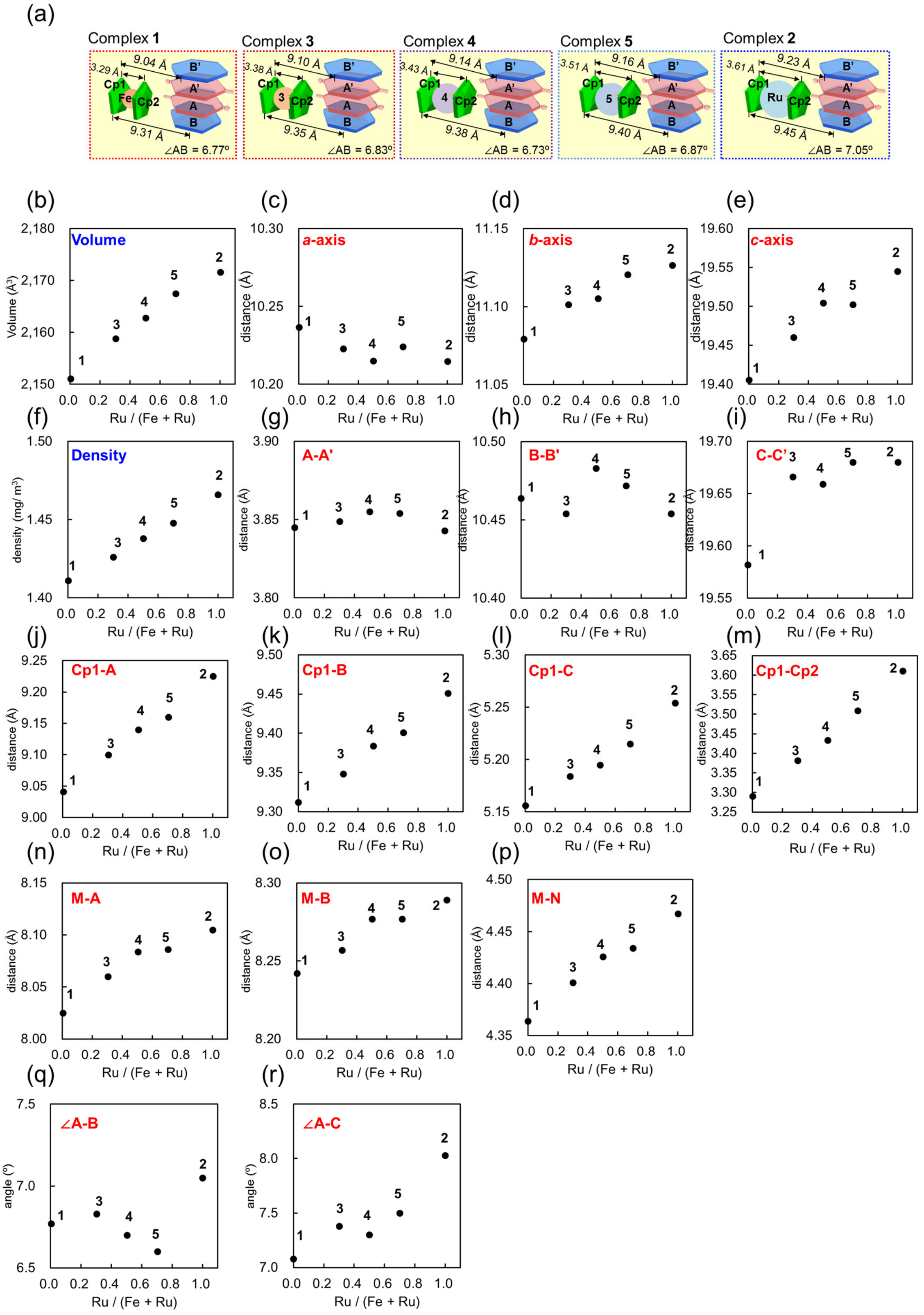

Supplementary Figure S12. Single-crystal X-ray crystallographic results of low temperature structures of complexes **1-5** at -73 ºC. (a) Illustration of mixed crystals with specific distances and angles between aromatic rings. (b)-(r) The unit cell volume, lattice parameters and intra/intermolecular distances and angles.

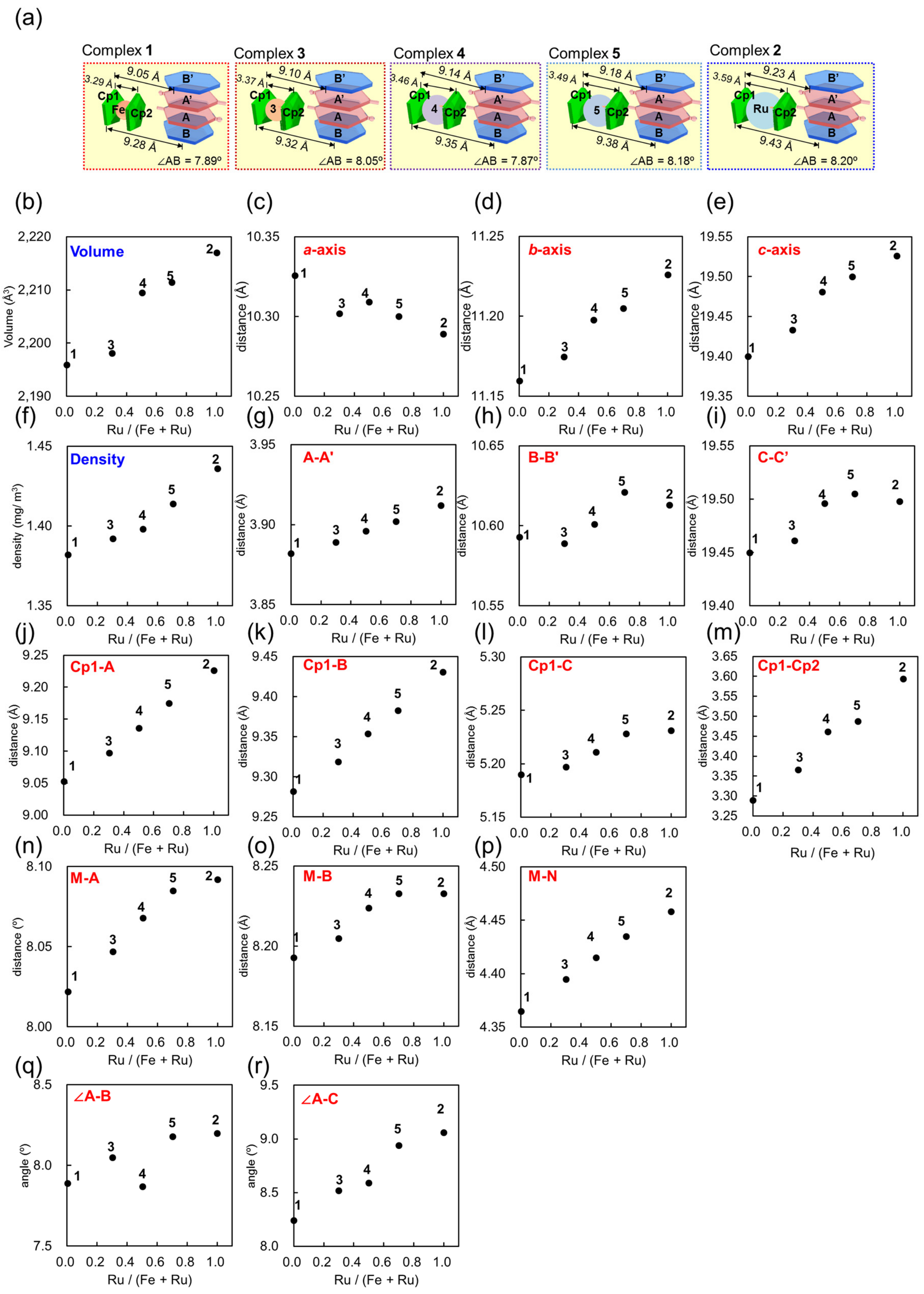

Supplementary Figure S13. Single-crystal X-ray crystallographic results of low temperature structures of complexes **1-5** at 30 ºC. (a) Illustration of mixed crystals with specific distances and angles between aromatic rings. (b)-(r) The unit cell volume, lattice parameters and intra/intermolecular distances and angles.

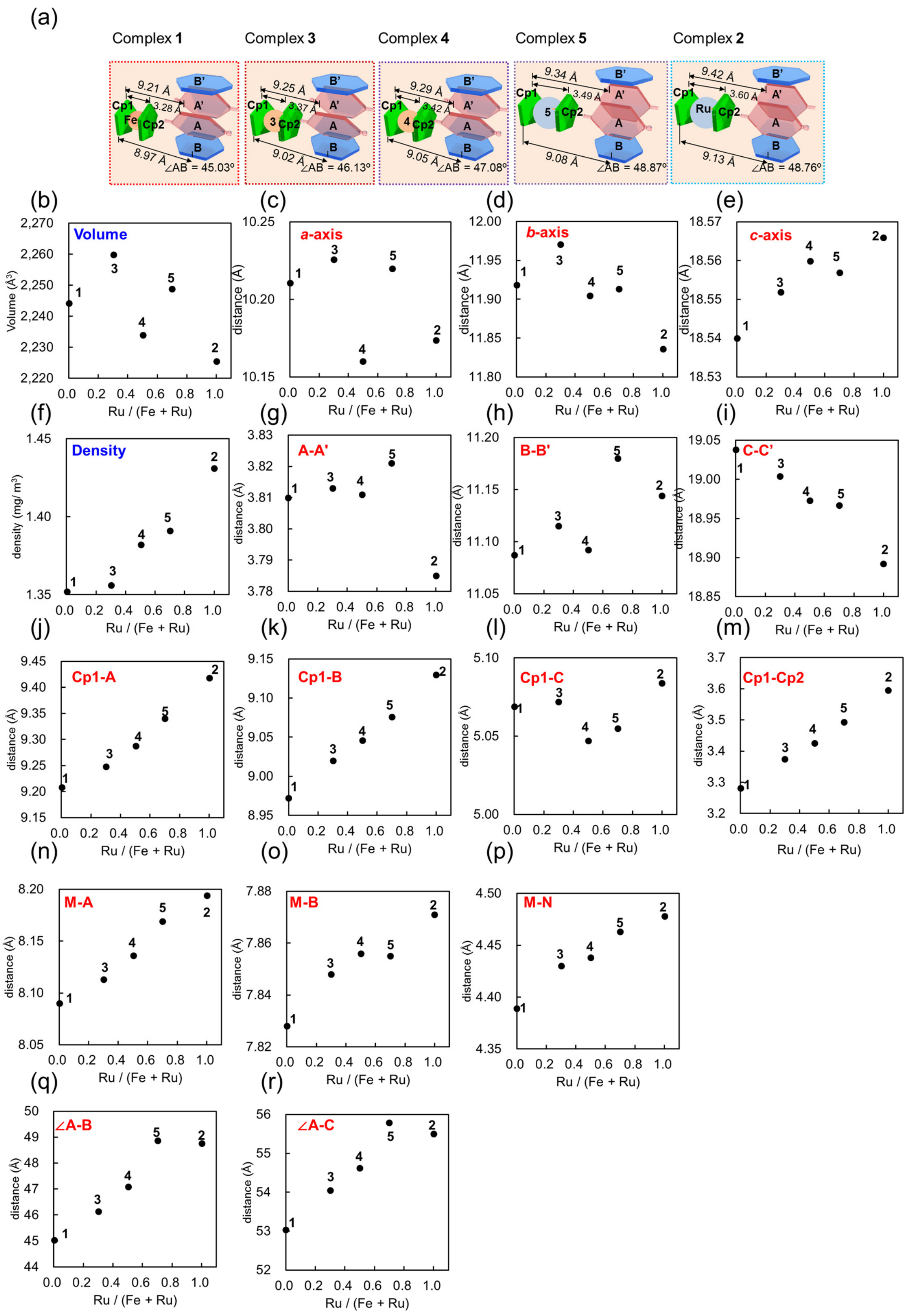

Supplementary Figure S14. Single-crystal X-ray crystallographic results of high temperature structures of complexes **1**-**5**. (a) Illustration of mixed crystals with specific distances and angles between aromatic rings. (b)-(r) The unit cell volume, lattice parameters and intra/intermolecular distances and angles.

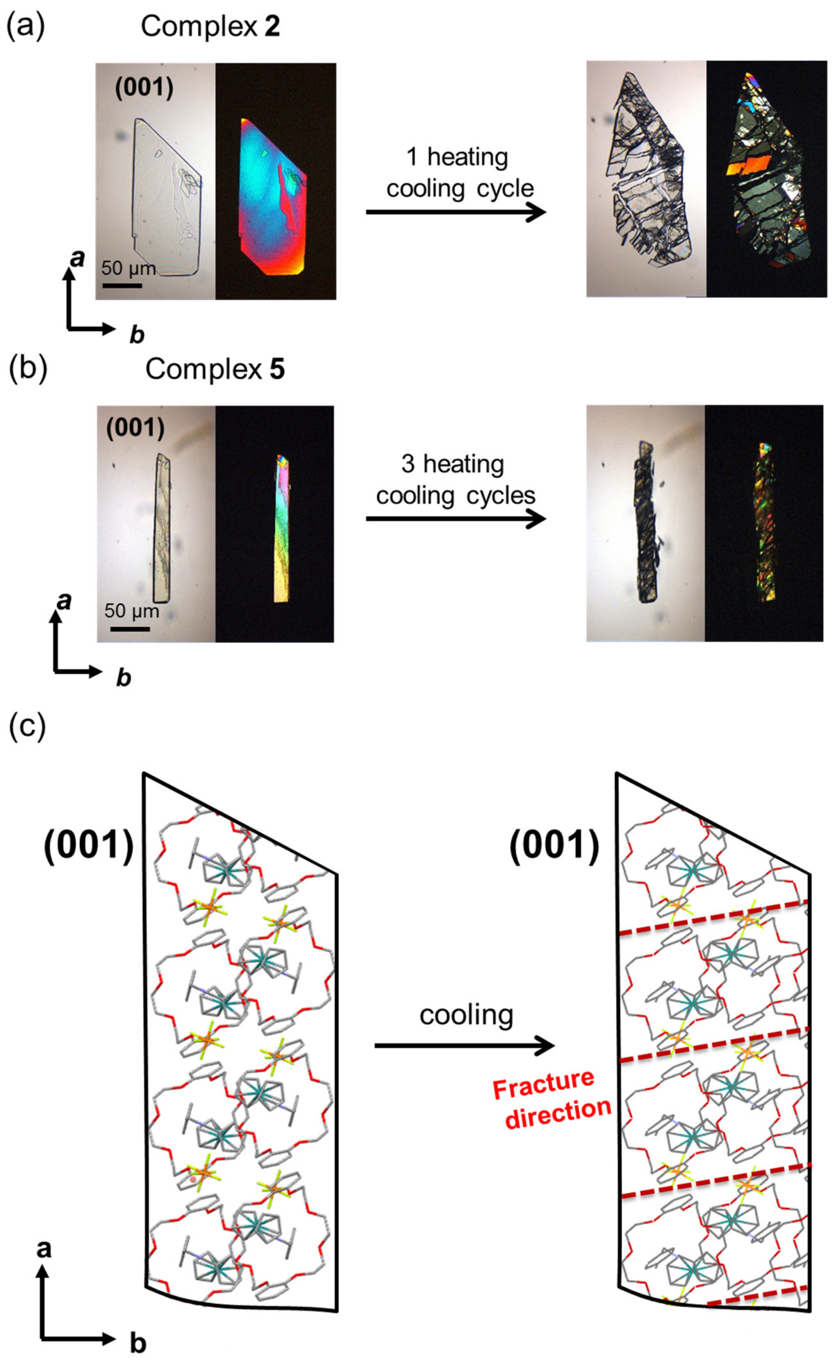

Supplementary Figure S15. Degradation of crystals of complexes **2** and **5**. (a) Optical micrographs of a single crystal of complex **2** before and after phase transition. See also the Supplementary Movie. (b) Optical micrographs of a single crystal of complex **5** before and after phase transition. (c) Packing structures of high and low temperature structures of complex **2**. Fracture direction is illustrated.

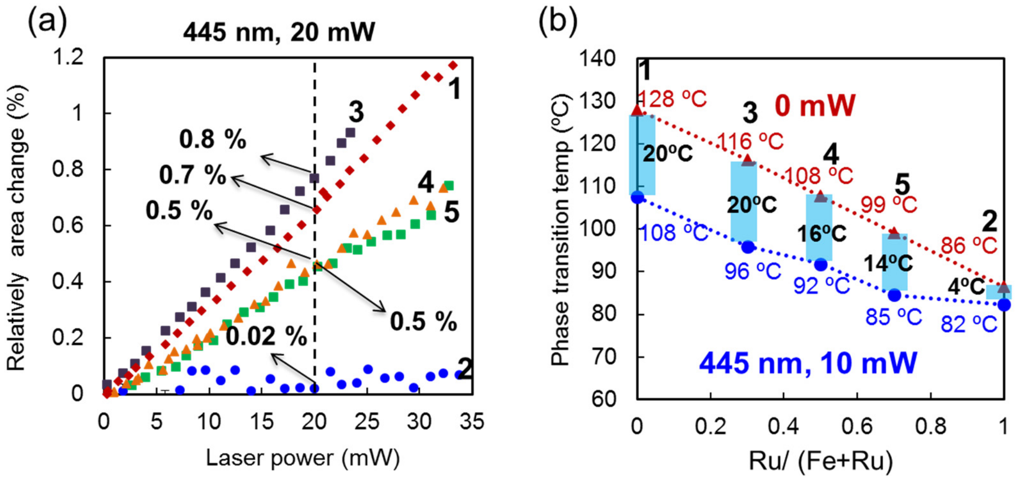

Supplementary Figure S16. Photo-responsive deformation. (a) Laser power dependence of the change in the relative area of the (001) facet of the single crystal induced by focused 445 nm laser irradiation. (b) The reduction of phase transition temperature induced by 445 nm laser irradiation.

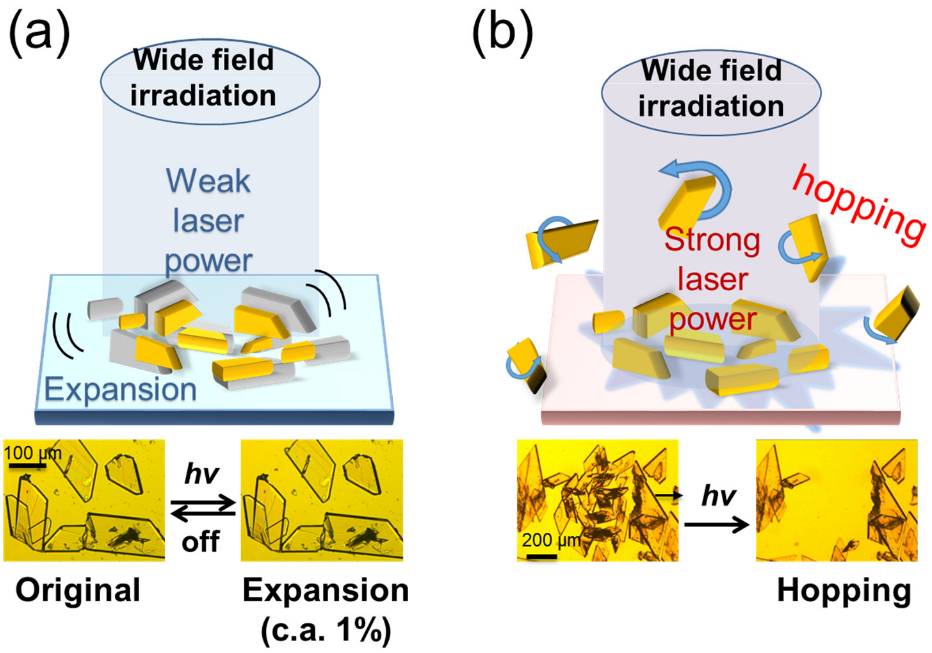

Supplementary Figure S17. Photo-triggered salient effects of complex **1**. (a) Reversible expansion and contraction of crystals of complex **1** are controlled by turning wide field laser irradiation (445 nm, 60 mW) on and off at 30 ºC. (b) Hopping of crystals of complex **1** is triggered by wide field irradiation (445 nm, 70 mW) at 30 ºC. See also the Supplementary Movie.

.

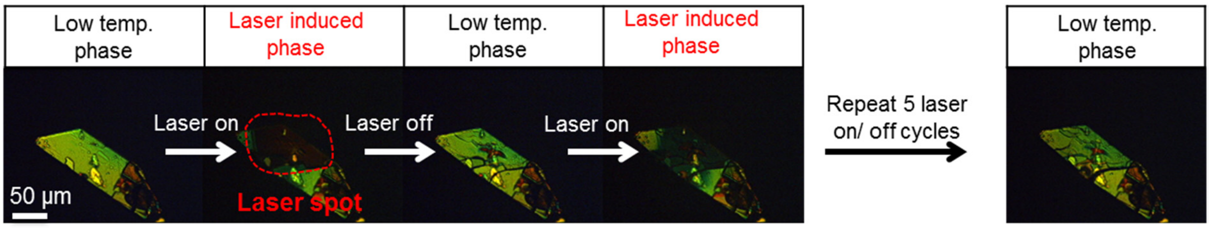

Supplementary Figure S18. Photo-triggered crystal-to-crystal phase transition of complex **3.** Thermal phase transition is controlled by focused laser (405 nm, 25 mW) at 30 ºC.

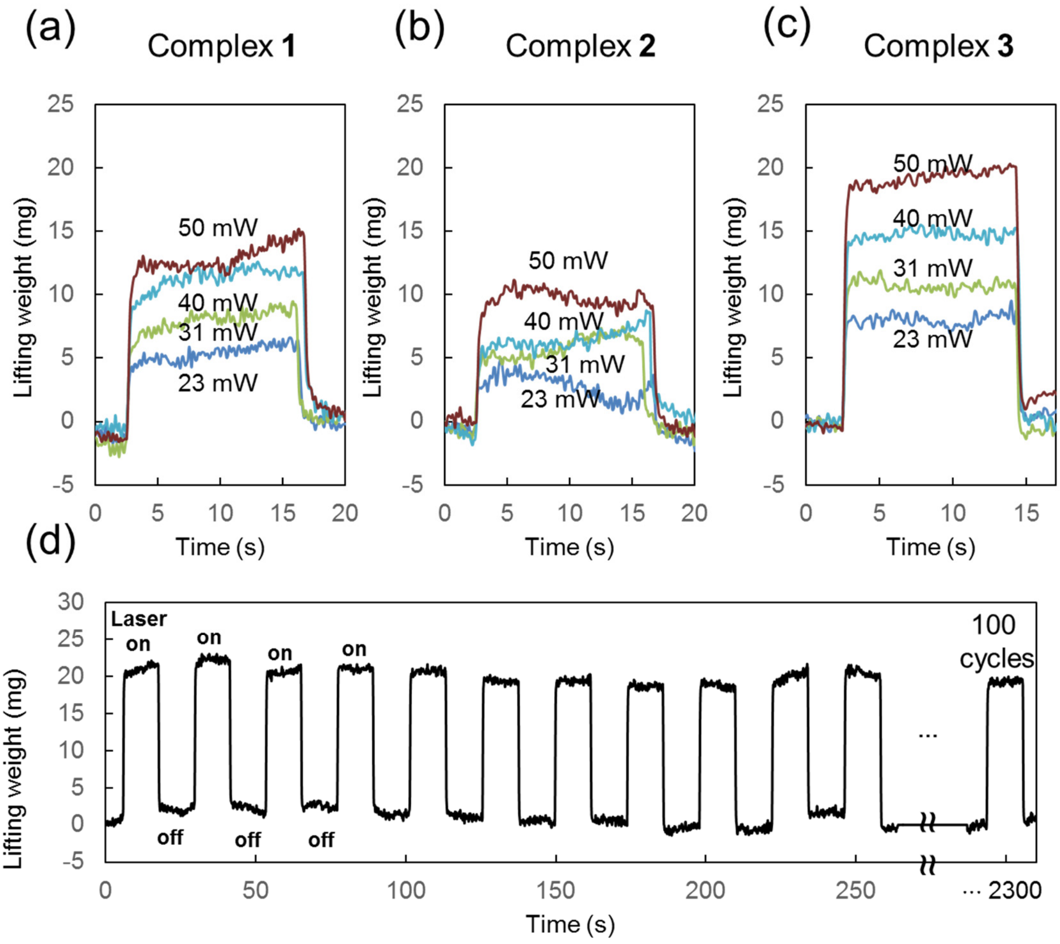

Supplementary Figure S19. Time dependence of force detection. Size expansion and contraction of the pseudorotaxane crystals are controlled by turning the 405-nm laser (50 mW) on and off at 30 ºC. (a) Time dependence of force detection for a crystal of complex **1** with a maximum lifting weight of 15 mg. (b) Complex **2** with a maximum lifting weight of 10 mg. (c) Complex **3** with a maximum lifting weight of 20 mg. These measurements were carried out under the same condition. (d) Repeating experiment of force detection for a crystal of complex **3**.

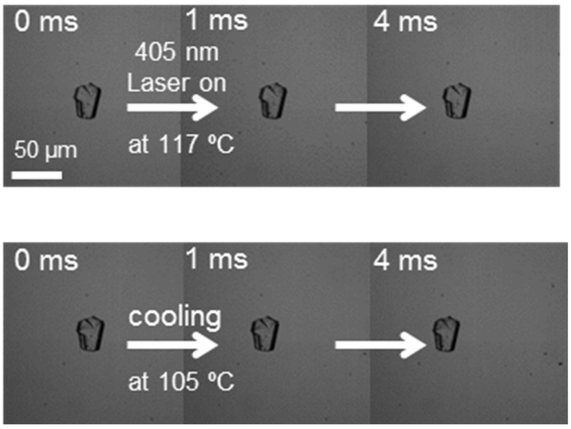

Supplementary Figure S20. Optical micrographs of a single crystal of complex **3** upon phase transition. These images were observed using a high-speed camera (1000 fps). See also the Supplementary Movie.

Supplementary Table S1. Crystal data and details of structure refinement of complex **2** at various temperatures.

Formula: $C_{43}H_{54}F_6RuNO_8P$

Molecular weight: 958.91

Crystal system: Triclinic

Space group: P1bar (No.2)

**Temp.**	**-73 °C**	**30 °C**	**45 °C**	**60 °C**	**75 °C**	**80 °C**	**84 °C**
a/ Å	10.2148(9)	10.2892(14)	10.2906(7)	10.2997(11)	10.3090(15)	10.311(2)	10.1738(18)
b/ Å	11.1265(8)	11.2261(15)	11.2476(8)	11.2779(12)	11.3223(17)	11.346(3)	11.836(2)
c/ Å	19.5452(12)	19.526(3)	19.5049(13)	19.492(2)	19.469(3)	19.453(5)	18.566(3)
α/deg	87.620(4)	87.659(3)	87.6689(15)	87.678(2)	87.658(3)	87.655(5)	86.473(3)
β/deg	78.083(4)	79.694(3)	80.0677(14)	80.431(2)	80.869(3)	81.034(5)	86.063(3)
γ/deg	89.044(5)	88.922(3)	88.8640(14)	88.815(2)	88.754(3)	88.711(5)	88.370(3)
V/ $Å^3$	2171.6(3)	2217.0(5)	2221.7(3)	2230.6(4)	2241.5(6)	2245.7(9)	2225.5(7)
Z	2	2	2	2	2	2	2
μ(MoKα) /cm^{-1}	4.76	4.66	4.65	4.63	4.61	4.60	4.64
F(000)	992	992	992	992	992	992	992
D/g cm^{-3}	1.436	1.436	1.433	1.428	1.421	1.418	1.431
crystal size /mm	0.59 x 0.10 x 0.03	0.25 x 0.20 x 0.10	0.25 x 0.20 x 0.10	0.25 x 0.20 x 0.10	0.25 x 0.20 x 0.10	0.25 x 0.20 x 0.10	0.25 x 0.20 x 0.10
Unique reflections	18976	36555	36737	36874	36761	36479	33649
Used reflections [I>2.0σ (I)]	7574	9173	9208	9255	9281	9298	9127
R	0.0505	0.0581	0.0597	0.0607	0.0622	0.0620	0.0660
*R*w	0.1117	0.1464	0.1476	0.1496	0.1484	0.1613	0.1591
GOF	1.082	1.003	0.983	0.985	0.977	0.931	0.957

Supplementary Table S2. Crystal data of low temperature structures of complexes **1** to **5** at -73 ºC.

**Temperature: -73 ºC**					
	Complex **1**	Complex **3**	Complex **4**	Complex **5**	Complex **2**
Formula in synthesis	$C_{43}H_{54}F_6Fe$ NO_8P	$C_{43}H_{54}F_6$ $Ru_{0.3}Fe_{0.7}$ NO_8P	$C_{43}H_{54}F_6$ $Ru_{0.5}Fe_{0.5}$ NO_8P	$C_{43}H_{54}F_6$ $Ru_{0.7}Fe_{0.3}$ NO_8P	$C_{43}H_{54}F_6Ru$ NO_8P
Calcd. formula from X-ray crystallography	$C_{43}H_{54}F_6Fe$ NO_8P	$C_{43}H_{54}F_6$ $Ru_{0.20}Fe_{0.80}$ NO_8P	$C_{43}H_{54}F_6$ $Ru_{0.35}Fe_{0.65}$ NO_8P	$C_{43}H_{54}F_6$ $Ru_{0.58}Fe_{042}$ NO_8P	$C_{43}H_{54}F_6Ru$ NO_8P
molecular weight	913.69	927.26	936.3	945.35	958.91
crystal system	Triclinic	Triclinic	Triclinic	Triclinic	Triclinic
space group	P1bar (No.2)	P1bar (No.2)	P1bar (No.2)	P1bar (No.2)	P1bar (No.2)
a/ Å	10.2368(6)	10.2230(4)	10.2150(7)	10.2242(5)	10.2148(9)
b/ Å	11.0792(6)	11.1013(6)	11.1053(7)	11.1205(5)	11.1265(8)
c/ Å	19.4057(10)	19.4604(10)	19.5046(13)	19.5026(11)	19.5452(12)
α/deg	87.149(13)	87.237(3)	87.272(2)	87.4130(10)	87.620(4)
β/deg	78.123(12)	78.141(2)	78.132(2)	78.1010(10)	78.083(4)
γ/deg	89.039(12)	89.001(2)	89.057(2)	89.0010(10)	89.044(5)
V/ Å^3	2151.1	2158.82(18)	2162.8(2)	2167.48(19)	2171.6(3)
Z	2	2	2	2	2
μ (MoKα) /cm^{-1}	4.66	4.69	4.71	4.74	4.76
F(000)	956	956	974	981	992
D/g cm^{-3}	1.411	1.426	1.438	1.448	1.466
crystal size /mm	0.55 x 0.39 x 0.02	0.69 x 0.19 x 0.19	0.74 x 0.55 x 0.50	0.95 x 0.91 x 0.38	0.59 x 0.10 x 0.03
Unique reflections	73602	17749	19128	19703	18976
Used reflections [I>2.0σ (I)]	7588	7557	7602	7633	7574
R	0.0383	0.0518	0.0424	0.0451	0.0505
*R*w	0.0979	0.1329	0.1243	0.1311	0.1117
GOF	1.036	1.102	1.202	1.140	1.082

Supplementary Table S3. Crystal data of low temperature structures of complexes **1** to **5** at 30 ºC.

**Temperature: 30 ºC**					
	Complex **1**	Complex **3**	Complex **4**	Complex **5**	Complex **2**
Formula in synthesis	$C_{43}H_{54}F_6Fe$ NO_8P	$C_{43}H_{54}F_6$ $Ru_{0.3}Fe_{0.7}$ NO_8P	$C_{43}H_{54}F_6$ $Ru_{0.5}Fe_{0.5}$ NO_8P	$C_{43}H_{54}F_6$ $Ru_{0.7}Fe_{0.3}$ NO_8P	$C_{43}H_{54}F_6Ru$ NO_8P
Calcd. formula from X-ray crystallography	$C_{43}H_{54}F_6Fe$ NO_8P	$C_{43}H_{54}F_6$ $Ru_{0.22}Fe_{0.78}$ NO_8P	$C_{43}H_{54}F_6$ $Ru_{0.36}Fe_{0.64}$ NO_8P	$C_{43}H_{54}F_6$ $Ru_{0.62}Fe_{038}$ NO_8P	$C_{43}H_{54}F_6Ru$ NO_8P
molecular weight	913.69	921.62	930.19	941.73	958.91
crystal system	Triclinic	Triclinic	Triclinic	Triclinic	Triclinic
space group	P1bar (No.2)	P1bar (No.2)	P1bar (No.2)	P1bar (No.2)	P1bar (No.2)
a/ Å	10.3258(4)	10.3020(5)	10.3092(4)	10.300(3)	10.2892(14)
b/ Å	11.1596(4)	11.1747(6)	11.1978(5)	11.205(3)	11.2261(15)
c/ Å	19.3998(8)	19.4330(10)	19.4810(8)	19.500(4)	19.526(3)
α/deg	87.2796(10)	87.3910(10)	87.4720(10)	87.514(5)	87.659(3)
β/deg	79.5728(9)	79.6190(10)	79.5820(10)	79.592(5)	79.694(3)
γ/deg	88.8537(10)	88.8740(10)	88.8430(10)	88.968(5)	88.922(3)
V/ $Å^3$	2195.93	2198.1(2)	2209.50(16)	2211.4(9)	2217.0(5)
Z	2	2	2	2	2
μ (MoKα) /cm^{-1}	4.57	4.59	4.59	4.62	4.66
F(000)	956	960	969	978	992
D/g cm^{-3}	1.382	1.392	1.398	1.414	1.436
crystal size /mm	0.30 x 0.22 x 0.10	0.23 x 0.20 x 0.15	0.24 x 0.22 x 0.12	0.23 x 0.20 x 0.18	0.25 x 0.20 x 0.10
Unique reflections	33063	34156	34713	29231	36555
Used reflections [I>2.0σ (I)]	9057	9067	9089	10030	9173
R	0.0517	0.0514	0.0476	0.0546	0.0581
*R*w	0.1334	0.1357	0.1289	0.1396	0.1464
GOF	0.939	1.029	1.026	0.996	1.003

Supplementary Table S4. Crystal data of high temperature structures of complexes **1** to **5**.

	Complex **1**	Complex **3**	Complex **4**	Complex **5**	Complex **2**
**Temp.**	**128 ºC**	**120 ºC**	**111 ºC**	**102 ºC**	**84 ºC**
Formula in synthesis	$C_{43}H_{54}F_6$ $FeNO_8P$	$C_{43}H_{54}F_6$ $Ru_{0.3}Fe_{0.7}$ NO_8P	$C_{43}H_{54}F_6$ $Ru_{0.5}Fe_{0.5}$ NO_8P	$C_{43}H_{54}F_6$ $Ru_{0.7}Fe_{0.3}$ NO_8P	$C_{43}H_{54}F_6$ $RuNO_8P$
Calcd. formula from X-ray crystallography	$C_{43}H_{54}F_6$ $FeNO_8P$	$C_{43}H_{54}F_6$ $Ru_{0.26}Fe_{0.73}$ NO_8P	$C_{43}H_{54}F_6$ $Ru_{0.4}Fe_{0.6}$ NO_8P	$C_{43}H_{54}F_6$ $Ru_{0.62}Fe_{038}$ NO_8P	$C_{43}H_{54}F_6$ $RuNO_8P$
molecular weight	913.69	922.87	929.76	941.73	958.91
crystal system	Triclinic	Triclinic	Triclinic	Triclinic	Triclinic
space group	P1bar (No.2)	P1bar (No.2)	P1bar (No.2)	P1bar (No.2)	P1bar (No.2)
a/ Å	10.211 (4)	10.226(9)	10.160(12)	10.220(5)	10.1738(18)
b/ Å	11.919(6)	11.971(11)	11.905(13)	11.914(6)	11.836(2)
c/ Å	18.540(7)	18.552(17)	18.560(2)	18.557(9)	18.566(3)
α/deg	85.670(3)	86.056(18)	86.180(2)	86.118(10)	86.473(3)
β/deg	86.500(3)	86.370(18)	86.110(3)	86.150(11)	86.063(3)
γ/deg	87.660(4)	87.864(19)	88.270(3)	88.582(12)	88.370(3)
V/ Å^3	2244.30	2260.0(4)	2234.0(4)	2248.9(19)	2225.5(7)
Z	2	2	2	2	2
μ (MoKα) /cm^{-1}	4.69	4.44	4.54	4.54	4.64
F(000)	956	961	966	978	992
D/g cm^{-3}	1.352	1.356	1.382	1.391	1.431
crystal size /mm	0.20 x 0.20 x 0.08	0.25 x 0.20 x 0.16	0.30 x 0.25 x 0.13	0.24 x 0.20 x 0.17	0.25 x 0.20 x 0.10
Unique reflections	11708	28590	28193	27291	33649
Used reflections [I>2.0σ (I)]	6842	9077	8932	8815	9127
R	0.1097	0.0767	0.0683	0.0637	0.0660
*R*w	0.2798	0.1939	0.1802	0.1705	0.1591
GOF	1.042	0.966	0.966	1.015	0.957

Supplementary Table S5. Intra-/intermolecular distances and angles of low temperature structures of complexes **1** to **5** at -73 ºC.

	Complex **1**	Complex **3**	Complex **4**	Complex **5**	Complex **2**
Fe:Ru	10:0	7:3	5:5	3:7	0:10
**Distance (Å)**					
Ring A-Ring B	3.733	3.730	3.733	3.741	3.740
Ring A-Ring A’	3.845	3.849	3.855	3.854	3.843
Ring B-Ring B’	10.464	10.454	10.483	10.472	10.454
Ring A-Ring C	9.274	9.300	9.303	9.300	9.310
Ring B-Ring C	11.324	11.331	11.332	11.334	11.337
Ring C-Ring C’	19.582	19.666	19.659	19.680	19.680
PF_6-PF_6’	15.361	15.352	15.352	15.343	15.338
N-Ring A	3.726	3.729	3.730	3.727	3.715
N-Ring B	5.071	5.063	5.066	5.069	5.056
N-Ring C	6.284	6.302	6.300	6.301	6.319
N-M	4.364	4.401	4.426	4.434	4.467
Cp1- Ring A	9.041	9.100	9.140	9.160	9.225
Cp1- Ring B	9.312	9.348	9.384	9.401	9.451
Cp1- Ring C	5.156	5.184	5.195	5.215	5.254
Cp2- Ring A	7.207	7.217	7.223	7.209	7.211
Cp2- Ring B	7.389	7.384	7.388	7.375	7.363
Cp2- Ring C	5.695	5.707	5.704	5.714	5.739
Cp1-Cp2	3.290	3.382	3.433	3.509	3.611
M-Ring A	8.025	8.060	8.084	8.086	8.105
M-Ring B	8.242	8.257	8.277	8.277	8.289
M-Ring C	5.213	5.219	5.217	5.221	5.222
**Angle (º)**					
A & (001)planes	71.59	71.71	71.68	71.62	71.86
B & (001) planes	65.12	65.14	65.31	65.35	65.23
C& (001) planes	66.72	66.72	66.94	66.80	66.63
A & C planes	7.08	7.38	7.30	7.50	8.03
A & B planes	6.77	6.83	6.70	6.60	7.05
B & C planes	3.43	3.83	3.73	3.87	3.87

Supplementary Table S6. Intra-/intermolecular distances and angles of low temperature structures of complexes **1** to **5** at 30 ºC.

	Complex **1**	Complex **3**	Complex **4**	Complex **5**	Complex **2**
Fe:Ru	10:0	7:3	5:5	3:7	0:10
**Distance (Å)**					
Ring A-Ring B	3.780	3.779	3.786	3.793	3.791
Ring A-Ring A’	3.882	3.889	3.896	3.902	3.912
Ring B-Ring B’	10.593	10.589	10.601	10.621	10.613
Ring A-Ring C	9.230	9.196	9.209	9.211	9.193
Ring B-Ring C	11.299	11.255	11.268	11.268	11.244
Ring C-Ring C’	19.450	19.461	19.496	19.505	19.498
PF_6-PF_6’	15.379	15.348	15.356	15.344	15.306
N-Ring A	3.791	3.717	3.720	3.721	3.708
N-Ring B	5.056	5.047	5.055	5.059	5.042
N-Ring C	6.242	6.241	6.247	6.246	6.241
N-M	4.365	4.395	4.415	4.435	4.458
Cp1- Ring A	9.053	9.097	9.136	9.175	9.226
Cp1- Ring B	9.282	9.319	9.354	9.383	9.431
Cp1- Ring C	5.190	5.197	5.211	5.228	5.231
Cp2- Ring A	7.183	7.180	7.185	7.184	7.167
Cp2- Ring B	7.309	7.298	7.302	7.294	7.265
Cp2- Ring C	5.753	5.751	5.762	5.771	5.779
Cp1-Cp2	3.286	3.366	3.461	3.487	3.594
M-Ring A	8.022	8.047	8.068	8.085	8.092
M-Ring B	8.193	8.205	8.224	8.233	8.233
M-Ring C	5.258	5.256	5.260	5.262	5.251
**Angle (º)**					
A & (001)planes	72.73	72.85	72.96	73.10	73.26
B & (001) planes	65.34	65.37	65.61	65.57	65.74
C& (001) planes	67.06	67.15	67.07	67.18	67.07
A & C planes	8.24	8.52	8.59	8.94	9.06
A & B planes	7.89	8.05	7.87	8.18	8.20
B & C planes	3.60	3.70	3.64	3.77	3.52

Supplementary Table S7. Intra-/intermolecular distances and angles of high temperature structures of complexes **1** to **5**.

	Complex **1**	Complex **3**	Complex **4**	Complex **5**	Complex **2**
Fe:Ru	10:0	7:3	5:5	3:7	0:10
**Distance (Å)**					
Ring A-Ring B	4.692	4.702	4.705	4.765	4.761
Ring A-Ring A'	3.810	3.813	3.811	3.821	3.785
Ring B-Ring B'	11.087	11.115	11.092	11.180	11.144
Ring A-Ring C	8.775	8.775	8.782	8.780	8.785
Ring B-Ring C	10.961	10.971	10.955	10.964	10.954
Ring C-Ring C'	19.038	19.004	18.973	18.967	18.892
PF_6-PF_6'	14.324	14.287	14.214	14.188	14.092
N-Ring A	3.713	3.695	3.706	3.713	3.723
N-Ring B	5.061	5.054	5.029	5.023	5.007
N-Ring C	6.002	6.022	6.030	6.050	6.054
N-M	4.389	4.430	4.438	4.463	4.478
Cp1- Ring A	9.208	9.248	9.288	9.340	9.418
Cp1- Ring B	8.972	9.020	9.046	9.076	9.130
Cp1- Ring C	5.069	5.072	5.047	5.055	5.084
Cp2- Ring A	7.141	7.146	7.138	7.151	7.151
Cp2- Ring B	6.889	6.887	6.862	6.842	6.837
Cp2- Ring C	5.757	5.803	5.796	5.834	5.834
Cp1-Cp2	3.282	3.374	3.426	3.493	3.595
M-Ring A	8.090	8.113	8.136	8.169	8.194
M-Ring B	7.828	7.848	7.856	7.855	7.871
M-Ring C	5.198	5.216	5.191	5.218	5.204
**Angle (º)**					
A & (001)planes	87.81	87.61	87.80	87.24	87.35
B & (001) planes	68.69	69.59	69.83	69.98	70.09
C& (001) planes	70.60	70.48	70.44	70.07	69.75
A & C planes	53.04	54.05	54.62	55.79	55.50
A & B planes	45.03	46.13	47.08	48.87	48.76
B & C planes	9.65	8.90	8.30	7.42	7.07

Supplementary Table S8. Thermodynamic parameters of crystals accompanied by phase transition estimated from DSC.

Complex	Fe:Ru	Endothermic peaks				Exothermic peaks			
		Temp. (°C)	*ΔH* (kJ mol^{-1})	*ΔS* (J K^{-1} mol^{-1})	**x*	Temp. (°C)	*ΔH* (kJ mol^{-1})	*ΔS* (J K^{-1} mol^{-1})	**x*
**1**	10:0	128	7.7	19	9.8	115	8.1	20	11.1
**3**	7:3	118	6.1	16	6.9	106	6.9	18	8.7
**4**	5:5	108	5.9	16	6.9	92	6.5	17	7.7
**5**	3:7	99	6.3	17	7.7	78	6.7	18	8.7
**2**	0:10	86	6.1	17	7.7	55	5.8	16	6.9

*The number of energetically equivalent microscopic states *x* were estimated from from Δ*S* values using Δ*S* = *R* ln(*x*). *R* is the gas constant.
