# Supplementary material for: Dynamic Pseudorotaxane Crystals Containing Metallocene Complexes
Source: Sci Rep. 2017 Oct 27;7:14195. doi: 10.1038/s41598-017-14505-7 (PMC5660174; doi:10.1038/s41598-017-14505-7)
Supplement: Supplementary file 1 — Supplement CIF files [file 41598_2017_14505_MOESM1_ESM.zip › 168049_1_additional_review_material_5173073_4wjhhr.pdf]

Submitted to *Scientific reports* (Original Article, Revised 1)

Dynamic Pseudorotaxane Crystals Containing Metallocene Complexes

Kai-Jen Chen,[1] Pei-Lin Chen[2] and Masaki Horie[1*]

[1]*Department of Chemical Engineering, National Tsing Hua University, 101, Sec. 2, Kuang-Fu Road, Hsinchu, 30013, Taiwan*

[2]*Instrumentation Center, National Tsing Hua University, 101, Sec. 2, Kuang-Fu Road, Hsinchu, 30013, Taiwan*

**To whom correspondence should be addressed (E-mail: mhorie@mx.nthu.edu.tw)*

Abstract

Molecular machines and switches composed of flexible pseudorotaxanes respond to external stimuli, transducing incident energy into mechanical motions. This study presents thermo- and photoresponsive dynamic pseudorotaxane crystals composed of axle molecules containing ferrocene or ruthenocene groups threaded through dibenzo[24]crown-8 ether rings. The ruthenocene-containing pseudorotaxane exhibits a crystal-to-crystal thermal phase transition at 86 °C, which is much lower than that of the ferrocene-containing pseudorotaxane (128 °C). Single-crystal X-ray crystallography at various temperatures reveals the details of the structural changes, and shows that the bulky ruthenocene provides distortion in the pseudorotaxane structure to facilitate

twisting of the axle molecule. A mixed ferrocene and ruthenocene pseudorotaxane crystal is applied to photomechanical conversion under 405 nm laser irradiation at 85 °C and provides a lifting force 6,400-times the weight of the crystal itself upon phase transition.

Introduction

Molecular machines and switches are of significant interest because they can be specifically designed to transduce chemical, electrical and photochemical energy into controllable mechanical motion[1-10]. In this context, rotaxanes, which comprise a molecular axle threaded through a ring molecule, undergo changes to the interactions between the axle and ring components under the influence of external stimuli, and operate as one-dimensional molecular switches[7-10]. To enhance the action of these molecular switches to the observable macroscopic level, structural control in the solid state is a key issue. For example, molecular muscles have been achieved using highly-oriented rotaxane films on substrates[11], and a rotaxane-based dry-type molecular muscle has been demonstrated as a photoresponsive actuator[12]. However, despite the rigid order of the solid state being advantageous for the integration and transmission of molecular motion, there are few reports in the literature on the structural control of rotaxanes in the crystal state[13-16].

Recently, stimuli-responsive dynamic molecular crystals have been gaining considerable research attention. The dynamics of these crystals include bending, twisting, rotation and salient effects such as hopping and flipping[17-26], allowing their potential application as actuators[23-32]. These dynamics are accompanied by molecular structural changes induced by thermal stimulus[17-19, 33, 34] or photochemical reactions such as photoaddition[35-37], photoisomerisation[22, 38, 39], or photocyclisation[40] in the crystal state. Some of these molecular structures and their stimuli-responsive structural changes have been precisely determined by single-crystal X-ray crystallography[18-21, 31, 33, 36-38] Such detailed structural characterisation is crucial for investigating the relationships between the molecular-level motion and the observable motion of crystals at the macroscopic scale.

Most of these dynamic crystals comprise a single molecule with reactive functional groups. However, in order to tailor molecular motions and provide multifunctionality, the use of interlocked molecular systems such as pseudorotaxanes is promising because they are composed of different functional molecules, i.e. a ring molecule with a flexible structure and host functionality, and a guest axle molecule that threads into the host. Adjusting and optimising the functionality of these components is a possible strategy for endowing pseudorotaxanes with controllable dynamics in the solid state.

Recently, we reported the thermal and photoinduced crystal-to-crystal phase

transitions of a pseudorotaxane composed of a ferrocenylmethyl(4-methylphenyl)ammonium axle and a dibenzo[24]crown-8 ether (DB24C8) ring (complex **1** in Fig. 1)[41-43]. This unique crystal behaviour was exploited for the remote control of microparticle transport and microswitching in an electric circuit[43]. During photomechanical energy conversion, the ferrocenyl group in the axle molecule of the pseudorotaxane acts as a photosensitiser. However, this ferrocene-containing pseudorotaxane is the only example reported to date of a pseudorotaxane capable of reversible switching accompanied by crystal-to-crystal phase transitions as directly observed by single-crystal X-ray crystallography[41-43]. Therefore, it is essential to develop further unique pseudorotaxanes with different functional groups that allow significant dynamic motion in the single-crystal state.

Consequently, herein we report thermo- and photoresponsive dynamic pseudorotaxane crystals containing a ruthenocene moiety (complex **2**) as well as mixed crystals with various ferrocene and ruthenocene ratios (complexes **3–5** in Fig. 1a). Ru has a larger atomic radius than Fe (132.5 and 124.1 pm, respectively). Therefore, it elongates the axle molecule to provide a highly-strained structure, which is expected to facilitate significant dynamic motion in the single-crystal state (Fig. 1b).

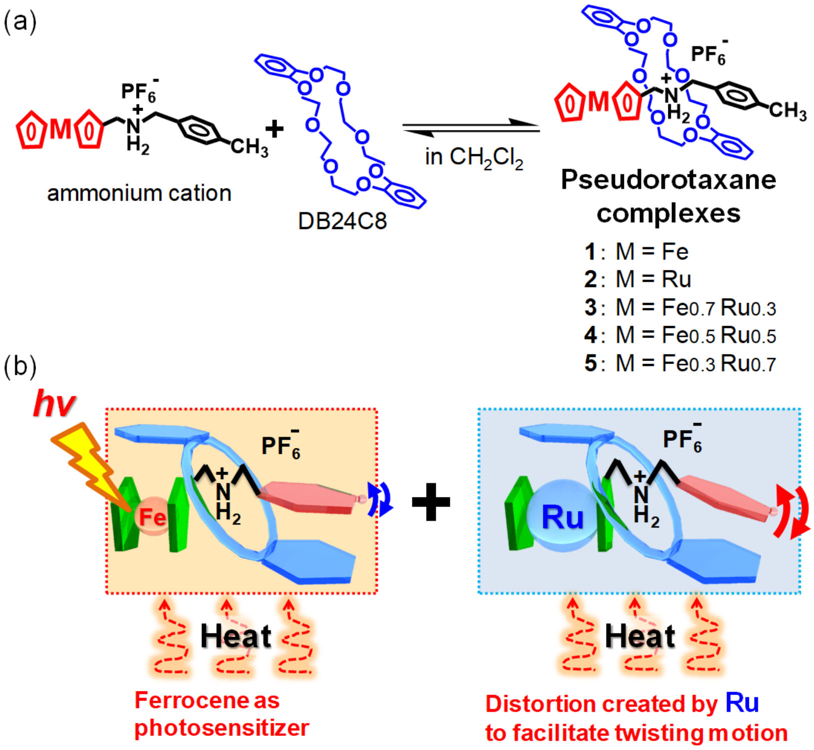

Figure 1. Pseudorotaxanes composed of Fe and Ru complexes. (**a**) Formation of pseudorotaxane molecules with different Fe and Ru ratios in dichloromethane solutions. (**b**) Illustration of stimuli-responsive dynamic pseudorotaxanes in the crystal state.

Results

Thermally induced phase transition

Complex **2** was synthesised by mixing the ruthenocene-containing axle molecule and DB24C8 in dichloromethane, followed by crystallisation by diffusion of diethyl ether vapour into the pseudorotaxane solution (Fig. 1, Supplementary Figs. S1-S9). Mixed crystals of complexes **3**–**5** containing Fe and Ru at different ratios (Fe:Ru = 7:3 for **3**, Fe:Ru = 5:5 for **4**, Fe:Ru = 3:7 for **5**) were also prepared by mixing complexes **1** and **2** at controlled molar ratios in dichloromethane solutions followed by crystallisation. Fe:Ru

ratios were estimated by single-crystal X-ray crystallography based on a substitutional disorder provided by occupation by different types of atoms at the same site. These values were consistent with the mixing ratios in the synthesis.

Fig. 2 compares the thermally induced crystal-to-crystal phase transition behaviours of single crystals of complexes **1**[41, 42] and **2**. Both crystals show dramatic interference colour change upon heating to 128 °C for complex **1** and 86 °C for complex **2**, accompanied by a crystal-to-crystal thermal phase transition (Fig. 2a). To investigate the structural changes, single-crystal X-ray crystallography was conducted for these complexes at different temperatures (Supplementary Tables S1-S7). Figs. 2b shows the temperature dependence of the unit cell volume V and the lengths of the a, b and c axes for crystals of complexes **1**[43] and **2** (Supplementary Table S1). Both crystals show expansion upon heating from −73 °C to just below their phase transition temperatures with the volumetric thermal expansion coefficients $\alpha_V = 263 \times 10^{-6}$ Å^3 K^{-1} for complex **1** and $\alpha_V = 223 \times 10^{-6}$ Å^3 K^{-1} for complex **2**. These values are some of the highest values for crystals of organic materials and metal-organic complexes reported to date[44-46]. Such significant thermal expansion is likely due to their flexible interlocked structures, which are expected to provide significant stimuli-responsive mechanical effects.

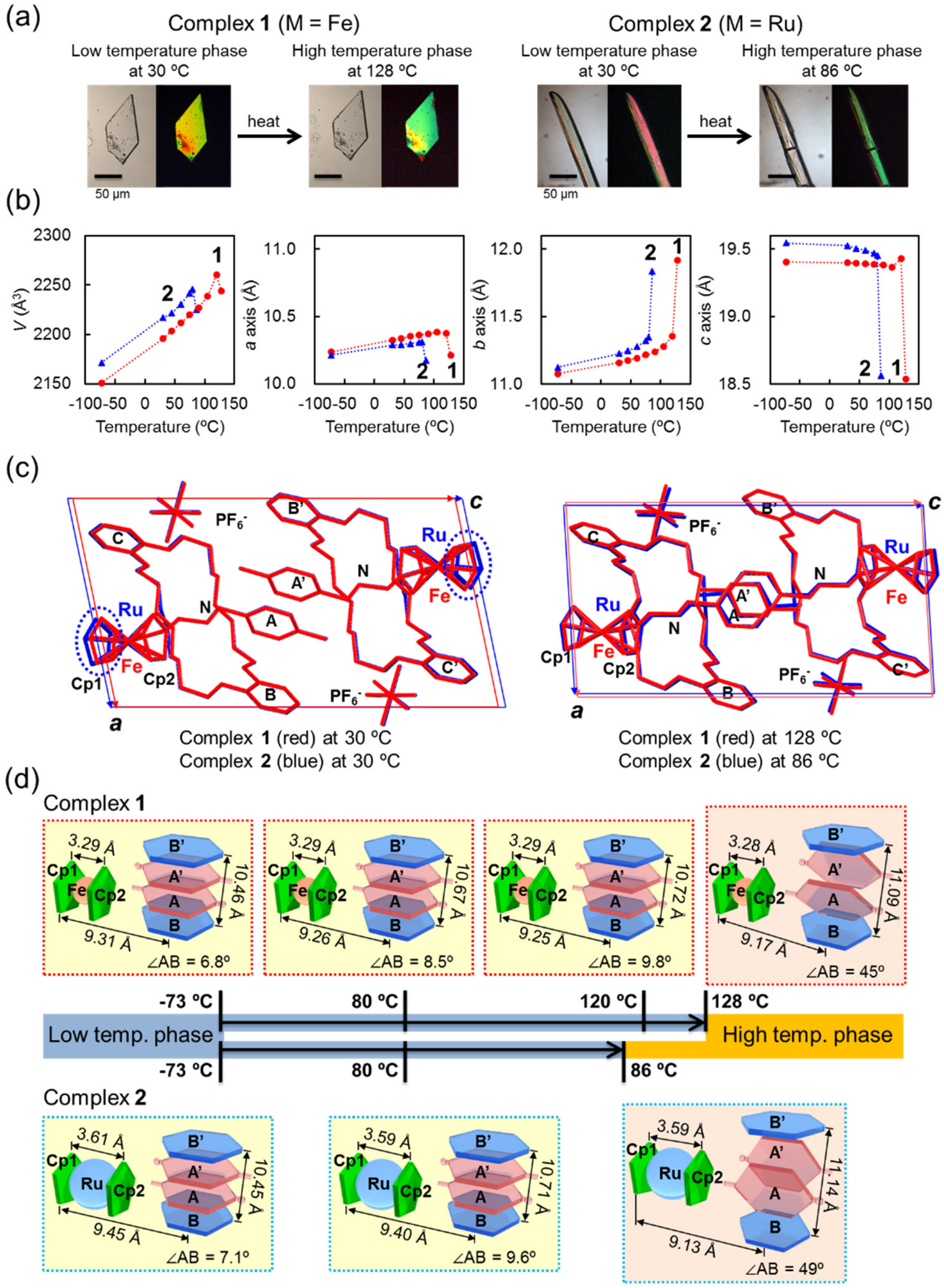

Figure 2. Thermal phase transition of pseudorotaxane crystals. (**a**) Optical micrographs of single crystals of complexes **1** and **2** without/with cross polarised light. (**b**) Volume and unit cell parameters of complexes **1** and **2** at different temperatures. (**c**) Overlays of the molecular structures of complexes **1** and **2** obtained by single-crystal X-ray crystallography at 30 °C, 128 °C (for complex **1**) and 86 °C (for complex **2**). See also the Supplementary Movie. (**d**) Schematic illustration of the molecular structural changes of complexes **1** and **2** on heating. Specific distances and angles between aromatic rings are depicted.

Upon heating beyond the phase transition temperatures, discontinuous changes in the unit cell parameters are observed (Figs. 2b). Complex **2** has similar *a* and *b* axis lengths to complex **1** both in the low and high temperature phases, whereas the *c* axis of complex **2** is significantly longer than that of complex **1** at −73 °C and becomes similar to that of complex **1** in the high temperature phase. The longer *c* axis for complex **2** in the low temperature phase is due to the presence of the bulky ruthenocene group.

Fig. 2c presents overlay drawings of the molecular structures in the unit cells for complexes **1** and **2** obtained by single-crystal X-ray crystallography, and the specific distances and angles are summarised in Fig. 2d and Supplementary Figs. S10 and S11. At low temperatures, both pseudorotaxane molecules are stabilised by an intramolecular π–π interaction between the tolyl group (aromatic ring A) of the axle molecule and the catechol group (aromatic ring B) of the cyclic molecule, and by an intermolecular π–π interaction between rings A and A' of two axle molecules. A significant difference is observed along the *c* axis due to the presence of different metallocene groups that span the *c* axis, where the distance between the two cyclopentadienyl (Cp) groups for complexes **1** and **2** are estimated to be 3.29 and 3.61 Å, respectively, at −73 °C. Therefore, in complex **2**, the bulky ruthenocene elongates the intramolecular distance along the *c* axis, i.e. the distance between Cp1 and ring B at −73 °C is 9.45 Å, which is longer than

that in complex **1** (Cp1-B = 9.31 Å) (Fig. 2d). Thus, the bulky ruthenocene distorts π–π interactions, effecting a slightly higher plane angle between rings A and B (7.1° at −73 °C and 9.6° at 80 °C) compared to that in complex **1** (6.8° at −73 °C and 8.5° at 80 °C). This distortion is one of the main reasons for the significantly lower phase transition temperature for complex **2**.

Upon heating, the plane angle between rings A and B for both complexes gradually increases, accompanied by an expansion of the crown ether cavity between rings B and B’ (Fig. 2d). These results indicate that the A-A’ pair of rings of the axle molecules are gradually activated on heating. Above the phase transition temperature, the A-A’ pair of rings eventually twist together maintaining their intermolecular π–π interaction. The plane angle between rings A and B for complex **2** significantly increases to 49°, which is higher than that of complex **1** (45°), whereas the Cp1-B distance for complex **2** (9.13 Å) becomes similar to that of complex **1** (9.17 Å) (Fig. 2d and Supplementary Fig. S10). These results suggest that the distortion along the *c* axis in the low temperature phase of complex **2** is released to provide a new distortion in the aromatic rings A, A’, B and B’ to form the more twisted axle pair A-A’ in the high temperature phase.

Thermal properties of mixed crystals

The large difference in the phase transition temperatures of complexes **1** and **2** is advantageous for tailoring the thermal properties of these pseudorotaxane complexes. Consequently, mixed crystals were prepared using various Fe and Ru ratios. Fig. 3a shows the differential scanning calorimetry (DSC) results for complexes **1–5**. All crystals show endo- and exothermic peaks upon heating and cooling, respectively, accompanied by crystal-to-crystal phase transitions. The thermodynamic parameters enthalpy (*ΔH*) and entropy (*ΔS*) were estimated from the peak areas and temperatures (Supplementary Table S8). As the fraction of Ru increases, these peaks shift to lower temperatures (Fig. 3b) with slightly lower *ΔH* and *ΔS* values.

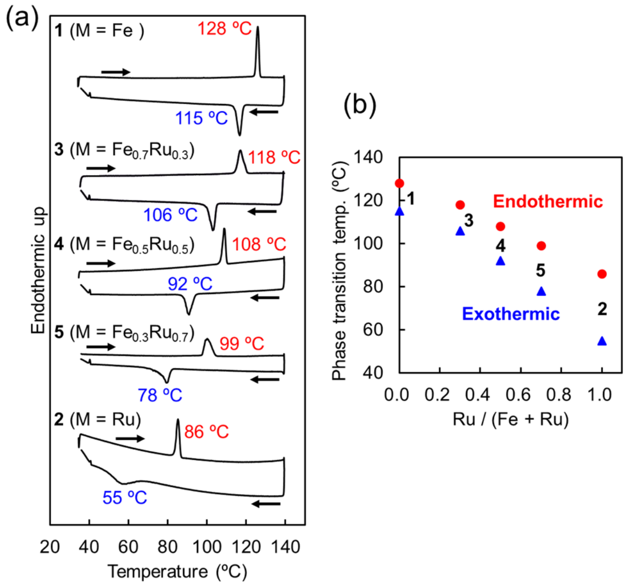

Figure 3. Thermal properties of mixed crystals. (**a**) DSC heating and cooling scans for complexes **1–5** at a scan rate of 5 °C min^{-1}. (**b**) Plots of crystal-to-crystal phase transition temperatures vs. molar fraction, i.e. Ru/(Fe + Ru).

This phenomenon can be explained by considering the molecular structures obtained by the single-crystal X-ray crystallography for complexes **1-5** (Supplementary Figs. S12-S14 and Supplementary Tables S2-S7). Intra- and intramolecular distances and angles are plotted with the molar fraction Ru/(Fe + Ru) at -73 °C, 30 °C and above the phase transition temperatures in Fig. 4. As the fraction of Ru increases, the distances Cp1-ring A and Cp1-ring B are elongated at all temperatures due to the presence of bulky Ru (Figs. 4a and 4b). Upon heating above the phase transition temperatures, significant increase in Cp1-ring A and decrease in Cp1-ring B are observed accompanied by the twisting of the pair of axle molecules. Similarly, as the fraction of Ru increases, the distance and angle between aromatic rings A and B are significantly increased above the phase transition temperatures (Figs. 4c and 4d). Consequently, the bulky ruthenocene provides distortion in the rotaxane structure to facilitate twisting of the axle molecules, resulting in the lower phase transition temperatures, as observed in DSC (Fig. 3).

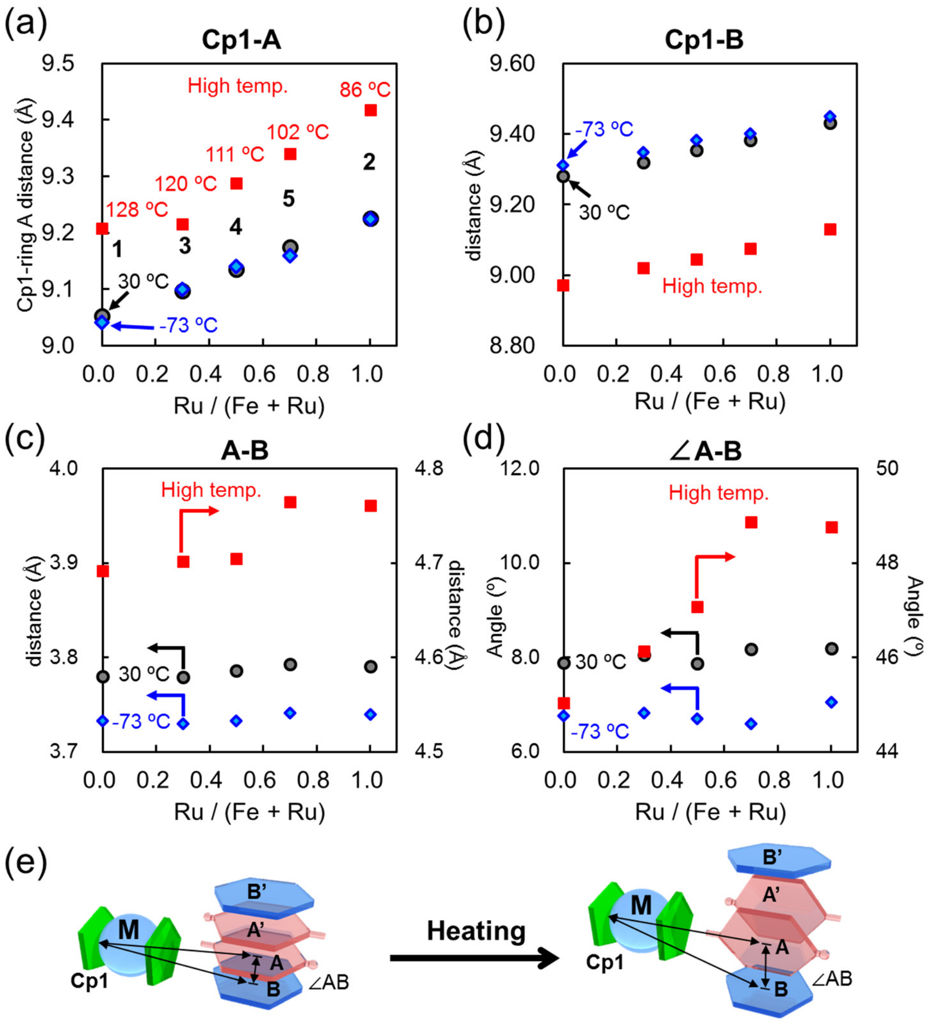

Figure 4. Plots of intra-/intermolecular distances and angles vs. Ru/(Fe + Ru). Distances between (**a**) Cp1 and aromatic ring A, (**b**) Cp1 and aromatic ring B, (**c**) aromatic rings A and B. (**d**) Angle between aromatic rings A and B. (**e**) Schematic illustration of the molecular structural changes of complexes **1-5** on heating.

To investigate macroscopic changes of the crystals accompanied by the phase transition, reflection micrographic images for crystals of complexes **1–5** were observed using a confocal laser microscope at 30 °C and above their phase transition temperatures (Fig. 5a). The crystal of complex **2** shows significant anisotropic deformation upon phase transition. The horizontal length, vertical length and thickness are changed from 157 to

155 µm (−1%), from 93 to 98 µm (+5%) and from 22.6 to 21.4 µm (−6%), respectively. This macroscopic crystal deformation is clearly related to the changes at the molecular level as observed by single-crystal X-ray crystallography, i.e. the *a*, *b* and *c* axes for complex **2** change by −1%, +5% and −5%, respectively (Fig. 2b and Supplementary Table S1).

Crystals of all the complexes undergo similar crystal deformations upon thermal phase transition. However, the crystals of complexes **2** and **5** are fractured by the phase transition, particularly in the cooling process (Fig. 5b and supplementary Fig. S15). This is probably because of the relatively weak intermolecular interaction along the fracture direction formed by stacking of pseudorotaxane molecules. This interaction is not durable to the dramatic size change during the phase transition, leading to quasi-reversibility. In terms of the reversibility of the phase transition in pseudorotaxane crystals for switching operations, the use of complex **1** is beneficial to the preparation of mixed crystals due to its durability to the phase transition. Indeed, the reversibility of the thermal phase transition of the crystals is substantially improved in the mixed crystals, particularly in complexes **3** and **4** (Fig. 5b).

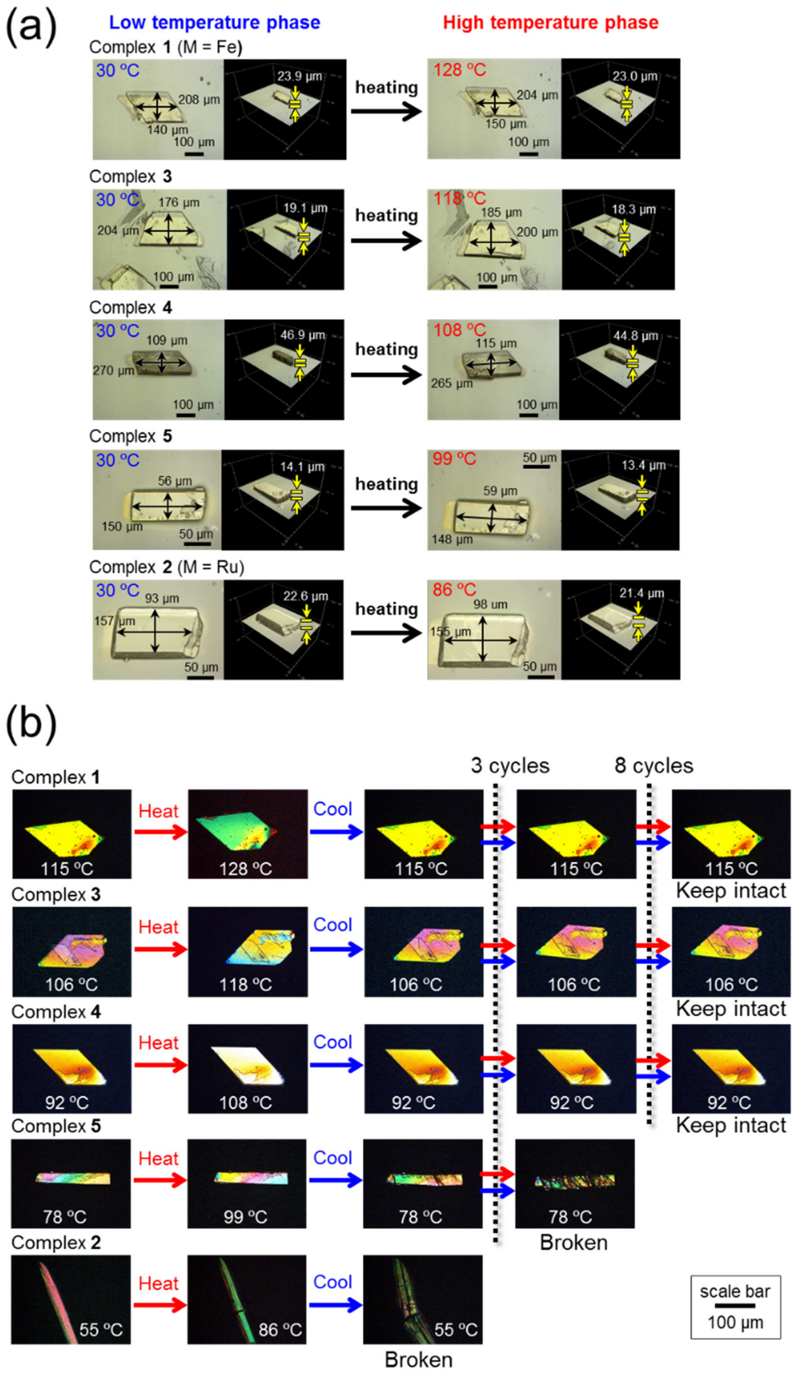

Figure 5. (**a**) Reflection micrographic images of crystals of complexes **1-5**. These images were observed using a confocal laser microscope. (**b**) Optical micrographs of single crystals of complexes **1-5** under cross polarised light. Crystals were repeatedly heated beyond the phase transition temperatures and cooled.

Photoinduced structural changes

Photoinduced switching of pseudorotaxane crystals was investigated. As shown in the schematic illustration in Fig. 6a, expansion/contraction of the crystals can be controlled by turning on/off laser irradiation at 405 nm or 445 nm at 30 °C (Path I). Furthermore, crystal-to-crystal thermal phase transition can be triggered by laser irradiation upon heating (Path II). These macroscopic crystal behaviours were observed using an optical microscope with/without cross polarised light under controlled temperatures.

Fig. 6b and Supplementary Fig. S16a show the laser-power dependence of the change in the relative area of the (001) facet, which is the top face of the single crystals, at 405 nm and 445 nm, respectively (Fig. 6a Path I). The magnitude of crystal expansion is significantly dependent on the power and wavelength of the laser. The crystal of complex **3** shows the highest magnitude of expansion, i.e. +1%, followed by **4** (+0.6%), **5** (+0.5%), **1** (+0.5%) and **2** (+0.3%), under 20 mW of 405 nm laser irradiation (Fig. 6b), whereas the order for 20 mW of 445 nm laser irradiation is **3** (+0.8%), **1** (+0.7%), **4** (+0.5%), **5** (+0.5%) and **2** (< +0.1%) (Supplementary Fig. S16a).

Fig. 6c shows the UV-visible spectra of complexes **1**–**5** in chloroform solutions. Complexes **1** and **2** exhibit absorption maxima at 440 and 320 nm, respectively, which

are assigned to metal-to-ligand charge transfer (MLCT) transitions of the ferrocenyl and ruthenocenyl group, respectively. Hence, the order of absorption intensity at 405 nm and 445 nm is **1**, **3**, **4**, **5** and **2**, which is also the order of ferrocenyl group content. Interestingly, this order is inconsistent with the magnitude of the expansion of the crystals, suggesting that the expansion efficiency is related to not only the photo absorption coefficient but also other parameters such as molecular packing, as discussed later.

Furthermore, the crystal-to-crystal phase transition temperatures were estimated with/without 10 mW 405 or 445 nm laser irradiation, based on observations using a cross polarised microscope, and these temperatures are plotted vs. Ru/(Fe + Ru) (Fig. 6a Path II, Fig. 6d and Supplementary Fig. S16b). The transition temperatures of all the complexes decrease upon laser irradiation. Among them, the mixed crystals of complexes **3** and **4** exhibit particularly large temperature drops (ΔT = 28 °C for complex **3** and ΔT = 24 °C for complex **4**) under 405 nm laser irradiation.

The photoinduced expansion/contraction and the decrease in the phase transition temperatures can be accounted for by the photo-thermal conversion of the crystals. In our previous study, the effect of laser irradiation on the surface temperature of the pseudorotaxane crystals was analysed through thermographic measurement, which showed that the crystal temperature rapidly increased by 10-15 °C by 445nm laser

irradiation[43]. This causes the photoactivation of the ferrocenyl group in the pseudorotaxanes, leading to the elongation of the crystal lattice in 3D as well as the photo-assisted thermal phase transitions.

To rationalise the higher dynamics of the mixed crystals than those of the pure crystals, an illustration of the simple packing models of the pseudorotaxanes is proposed in Fig. 6e. The crystals of complexes **1** and **2** have highly packed structures compared to those of the mixed crystals. Therefore, the motions of the pseudorotaxane molecules therein are lessened. Conversely, the addition of the bulky ruthenocene complex disrupts the packing in the mixed crystals of complexes **3–5**, and as a result, the mixed crystals exhibit relatively flexible packing and larger free volumes. Hence, the pseudorotaxane molecules with the less bulky ferrocenyl group exhibit higher dynamics. In addition, because the ferrocenyl group exhibits high absorbance at 405 and 445 nm, crystals of complex **3** with the high ferrocenyl composition ratio show the most significant dynamics upon laser irradiation at 405 and 445 nm in all pseudorotaxanes.

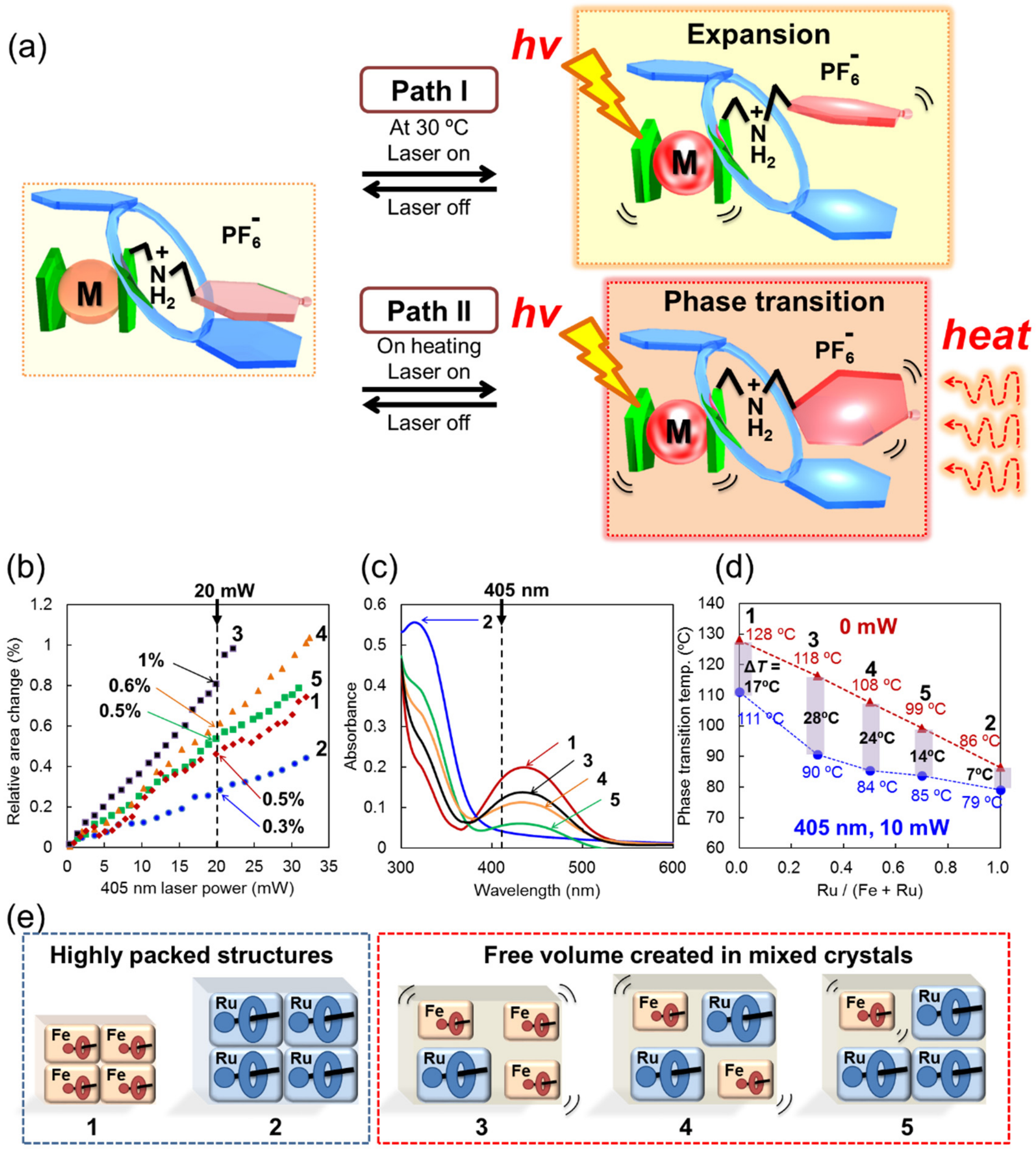

Figure 6. Photoresponsive behaviours of pseudorotaxane crystals. (**a**) Schematic of photoinduced structural changes. Pseudorotaxane is activated by laser irradiation, leading to expansion of the crystal at 30 °C (Path I) and crystal-to-crystal phase transition at high temperatures (Path II). (**b**) Laser-power dependence of the change in the relative area of the (001) facet of the single crystal induced by focused 405 nm laser irradiation. (**c**) UV-visible absorption spectra of mixed crystals in chloroform solutions. (**d**) Plots of phase transition temperature vs. Ru/(Fe + Ru) with/without 10 mW 405 nm laser irradiation. (**e**) Illustration of packing structures for pseudorotaxanes in the crystal state. Free space can be created by the presence of ruthenocene in the mixed crystals. See also the Supplementary Movie.

Photomechanical conversion

Next, the photoinduced deformations of the pseudorotaxane crystals are used for demonstrations of photomechanical conversions (Fig. 7 and Supplementary Fig. S17). Fig. 7a–7e shows illustrations and optical micrographs of various photo-triggered salient effects of crystals of complex **3**. Crystals of complex **3** were selected because it shows the highest sensitivity and good reversibility in the switching operations discussed above. The plate-shaped crystal shows reversible size expansion/contraction controlled by 405 nm irradiation with a weak 14 mW laser at 30 °C (Fig. 7b). In addition, a cluster of rod-shaped crystals exhibits a reversible scissoring motion controlled by 405 nm laser irradiation at a power of 16 mW (Fig. 7c). These motions are regular and the crystal integrity is completely retained. Furthermore, hopping of the crystals over a long distance and flipping over a short distance are triggered by 405 nm laser irradiation at a relatively strong laser power of 25 mW at 30 °C (Figs. 7d and 7e). These motions are drastic and irregular (i.e. stochastic). Therefore, they are probably induced by the crystal-to-crystal phase transition. In fact, complex **3** undergoes crystal-to-crystal phase transition even at 30 °C under 405 nm irradiation at a high laser power of 25 mW (Supplementary Fig. S18). After the hopping and flipping, the crystals still preserve their macroscopic integrity.

The forces accompanied by the photoinduced crystal expansion and phase transition

were measured at 30 and 85 °C for crystals of complexes **1–3** using a microforce analyser comprising two gauges on a cantilever (Figs. 7f and 7g and Supplementary Fig. S19). A 13 μg crystal of complex **3** provides 20 mg force when irradiated by 50 mW of 405 nm laser at 30 °C. This value corresponds to 1,600-times the crystal weight itself. Under the same observation condition, complex **3** provides the highest lifting weight ratio of the complexes analysed, followed by complexes **1** and **2**. This order is consistent with the expansion sensitivity to 405 nm laser irradiation (Fig. 6b).

Conversely, upon heating at 85 °C under 12 mW of 405 nm laser irradiation, a negative force is observed for 2.5 μg of a crystal of complex **3**. This is due to a decrease in the crystal thickness (*c* axis, Fig. 2b) upon phase transition, which is completed within only 4 ms (Supplementary Fig. S20). When the laser is turned off, the crystal immediately returns to its original form on the same timescale (4 ms, Supplementary Fig. S20), providing a high lifting weight of 16 mg that corresponds to a weight ratio of 6,400-times the crystal weight. For comparison, complex **1** was also irradiated under the same conditions. However, complex **1** exhibits expansion only because it has a much higher phase transition temperature than complex **3**. The observed weight ratio for the phase transition of complex **3** is 4 orders of magnitude higher than that previously observed for the expansion of complex **1** (a lifting/crystal weight ratio of ca. 1600) under 445 nm laser

irradiation[43], and 1–2 orders of magnitude higher than those previously reported for photochromic azobenzene polymer films[47, 48], diarylethene crystals[23, 49] or hydrogels with electro-triggered mobility[50] and polymer composite actuators driven by hydro gradients[51].

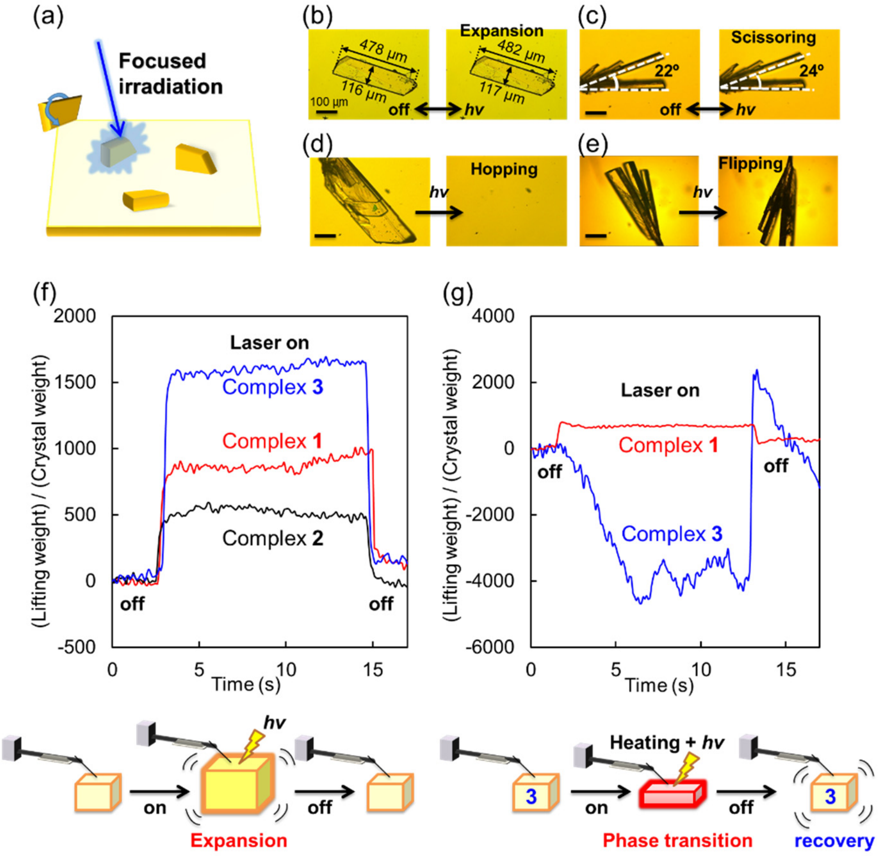

Figure 7. Photosalient effects. (**a**) Illustration of photosalient effect triggered by focused laser irradiation. (**b**) Photoinduced expansion of a crystal of complex **3** controlled by 405 nm laser irradiation (14 mW) at 30 °C. (**c**) Photoinduced scissoring motion of crystals of complex **3** controlled by 405 nm laser irradiation (16 mW) at 30 °C. (**d**) Hopping of a crystal of complex **3** triggered by 405 nm laser irradiation (25 mW) at 30 °C. (**e**) Flipping of crystals of complex **3** triggered by 405 nm laser irradiation (25 mW) at 30 °C. (**f**) Time dependence of lifting/crystal weight ratio of complexes **1**–**3** controlled by 405 nm laser irradiation (50 mW) at 30 °C. (**g**) Time dependence of lifting/crystal weight ratio of complexes **1** and **3** controlled by 405 nm laser irradiation (12 mW) at 85 °C. Illustration of the force detection is depicted. Scale bars represent 100 μm. See also the Supplementary Movie.

Conclusion

We have developed metallocene-containing dynamic pseudorotaxane crystals that respond to thermo- and photostimuli, transducing the incident energy into mechanical motions. These pseudorotaxanes are composed of multiple functional moieties: the ring molecule of the dibenzo crown ether acts as a flexible host with size and motion adjustability; the aromatic ring at one end of the axle molecule exhibits mechanical twisting dynamics, and the ferrocene at the other end of the axle molecule acts as a photosensitiser under 405 nm laser irradiation. Furthermore, substituting the ferrocene moiety with a bulky ruthenocene complex facilitates the twisting of the aromatic ring of the axle molecule. The structural changes of the pseudorotaxanes have been precisely observed by single-crystal X-ray crystallography, providing a detailed rationale and a strategy for the molecular design of interlocked molecules that accumulate molecular motion and express them as macroscopic dynamics in the crystal state. We expect that such unique dynamic interlocked molecules in the crystal state can be applied as molecular actuators and switches in stimuli-responsive mechanical components.

Methods

General methods. Synthesis and crystallization of the pseudorotaxane were performed on the basis of the method described in the literature[52]. Ruthenocene, sodium borohydride ($NaBH_4$) and DB24C8 were purchased from Sigma-Aldrich. Anhydrous DMF, toluene, MeOH and THF were purchased from Aldrich in Sure/Seal container and were stored under N_2 gas. 4-Methylbenzylamine was purchased from Alfa Aesar. ^{1}H NMR spectra were record on Varian-Unity INOVA-500 spectrometer. The chemical shifts were referenced with respect to $CHCl_3$ ($\delta_H = 7.24$) and CD_2HCN ($\delta_H = 1.93$) for ^{1}H as internal standards. Elemental analysis was carried out with a CHN-O-Rapid elemental analyzer (Foss. Heraeus, Germany). Fast atom bombardment mass spectra (FAB-MS) were obtained with Micromass Trio 2000 using 3-nitrobenzyl alcohol as the matrix. DSC curves were recorded by a Perkin Elmer Diamond DSC instrument using heating and cooling rates of 10 °C min^{-1}. UV-visible absorption spectra were measured using a JASCO V-630 spectrophotometer.

Synthesis of ruthenocenylmethyl(4-methylphenyl)ammonium axle molecule. *p*-xylylaminomethylruthenocene (163.6 mg, 0.45 mmol) in 6N HCl aq. (10 mL) was treated with ultrasonication for a few minutes to prepare a suspension. This was then stirred at a room temperature for 1 h. The residual brown powder was washed with water (20 mL) then dried in vacuum. To a suspension of [ruthenocenylmethyl(4-methylphenyl) ammonium]$^+$Cl$^-$ in acetone (150 mL), NH_4PF_6 (93.5 mg, 0.58 mmol) was added, and the mixture was stirred at a room temperature for 1 h. The precipitate was collected by filtration and dried in vacuum to give [ruthenocenylmethyl(4-methylphenyl) ammonium]$^+$(PF_6)$^-$ (133.4 mg, 58%) as a light brown powder. ^{1}H NMR spectrum (500 MHz, CD_3CN, r.t.): δ 2.32 (s, 3H, Me), 3.78 (br, 2H, CH_2), 4.10 (br, 2H, CH_2), 4.58 (s,

5H, C_5H_5), 4.62 (m, 2H, C_5H_4), 4.75 (m, 2H, C_5H_4), 7.26 (d, 2H, C_6H_4, J = 7.5 Hz), 7.32 (d, 2H, C_6H_4, J = 7.5 Hz) (Supplementary Fig. S2). ^{13}CNMR (500 MHz, CD_3CN): δ 21.25 (Me), 47.76, 51.83 (CH_2), 72.14 (C_5H_5), 72.31, 73.33, 80.89 (C_5H_4), 128.45, 130.70, 131.01, 140.93 (C_6H4) (Supplementary Fig. S3). HRMS (FD) calcd. for $C_{19}H_{22}NRu$ $[M+H]^+$ (m/z): 366.0790, found: 366.0780 (error 2.7 ppm) (Supplementary Fig. S5). Anal. Calcd. for $C_{19}H_{22}NF_6RuP$: C, 44.70; H, 4.30; N, 2.75. Found: C, 44.53; H, 4.75; N, 2.85.

Preparation of [ruthenocenylmethyl(4-methylphenyl)ammonium·DB24C8]$^+$(PF_6)$^-$ rotaxane crystal: [Ruthenocenylmethyl(4-methylphenyl)ammonium]$^+$(PF_6)$^-$ (0.028 mmol, 14.4 mg) and DB24C8 (0.028 mmol, 14.3 mg) were dissolved in CH_2Cl_2. This solution was then placed in Et_2O vapour atmosphere at a room temperature for 24 h to give light yellow crystals of [ruthenocenylmethyl(4-methylphenyl)ammonium ·DB24C8]$^+$(PF_6)$^-$ (19.5 mg, 0.020 mmol, 73% yield). ^{1}H NMR spectrum (500 MHz, CD_3CN, r.t.): δ 2.11-2.13* (s, 3H, Me), 3.55-3.70* (m, 8H, OCH_2), 3.70-3.90* (m, 8H, OCH_2), 4.04-4.21* (m, 8H, OCH_2), 4.19 (m, 2H, NCH_2), 4.33 (s, 5H, Cp), 4.38 (s, 2H, Cp), 4.58 (m, 2H, NCH_2), 6.87-6.95* (m, 8H, C_6H_4 (host)), 7.15 (d, 2H, C_6H_4, J = 8 Hz) (Supplementary Fig. S8). The peaks with asterisks are significantly overlapped with other signals. The complex/uncomplex ratio in CD_3CN solution is determined to be 1.29 by the comparison of the integrated peak area of cyclopentadienyl protons and methylene protons. ^{13}CNMR (500 MHz, CD_3CN): δ 21.24 (uc, Me), 30.88 (c, Me) 47.71, 51.77 (uc, CH_2), 49.24, 52.77 (c, CH_2), 71.94 (uc, C_5H_5), 71.85 (c, C_5H_5), 72.27, 73.30, 80.94 (uc, C_5H_4), 72.12, 73.22, 81.10 (c, C_5H_4), 129.98, 130.22, 130.96, 140.84 (uc, C_6H4), 128.95, 130.22, 130.67, 139.83 (c, C_6H4), 69.06, 69.75, 70.27, 71.18, 71.27, 71.56 (OCH_2), 113.67, 115.33, 122.28, 122.52, 148.72, 149.77 (C_6H_4) (Supplementary Fig. S9). HRMS (FD) calcd. for $C_{43}H_{54}NO_8Ru$ $[M+H]^+$ (m/z): 814.2887, found: 814.29091 (error 1.7 ppm)

(Supplementary Fig. S7). Anal. Calcd. for $C_{43}H_{54}NO_8F_6RuP$: C, 53.80; H, 5.63; N, 1.46. Found: C, 53.82; H, 5.67; N, 1.34.

Single-crystal X-ray crystallography. Single-crystal X-ray crystallography was performed for the pseudorotaxane crystals (Bruker, APEX DUO, Dual Wavelength System, 60 Hz). An APEX II 4K CCD Detector was used for data collection, and the corresponding Bruker APEX II software was used for data collection and reduction. An OXFORD, Cryostream 800 Plus seamless sample cooling and heating system was used to control the measurement temperature. The structures were solved using SHELXL-2014/6.

Optical microscopy. A microscope (Olympus, BX51) equipped with a temperature-controlled stage (TMS 92 and HMS600, both from Linkam Co.) was used to monitor the crystal-to-crystal phase transitions. A crystal was placed on the stage, and the stage was heated or cooled a rate of 2 °C min^{-1}. The Imagesource DFK 51AU02 charge-coupled device (CCD) camera with a video rate of 12 fps was used to capture the images and movies of a crystal. A confocal laser microscope with a 50X objective lens (Keyence VK-9500) was used to measure the crystal dimensions.

Photomechanical measurement. Continuous-wave 405-nm and 445-nm diode pump solid state lasers (TAN-YU, T405F200 and LSR445FP-1W) were used in this experiment. A multiple-optical-fibre cable was connected as an output light source and the light illuminated from the source was aligned by a SMA 905 collimator. The power of the 405 nm or 445 nm laser beam passing through the objective lens was tuned by adjusting the amperage from the controller, and was measured using a power meter (OPHIR, Nova II P/N7Z01550). After passing through a 20X objective lens with numerical aperture (NA) = 0.25, the focused laser beam spot diameter was approximately 1 μm. The crystals were

observed using the optical microscope (Olympus, BX51) equipped with the Image source DFK 51AU02 CCD camera or pco.dimax S1 high speed CMOS camera (1008^2 pixel, 1000 fps) with CamWare V3.17 software to capture the images and movies of the crystals.

Force measurement. A microforce detector composed of a cantilever attached to two strain gauge on both sides (CHIEF SI, μ-force) and the Bridge DAQ software were used to measure the lifting weight of the single crystals of pseudorotaxane complexes. During the measurement, all equipments were placed on a vibration isolation table to prevent any possible vibration. A continuous-wave 405-nm laser (TAN-YU, T405F200) was used with an optical microscope (Olympus, BX51) with a 4X objective lens (NA = 0.1). Temperature of crystals were controlled using TMS 92 and HMS600 (Linkam Co.).

Data availability. Data supporting the findings of this study are available within the article (and its Supplementary Information files) and from the corresponding author on reasonable request. Crystallographic data have been deposited at the CCDC and copies can be obtained on request, free of charge, by quoting the publication citation and the deposition numbers 1546042-1546057 and 1551312.

Reference.

1. Vogelsberg, C. S. & Garcia-Garibay, M. A., Crystalline molecular machines: function, phase order, dimensionality, and composition. *Chem. Soc. Rev.* **41**, 1892-1910 (2012).

2. Kudernac, T. *et al.*, Electrically driven directional motion of a four-wheeled molecule on a metal surface. *Nature* **479**, 208-211 (2011).

3. Jiang, X., Rodríguez-Molina, B., Nazarian, N. & Garcia-Garibay, M. A., Rotation of a Bulky Triptycene in the Solid State: Toward Engineered Nanoscale Artificial Molecular Machines. *J. Am. Chem. Soc.* **136**, 8871-8874 (2014).

4. Maggi, C., Saglimbeni, F., Dipalo, M., De Angelis, F. & Di Leonardo, R., Micromotors with asymmetric shape that efficiently convert light into work by thermocapillary effects. *Nat. Commun.* **6**, 7855 (2015).

5. Kassem, S., Lee, A. T. L., Leigh, D. A., Markevicius, A. & Solà, J., Pick-up, transport and release of a molecular cargo using a small-molecule robotic arm. *Nat. Chem.* **8**, 138-143 (2016).

6. Zhang, Y. *et al.*, Simultaneous and coordinated rotational switching of all molecular rotors in a network. *Nat. Nanotech.* **11**, 706-712 (2016).

7. Erbas-Cakmak, S., Leigh, D. A., McTernan, C. T. & Nussbaumer, A. L., Artificial Molecular Machines. *Chem. Rev.* **115**, 10081-10206 (2015).

8. Cheng, C. *et al.*, An artificial molecular pump. *Nat Nanotech.* **10**, 547-553 (2015).

9. Lewandowski, B. *et al.*, Sequence-Specific Peptide Synthesis by an Artificial Small-Molecule Machine. *Science* **339**, 189-193 (2013).

10. Coskun, A., Banaszak, M., Astumian, R. D., Stoddart, J. F. & Grzybowski, B. A., Great expectations: can artificial molecular machines deliver on their promise? *Chem. Soc. Rev.* **41**, 19-30 (2012).

11. Bruns, C. J. & Stoddart, J. F., Rotaxane-Based Molecular Muscles. *Acc. Chem. Res.* **47**, 2186-2199 (2014).

12. Iwaso, K., Takashima, Y. & Harada, A., Fast response dry-type artificial molecular muscles with [c2]daisy chains. *Nat. Chem.* **8**, 625-632 (2016).

13. Tayi, A. S., Kaeser, A., Matsumoto, M., Aida, T. & Stupp, S. I., Supramolecular ferroelectrics. *Nat. Chem.* **7**, 281-294 (2015).

14. Zhu, K., O'Keefe, C. A., Vukotic, V. N., Schurko, R. W. & Loeb, S. J., A molecular shuttle that operates inside a metal–organic framework. *Nat. Chem.* **7**, 514-519 (2015).

15. Coskun, A. *et al.*, Metal–Organic Frameworks Incorporating Copper-Complexed Rotaxanes. *Angew. Chem. Int. Ed.* **51**, 2160-2163 (2012).

16. Han, Y. *et al.*, Formation of a series of stable pillar[5]arene-based pseudo[1]-rotaxanes and their [1]rotaxanes in the crystal state. *Sci. Rep.* **6**, 28748 (2016).

17. Panda, M. K. *et al.*, Strong and Anomalous Thermal Expansion Precedes the Thermosalient Effect in Dynamic Molecular Crystals. *Sci. Rep.* **6**, 29610 (2016).

18. Shima, T. *et al.*, Thermally Driven Polymorphic Transition Prompting a Naked-Eye-Detectable Bending and Straightening Motion of Single Crystals. *Angew. Chem. Int. Ed.* **53**, 7173-7178 (2014).

19. Centore, R. *et al.*, A series of compounds forming polar crystals and showing single-crystal-to-single-crystal transitions between polar phases. *Cryst. Eng. Comm.* **14**, 2645-2653 (2012).

20. Nath, N. K. *et al.*, Surface and Bulk Effects in Photochemical Reactions and Photomechanical Effects in Dynamic Molecular Crystals. *J. Am. Chem. Soc.* **137**, 13866-13875 (2015).

21. Seki, T., Sakurada, K., Muromoto, M. & Ito, H., Photoinduced single-crystal-to-single-crystal phase transition and photosalient effect of a gold(i) isocyanide complex with shortening of intermolecular aurophilic bonds. *Chem. Sci.* **6**, 1491-1497 (2015).

22. Taniguchi, T., Fujisawa, J., Shiro, M., Koshima, H. & Asahi, T., Mechanical Motion of Chiral Azobenzene Crystals with Twisting upon Photoirradiation. *Chem. Eur. J.* **22**, 7950-7958 (2016).

23. Terao, F., Morimoto, M. & Irie, M., Light-Driven Molecular-Crystal Actuators: Rapid and Reversible Bending of Rodlike Mixed Crystals of Diarylethene Derivatives. *Angew. Chem. Int. Ed.* **124**, 925-928 (2012).

24. Shepherd, H. J. *et al.*, Molecular actuators driven by cooperative spin-state switching. *Nat. Commun.* **4**, 2607 (2013).

25. Sahoo, S. C. *et al.*, Kinematic and Mechanical Profile of the Self-Actuation of Thermosalient Crystal Twins of 1,2,4,5-Tetrabromobenzene: A Molecular

Crystalline Analogue of a Bimetallic Strip. *J. Am. Chem. Soc.* **135**, 13843-13850 (2013).

26. Naumov, P., Sahoo, S. C., Zakharov, B. A. & Boldyreva, E. V., Dynamic Single Crystals: Kinematic Analysis of Photoinduced Crystal Jumping (The Photosalient Effect). *Angew. Chem. Int. Ed.* **52**, 9990-9995 (2013).

27. Naumov, P., Chizhik, S., Panda, M. K., Nath, N. K. & Boldyreva, E., Mechanically Responsive Molecular Crystals. *Chem. Rev.* **115**, 12440-12490 (2015).

28. Commins, P., Desta, I. T., Karothu, D. P., Panda, M. K. & Naumov, P., Crystals on the move: mechanical effects in dynamic solids. *Chem. Commun.* **52**, 13942-13954 (2016).

29. Shtukenberg, A. G., Punin, Y. O., Gujral, A. & Kahr, B., Growth Actuated Bending and Twisting of Single Crystals. *Angew. Chem. Int. Ed.* **53**, 672-699 (2014).

30. Han, D.-D. *et al.*, Light-Mediated Manufacture and Manipulation of Actuators. *Adv. Mater.* **28**, 8328-8343 (2016).

31. Yao, Z.-S. *et al.*, Molecular motor-driven abrupt anisotropic shape change in a single crystal of a Ni complex. *Nat. Chem.* **6**, 1079-1083 (2014).

32. Sato, O., Dynamic molecular crystals with switchable physical properties. *Nat. Chem.* **8**, 644-656 (2016).

33. Su, S.-Q. *et al.*, Assembling an alkyl rotor to access abrupt and reversible crystalline deformation of a cobalt(II) complex. *Nat. Commun.* **6**, 8810 (2015).

34. Yangui, A. *et al.*, Rapid and robust spatiotemporal dynamics of the first-order phase transition in crystals of the organic-inorganic perovskite $(C_{12}H_{25}NH_3)_2PbI_4$. *Sci. Rep.* **5**, 16634 (2015).

35. Medishetty, R., Sahoo, S. C., Mulijanto, C. E., Naumov, P. & Vittal, J. J., Photosalient Behavior of Photoreactive Crystals. *Chem. Mater.* **27**, 1821-1829 (2015).

36. Medishetty, R. *et al.*, Single Crystals Popping Under UV Light: A Photosalient Effect Triggered by a [2+2] Cycloaddition Reaction. *Angew. Chem. Int. Ed.* **53**, 5907-5911 (2014).

37. Sun, J.-K. *et al.*, Photoinduced Bending of a Large Single Crystal of a 1,2-Bis(4-pyridyl)ethylene-Based Pyridinium Salt Powered by a [2+2] Cycloaddition.

Angew. Chem. Int. Ed. **52**, 6653-6657 (2013).

38. Guo, X., Zhou, J., Siegler, M. A., Bragg, A. E. & Katz, H. E., Visible-Light-Triggered Molecular Photoswitch Based on Reversible E/Z Isomerization of a 1,2-Dicyanoethene Derivative. *Angew. Chem. Int. Ed.* **54**, 4782-4786 (2015).

39. Bushuyev, O. S., Friscic, T. & Barrett, C. J., Photo-induced motion of azo dyes in organized media: from single and liquid crystals, to MOFs and machines. *Cryst. Eng. Comm.* **18**, 7204-7211 (2016).

40. Irie, M., Fukaminato, T., Matsuda, K. & Kobatake, S., Photochromism of Diarylethene Molecules and Crystals: Memories, Switches, and Actuators. *Chem. Rev.* **114**, 12174-12277 (2014).

41. Horie, M. *et al.*, A Crystalline Supramolecular Switch: Controlling the Optical Anisotropy through the Collective Dynamic Motion of Molecules. *Angew. Chem. Int. Ed.* **46**, 4983-4986 (2007).

42. Horie, M. *et al.*, Thermally-Induced Phase Transition of Pseudorotaxane Crystals: Changes in Conformation and Interaction of the Molecules and Optical Properties of the Crystals. *J. Am. Chem. Soc.* **134**, 17932-17944 (2012).

43. Chen, K.-J. *et al.*, Rapid and reversible photoinduced switching of a rotaxane crystal. *Nat. Commun.* **7**, 13321 (2016).

44. Yang, C., Wang, X. & Omary, M. A., Crystallographic Observation of Dynamic Gas Adsorption Sites and Thermal Expansion in a Breathable Fluorous Metal–Organic Framework. *Angew. Chem. Int. Ed.* **48**, 2500-2505 (2009).

45. Das, D., Jacobs, T. & Barbour, L. J., Exceptionally large positive and negative anisotropic thermal expansion of an organic crystalline material. *Nat. Mater.* **9**, 36-39 (2010).

46. Panda, M. K. *et al.*, Colossal positive and negative thermal expansion and thermosalient effect in a pentamorphic organometallic martensite. *Nat. Commun.* **5**, 4811-4818 (2014).

47. Zhang, L., Liang, H., Jacob, J. & Naumov, P., Photogated humidity-driven motility. *Nat. Commun.* **6**, 7429 (2015).

48. Cheng, F., Yin, R., Zhang, Y., Yen, C.-C. & Yu, Y., Fully plastic microrobots which manipulate objects using only visible light. *Soft Matter.* **6**, 3447-3449 (2010).

49. Morimoto, M. & Irie, M., A Diarylethene Cocrystal that Converts Light into Mechanical Work. *J. Am. Chem. Soc.* **132**, 14172-14178 (2010).

50. Yang, C. *et al.*, Hydrogel Walkers with Electro-Driven Motility for Cargo Transport. *Sci. Rep.* **5**, 13622 (2015).

51. Ma, M., Guo, L., Anderson, D. G. & Langer, R., Bio-inspired Polymer Composite Actuator and Generator Driven by Water Gradients. *Science* **339**, 186-189 (2013).

52. Horie, M., Suzaki, Y. & Osakada, K., Chemical and Electrochemical Formation of Pseudorotaxanes Composed of Alkyl(ferrocenylmethyl)ammmonium and Dibenzo[24]crown-8. *Inorg. Chem.* **44**, 5844-5853 (2005).

End notes

Acknowledgments

We are grateful to Prof. K. Osakada and Dr. Y. Suzaki of Tokyo Institute of Technology for enlightening discussion. This work was financially supported by Ministry of Science and Technology Taiwan and Frontier Research Center on Fundamental and Applied Sciences of Matters at National Tsing Hua University Taiwan.

Author contributions

M.H. designed the study. K-J.C. carried out the most experiments. P-L.C. conducted the X-ray measurements. K-J.C. and M.H. wrote and edited the manuscript.

Additional information

Supplementary Information accompanies this paper at http://www.nature.com/.

Correspondence and requests for materials should be addressed to M.H.

Competing financial interests: The authors declare no competing financial interests.

Reprints and permission information is available online at http://npg.nature.com/reprintsandpermissions/

How to cite this article: Chen, K.-J. *et al.* Dynamic pseudorotaxane crystals containing metallocene complexes.

Figure legends

Figure 1. Pseudorotaxanes composed of Fe and Ru complexes. (**a**) Formation of pseudorotaxane molecules with different Fe and Ru ratios in dichloromethane solutions. (**b**) Illustration of stimuli-responsive dynamic pseudorotaxanes in the crystal state.

Figure 2. Thermal phase transition of pseudorotaxane crystals. (**a**) Optical micrographs of single crystals of complexes **1** and **2** without/with cross polarised light. (**b**) Volume and unit cell parameters of complexes **1** and **2** at different temperatures. (**c**) Overlays of the molecular structures of complexes **1** and **2** obtained by single-crystal X-ray crystallography at 30 °C, 128 °C (for complex **1**) and 86 °C (for complex **2**). See also the Supplementary Movie. (**d**) Schematic illustration of the molecular structural changes of complexes **1** and **2** on heating. Specific distances and angles between aromatic rings are depicted.

Figure 3. Thermal properties of mixed crystals. (**a**) DSC heating and cooling scans for complexes **1–5** at a scan rate of 5 °C min^{-1}. (**b**) Plots of crystal-to-crystal phase transition temperatures vs. molar fraction, i.e. Ru/(Fe + Ru).

Figure 4. Plots of intra-/intermolecular distances and angles vs. Ru/(Fe + Ru). Distances between. (**a**) Cp1 and aromatic ring A, (**b**) Cp1 and aromatic ring B, (**c**) aromatic rings A and B. (**d**) Angle between aromatic rings A and B. **e,** Schematic illustration of the molecular structural changes of complexes **1-5** on heating.

Figure 5. (**a**) Reflection micrographic images of crystals of complexes **1-5**. These images were observed using a confocal laser microscope. (**b**) Optical micrographs of single crystals of complexes **1-5** under cross polarised light. Crystals were repeatedly heated beyond the phase transition temperatures and cooled.

Figure 6. Photoresponsive behaviours of pseudorotaxane crystals. (**a**) Schematic of photoinduced structural changes. Pseudorotaxane is activated by laser irradiation, leading to expansion of the crystal at 30 °C (Path I) and crystal-to-crystal phase transition at high temperatures (Path II). (**b**) Laser-power dependence of the change in the relative area of the (001) facet of the single crystal induced by focused 405 nm laser irradiation. (**c**) UV-visible absorption spectra of mixed crystals in chloroform solutions. (**d**) Plots of phase transition temperature vs. Ru/(Fe + Ru) with/without 10 mW 405 nm laser irradiation. (**e**) Illustration of packing structures for pseudorotaxanes in the crystal state. Free space can be created by the presence of ruthenocene in the mixed crystals. See also the Supplementary Movie.

Figure 7. Photosalient effects. (**a**) Illustration of photosalient effect triggered by focused laser irradiation. (**b**) Photoinduced expansion of a crystal of complex **3** controlled by 405 nm laser irradiation (14 mW) at 30 °C. (**c**) Photoinduced scissoring motion of crystals of complex **3** controlled by 405 nm laser irradiation (16 mW) at 30 °C. (**d**) Hopping of a crystal of complex **3** triggered by 405 nm laser irradiation (25 mW) at 30 °C. (**e**) Flipping of crystals of complex **3** triggered by 405 nm laser irradiation (25 mW) at 30 °C. (**f**) Time dependence of lifting/crystal weight ratio of complexes **1–3** controlled by 405 nm laser irradiation (50 mW) at 30 °C. (**g**) Time dependence of lifting/crystal weight ratio of complexes **1** and **3** controlled by 405 nm laser irradiation (12 mW) at 85 °C. Illustration of the force detection is depicted. Scale bars represent 100 μm. See also the Supplementary Movie.

Supplementary Information for

Dynamic Pseudorotaxane Crystals Containing Metallocene Complexes

Kai-Jen Chen [†], Pei-Lin Chen[‡] and Masaki Horie *[†]

†*Department of Chemical Engineering, National Tsing Hua University, 101, Sec. 2, Kuang-Fu Road, Hsinchu, 30013,*

Taiwan

‡*Instrumentation Center, National Tsing Hua University, 101, Sec. 2, Kuang-Fu Road, Hsinchu, 30013, Taiwan*

Correspondence Address

Dr. Masaki Horie

Department of Chemical Engineering, National Tsing-Hua University

101, Sec. 2, Kuang-Fu Road, Hsin-Chu, 30013 Taiwan

E-mail: mhorie@mx.nthu.edu.tw

1) Synthesis of Ruthenocenecarboxaldehyde:

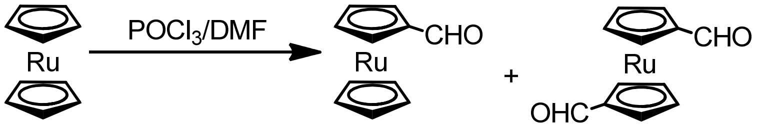

2) Synthesis of axle:

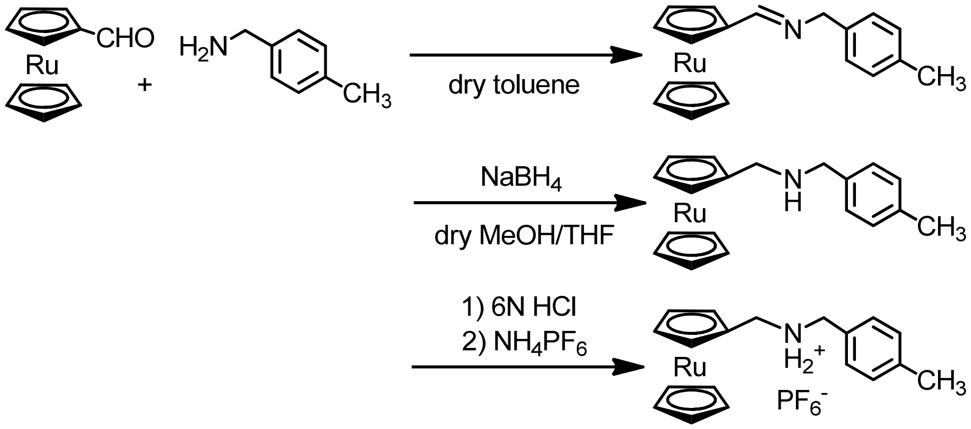

3) synthesis of pseudorotaxane:

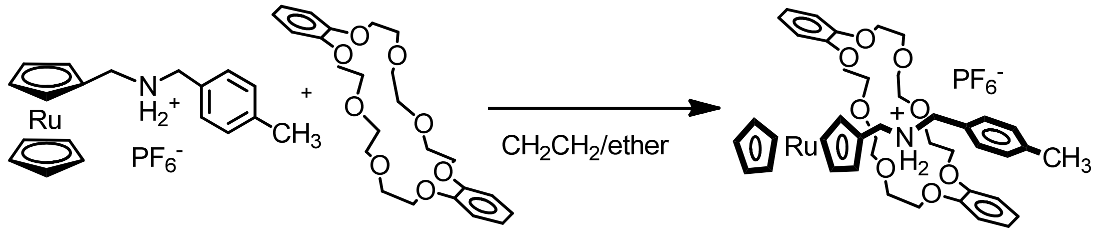

Supplementary Figure S1. Synthesis of complex **2**

Synthesis of Ruthenocenecarboxaldehyde:

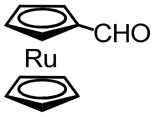

Vilsmeier–Haack reaction was used to synthesise ruthenocenecarboxaldehyde. In a Schlenk tube, $POCl_3$ (1.86 mL, 19.8 mmol) was added into DMF (1.55 mL, 19.6 mmol) at 0 ºC and stirred for 30 min. $POCl_3$/DMF solution was slowly added into ruthenocene (800 mg, 3.45 mmol) in 10 mL CH_2Cl_2 and the mixture was stirred at 80 ºC for 2 h. The mixture was cooled to a room temperature, and was slowly poured into 80 mL water. After stirring for 30 min, the mixture was partitioned between 1N NH_4Cl aq. (100 mL) and CH_2Cl_2 (100 mL). The organic extract was dried over $MgSO_4$, filtered, and concentrated under reduced pressure to give crude product. This was then purified by column chromatography (SiO_2/CH_2Cl_2) and dried in vacuum to give ruthenocene carboxaldehyde (170 mg, 19%) as a golden yellow solid. ^{1}H NMR spectrum (500 MHz, $CDCl_3$, r.t.): δ 4.63 (s, 5H, C_5H_5), 4.84 (m, 2H, C_5H_4), 5.07 (m, 2H, C_5H_4), 9.71 (d, 1H, CHO, J = 7.5 Hz).

Synthesis of *p*-xylyliminomethylruthenocene:

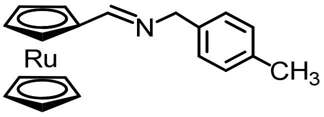

Ruthenocenecarboxaldehyde (300 mg, 1.154 mmol) and 4-ethylbenzylamine (0.147 mL, 1.154 mmol) were dissolved in dry toluene (17.5 mL) and stirred at a room temperature for 18 h. The solvent was removed under reduced pressure to provide *p*-xylyliminomethylruthenocene (414 mg, 99%) as a light brown solid. ^{1}H NMR spectrum (500 MHz, $CDCl_3$, r.t.): δ 2.31 (s, 3H, Me), 4.54 (s, 5H, C_5H_5), 4.53 (m, 2H, C_5H_4), 4.67 (s, 2H, NCH_2), 5.01 (m, 2H, C_5H_4), 7.10 (d, 2H, C_6H_4, J = 7.5 Hz), 7.11 (d, 2H, C_6H_4, J = 7.5 Hz), 8.03 (s, 1H, CH).

Synthesis of *p*-xylylaminomethylruthenocene:

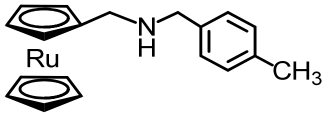

p-Xylyliminomethylruthenocene (386 mg, 1.06 mmol) was dissolved in dry THF/MeOH (24 mL:40 mL). $NaBH_4$ (40.9 mg, 1.08 mmol) was added to the solution, and stirred at a room temperature for 2 h. Further portion of $NaBH_4$ (40.9 mg, 1.08 mmol) was added to the reaction mixture. After stirring for 12 h, the reaction was quenched by addition of 1N HCl aq. (2 mL). The solvent was partitioned between 1N KOH aq. (10 mL) and CH_2Cl_2 (10 mL). The organic extract was dried over $MgSO_4$, filtered, and concentrated under reduced pressure to give *p*-xylylaminomethylruthenocene (376.6 mg, 96%) as dark brown oil. ^1H NMR spectrum (500 MHz, $CDCl_3$, r.t.): δ 2.28 (s, 3H, Me), 3.61 (br, 2H, CH_2), 3.89 (br, 2H, CH_2), 4.49 (s, 5H, C_5H_5), 4.53 (m, 2H, C_5H_4), 4.80 (m, 2H, C_5H_4), 7.16 (d, 2H, C_6H_4, J = 7.5 Hz), 7.40 (d, 2H, C_6H_4, J = 7.5 Hz).

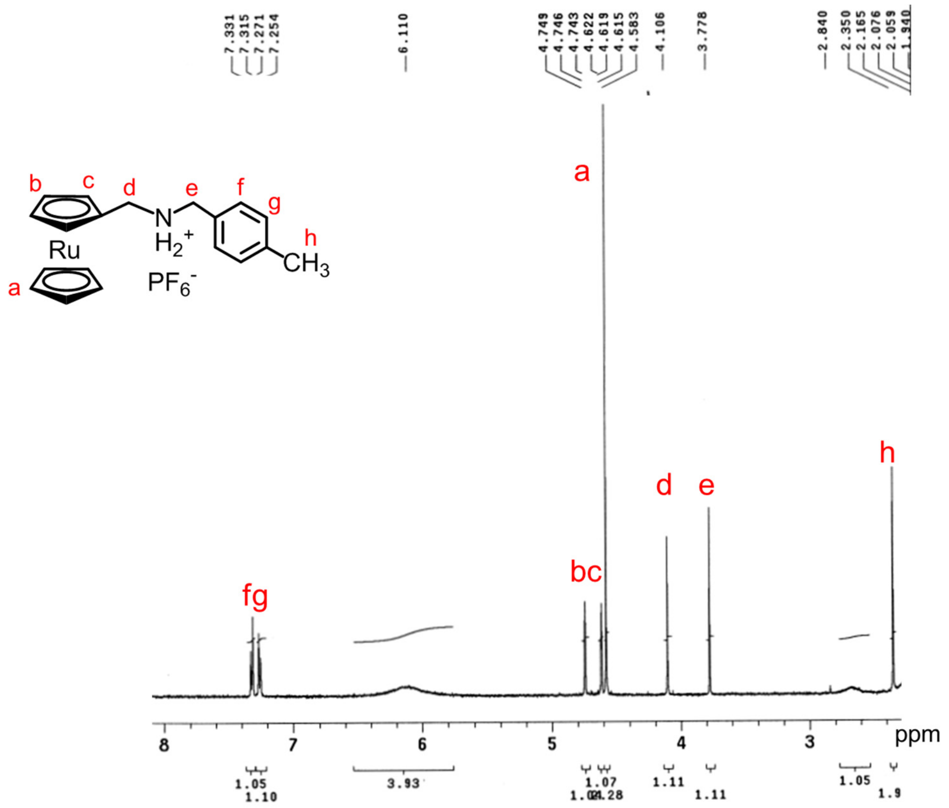

Supplementary Figure S2. ^1H NMR spectrum (500 MHz, CD_3CN) of [ruthenocenylmethyl (4-methylphenyl) ammonium]$^+(PF_6)^-$.

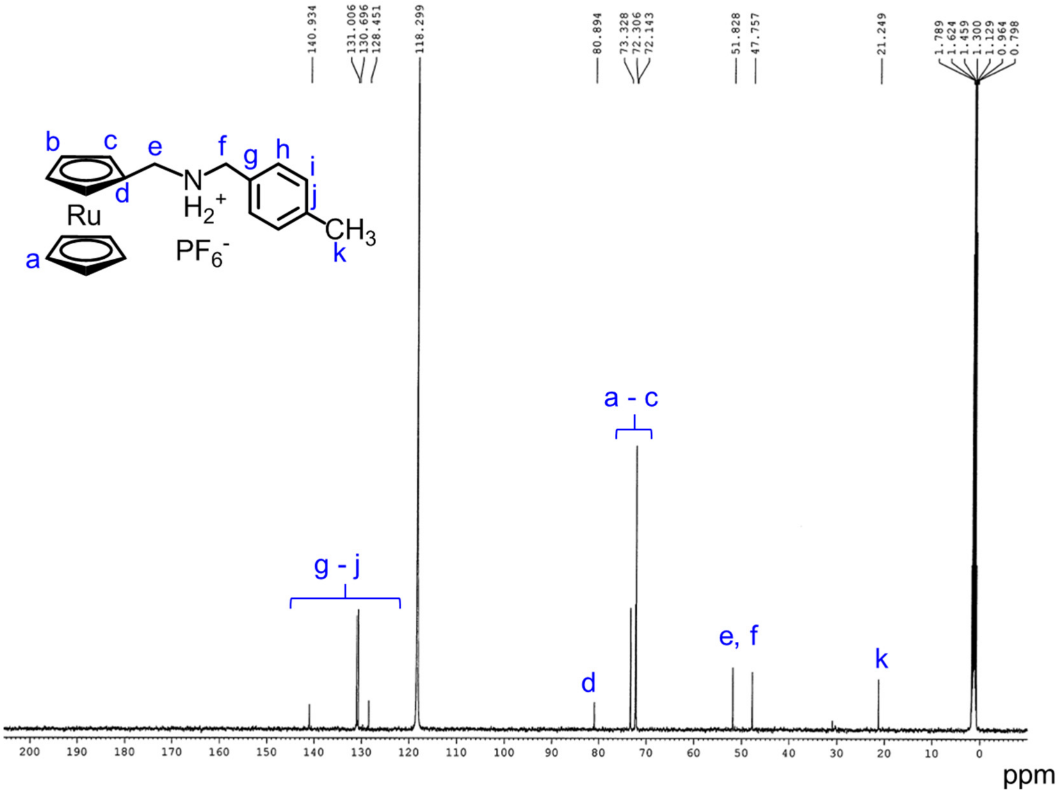

Supplementary Figure S3. ^{13}C NMR spectrum (125 MHz, CD_3CN) of [ruthenocenylmethyl (4-methylphenyl) ammonium]$^+(PF_6)^-$.

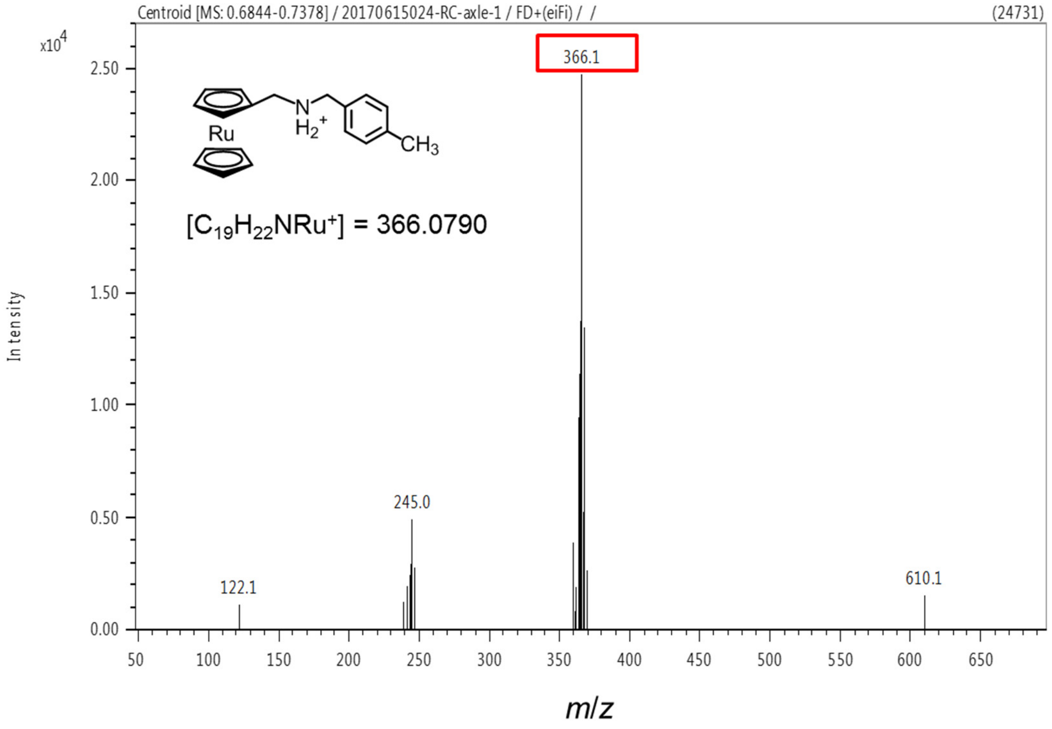

Supplementary Figure S4. LRFD mass spectra of [ruthenocenylmethyl(4methylphenyl) ammonium]$^+$(PF$_6$)$^-$. The main peak at m/z = 366.1 corresponds to [ruthenocenylmethyl(4-methylphenyl) ammonium]$^+$ ([C$_{19}$H$_{22}$NRu$^+$] =366.0790, error 57 ppm).

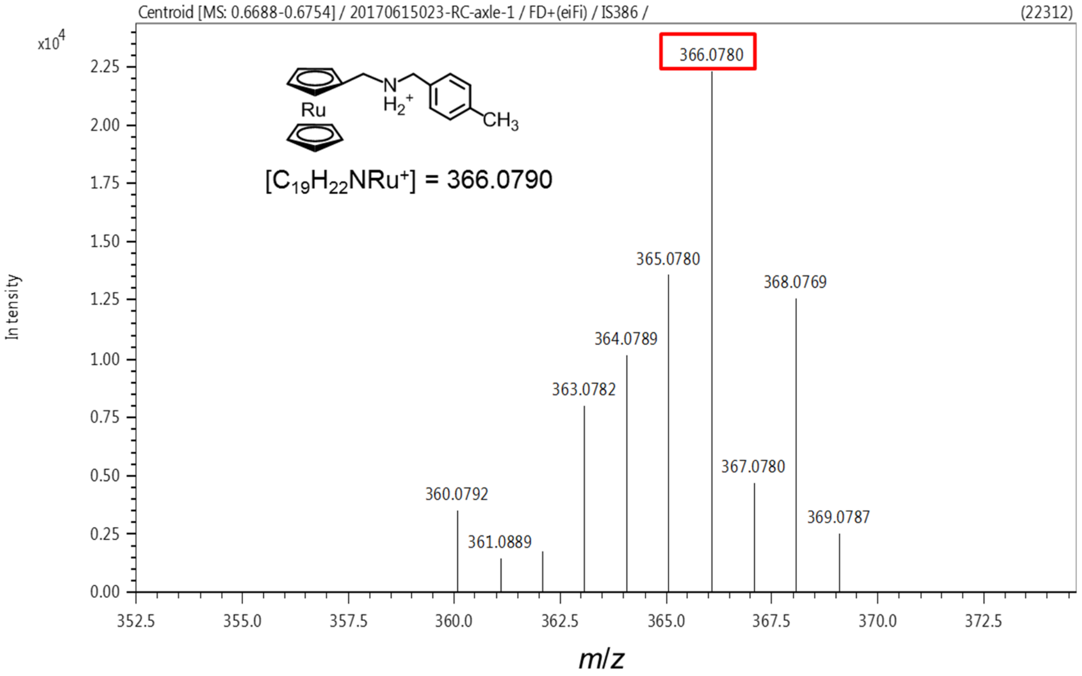

Supplementary Figure S5. HRFD mass spectra of [ruthenocenylmethyl(4methylphenyl) ammonium]$^+$(PF$_6$)$^-$. The main peak at m/z = 366.0780 corresponds to [ruthenocenylmethyl(4-methylphenyl)ammonium]$^+$ ([C$_{19}$H$_{22}$NRu$^+$] =366.0790, error 2.7 ppm).

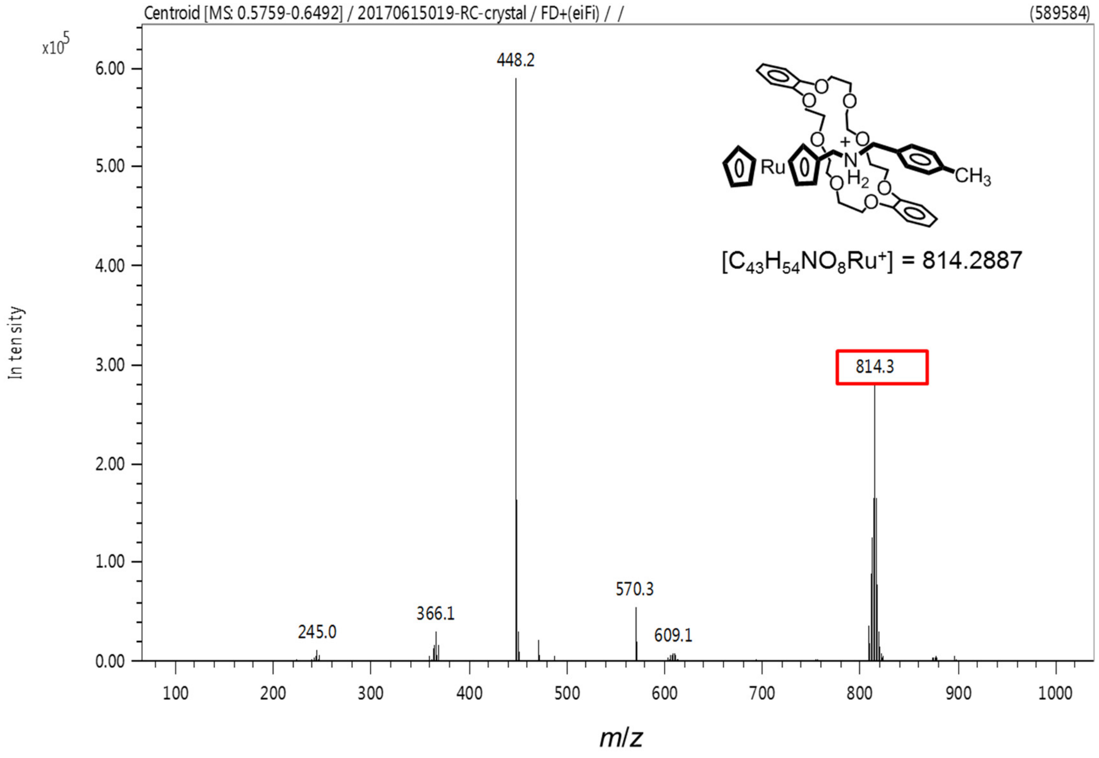

Supplementary Figure S6. LRFD mass spectra of $[Rc{\cdot}DB24C8]^{+}(PF_6)^{-}$. The main peak at *m*/*z* = 814.3 corresponds to $[Rc{\cdot}DB24C8]^{+}(PF_6)^{-}$. ($[C_{43}H_{54}NO_8Ru^{+}]$ =814.2887, error 14 ppm).

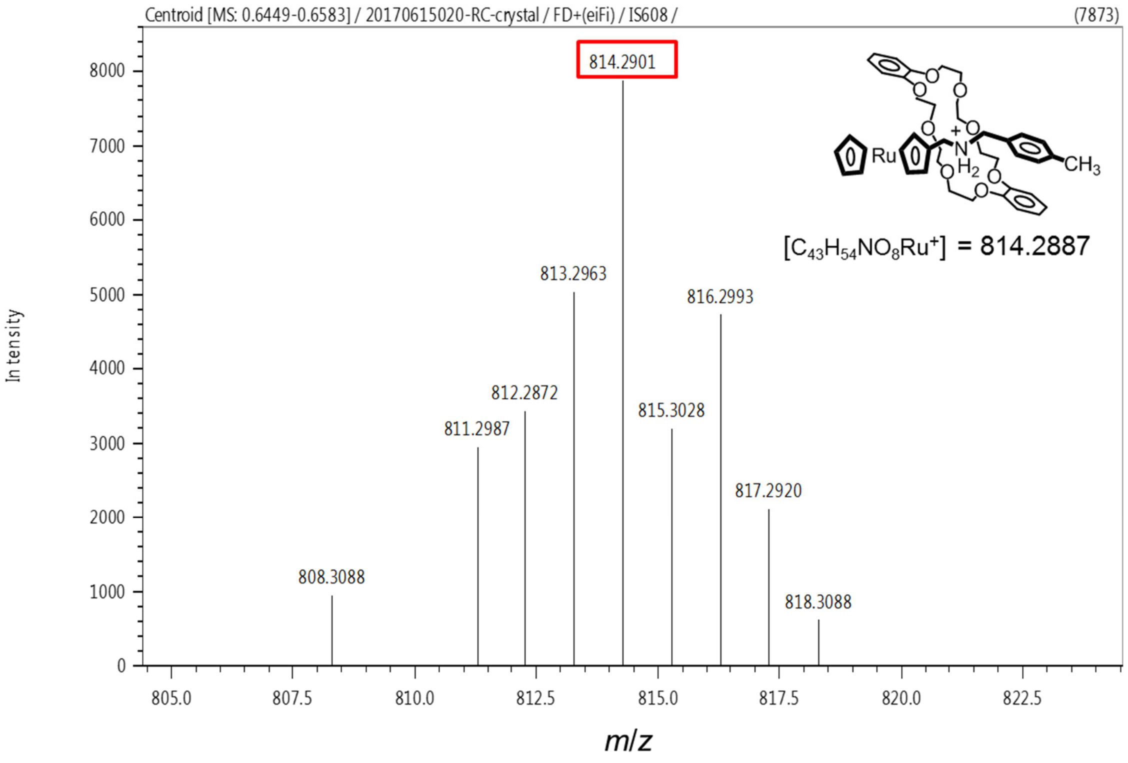

Supplementary Figure S7. HRFD mass spectra of $[Rc{\cdot}DB24C8]^{+}(PF_6)^{-}$. The main peak at *m*/*z* = 814.2901 corresponds to $[Rc{\cdot}DB24C8]^{+}(PF_6)^{-}$. ($[C_{43}H_{54}NO_8Ru^{+}]$ =814.2887, error 1.7 ppm).

The isolated crystals of [ruthenocenylmethyl(4-methylphenyl)ammonium·DB24C8]$^{+}$(PF_6)$^{-}$ were redissolved in CD_3CN. ^{1}H NMR spectra of DB24C8, [ruthenocenylmethyl(4-methylphenyl)ammonium·DB24C8]$^{+}$(PF_6)$^{-}$ and [ruthenocenylmethyl(4-methylphenyl) ammonium]$^{+}$(PF_6)$^{-}$ in CD_3CN are shown in Supplementary Figure S8. In the pseudorotaxane complex, the signals at 4.19 and 4.58 ppm are assigned to -NCH_2- in the complex. The signals associated with the aromatic protons H^{f} and H^{g} of [ruthenocenylmethyl(4-methylphenyl)ammonium]$^{+}$(PF_6)$^{-}$ shift from 7.32 and 7.26 ppm to 7.17 and 7.15 ppm upon complexation. Peaks of protons of ruthenocenyl group (H^{a}, H^{b}, H^{c}) and peaks around ammonium cation (H^{d}, H^{e}) in the axle molecule also show upfield shift. The molar ratio of complex/uncomplex for ruthenocene-containing pseudorotaxane in CD_3CN solution is estimated to be 0.72, which is 1.7 times lower than ferrocene-containing pseudorotaxane (1.25). This comparison indicates that replacement of the metal centre from Fe to Ru reduces the association between ring and ammonium axle molecules.

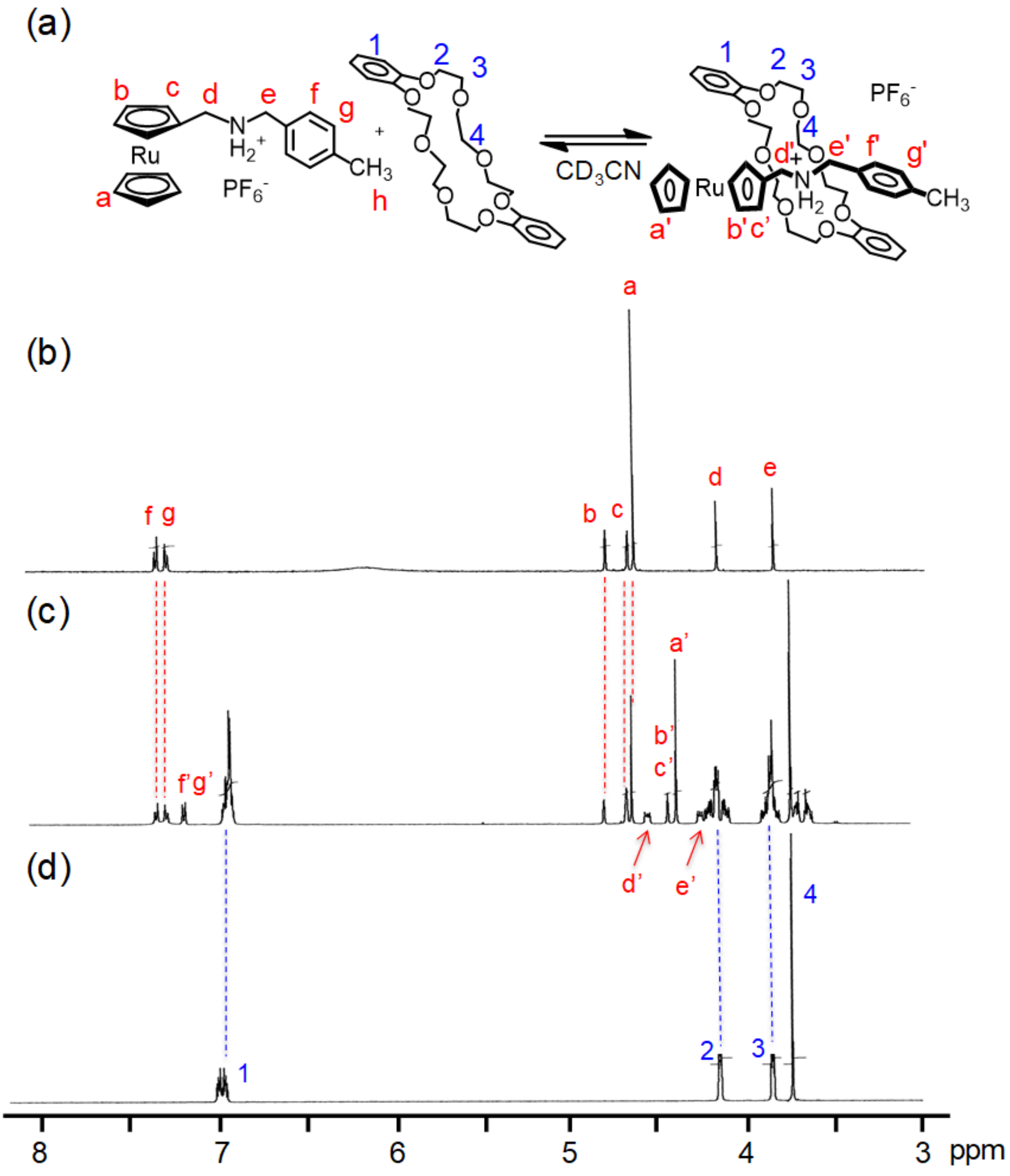

Supplementary Figure S8. ^{1}H NMR spectra (500 MHz, r.t, CD_3CN) of ruthenocene-containing pseudorotaxane and its components. (a) Dissociation/association of the pseudorotaxane in a solution. (b) ^{1}H NMR spectrum of [ruthenocenylmethyl(4-methylphenyl)-ammonium]$^+$(PF_6)$^-$, (c) [ruthenocenylmethyl(4-methylphenyl)ammonium·DB24C8]$^+$(PF_6)$^-$, (d) DB24C8.

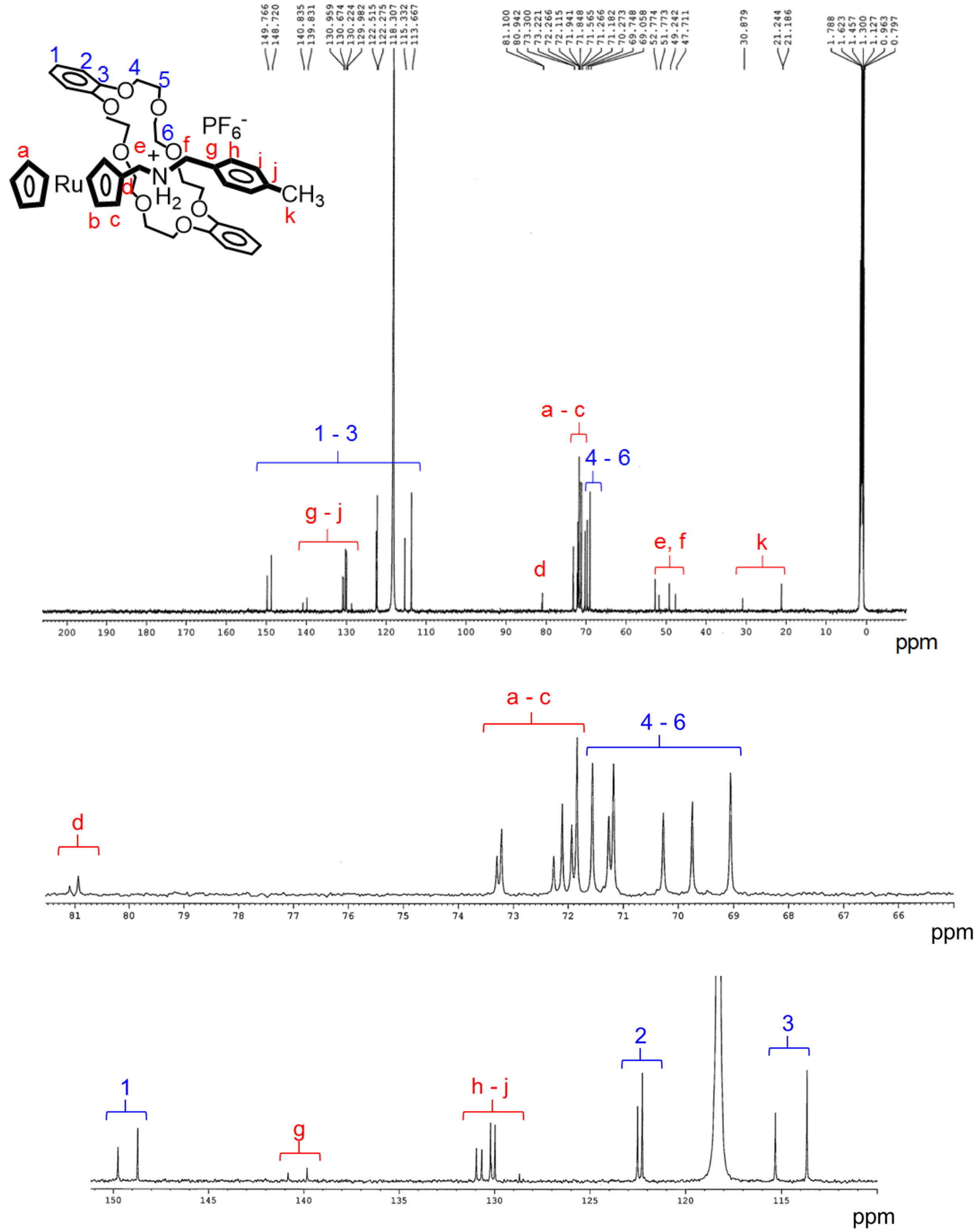

Supplementary Figure S9. ^{13}C NMR spectra (500 MHz, CD_3CN) of [ruthenocenylmethyl(4-methylphenyl) ammonium·DB24C8]$^+$(PF_6)$^-$. Signals involve the pseudorotaxane and uncomplex components in CD_3CN solution.

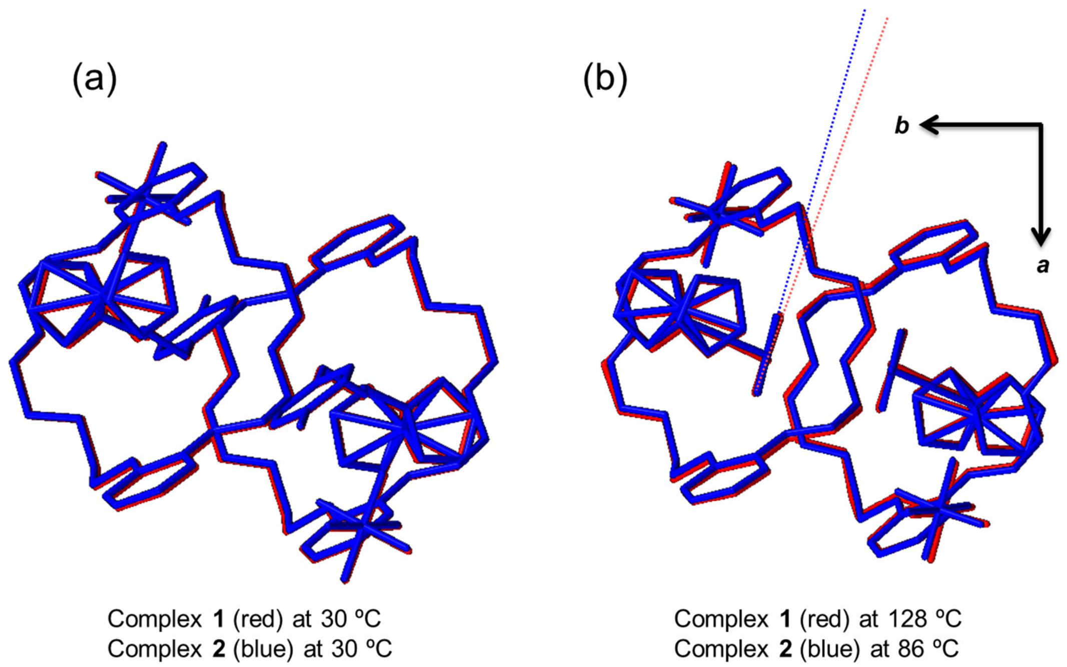

Supplementary Figure S10. Overlay drawings of molecular structures. A pair of packing structure of complex **1** and **2** obtained from single crystal X-ray crystallography (a) at 30 ºC and (b), at high temperature phase.

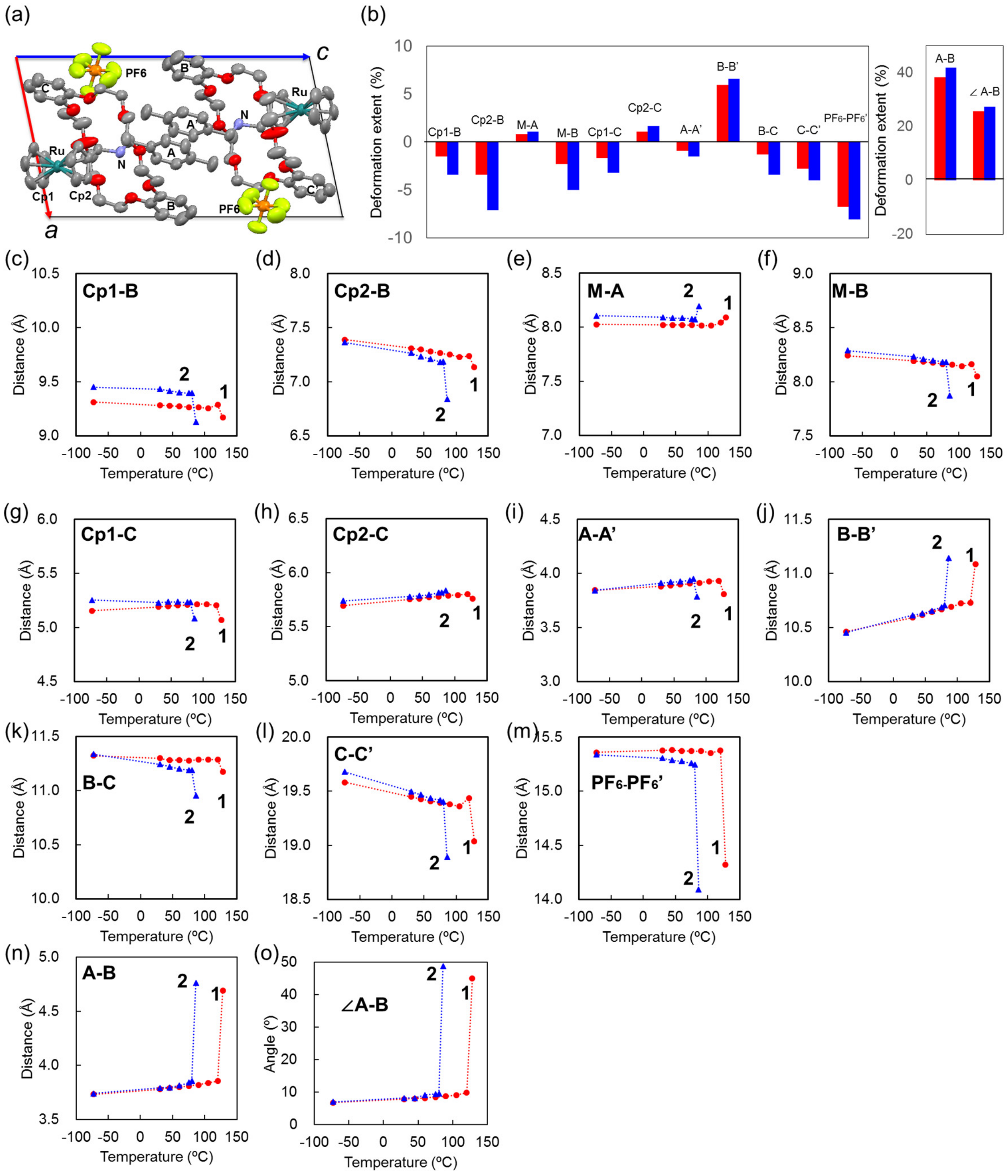

Supplementary Figure S11. Single-crystal X-ray crystallographic results of complexes **1** and **2** at various temperatures. (a) ORTEP representation of a pair of complex **2** with 30 % probability. (b) bar chart for deformation extent. (c)-(o) specific distances and angles of complex **1** (red) and **2** (blue).

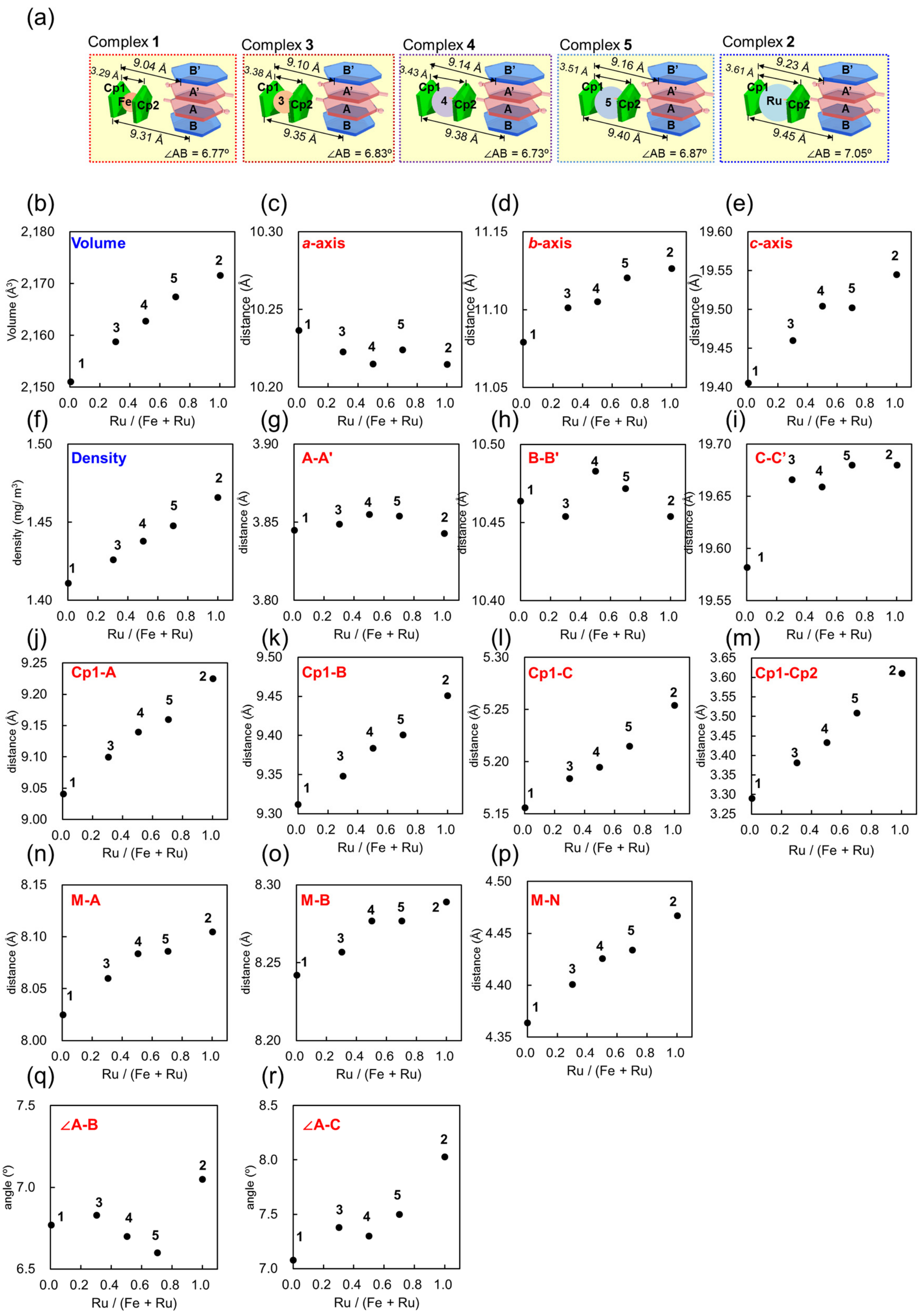

Supplementary Figure S12. Single-crystal X-ray crystallographic results of low temperature structures of complexes **1-5** at -73 ºC. (a) Illustration of mixed crystals with specific distances and angles between aromatic rings. (b)-(r) The unit cell volume, lattice parameters and intra/intermolecular distances and angles.

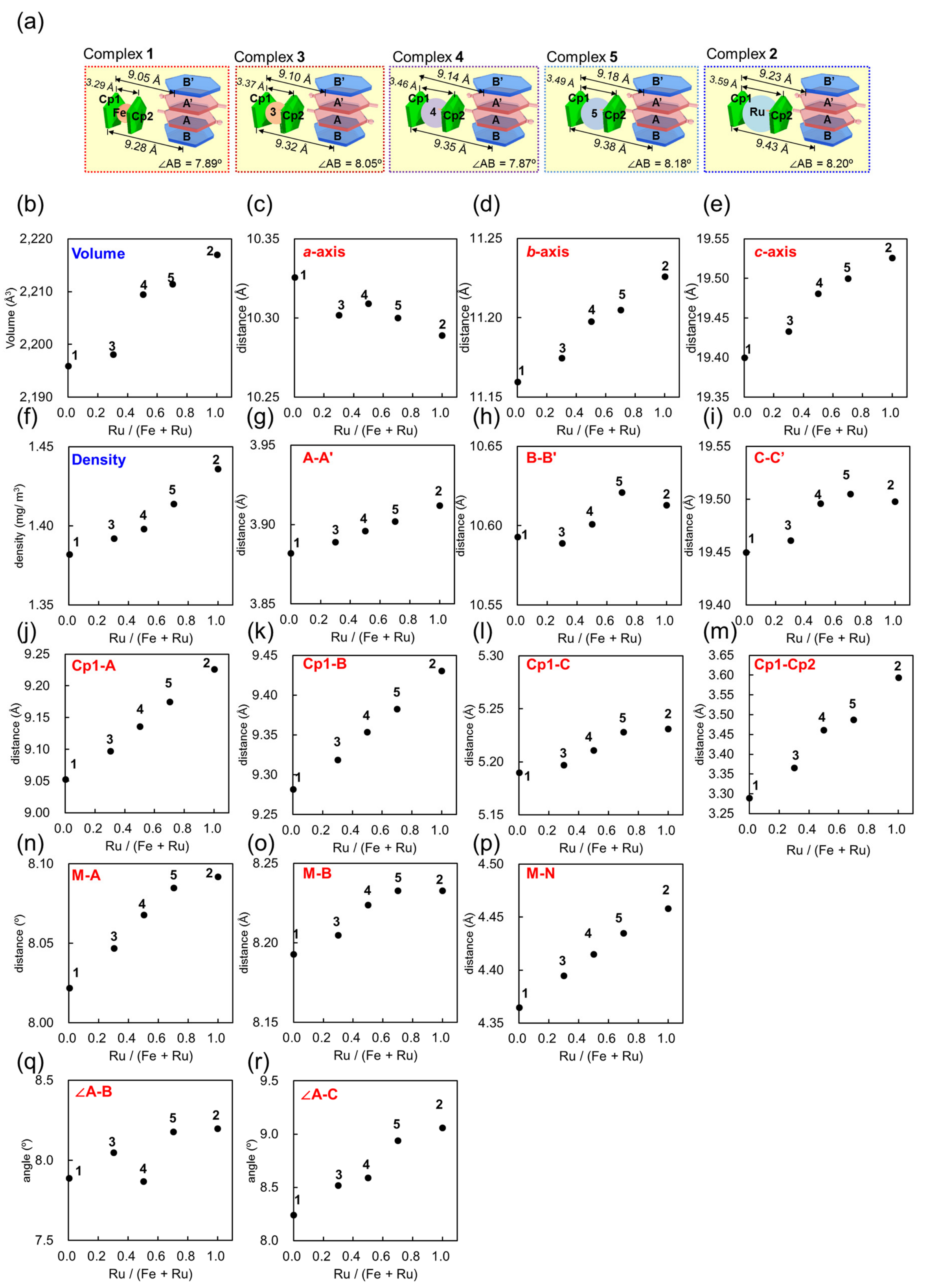

Supplementary Figure S13. Single-crystal X-ray crystallographic results of low temperature structures of complexes **1-5** at 30 ºC. (a) Illustration of mixed crystals with specific distances and angles between aromatic rings. (b)-(r) The unit cell volume, lattice parameters and intra/intermolecular distances and angles.

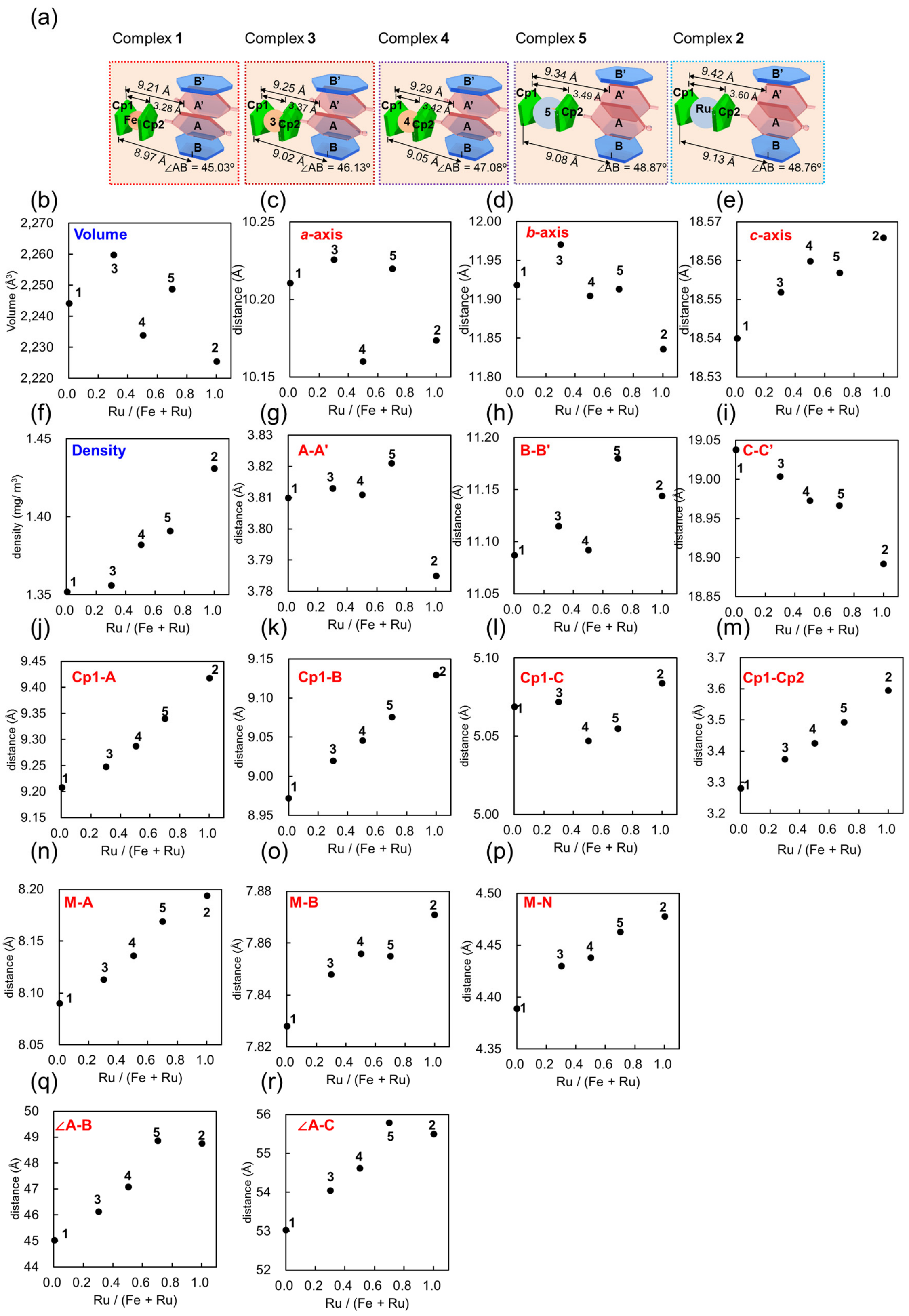

Supplementary Figure S14. Single-crystal X-ray crystallographic results of high temperature structures of complexes **1-5**. (a) Illustration of mixed crystals with specific distances and angles between aromatic rings. (b)-(r) The unit cell volume, lattice parameters and intra/intermolecular distances and angles.

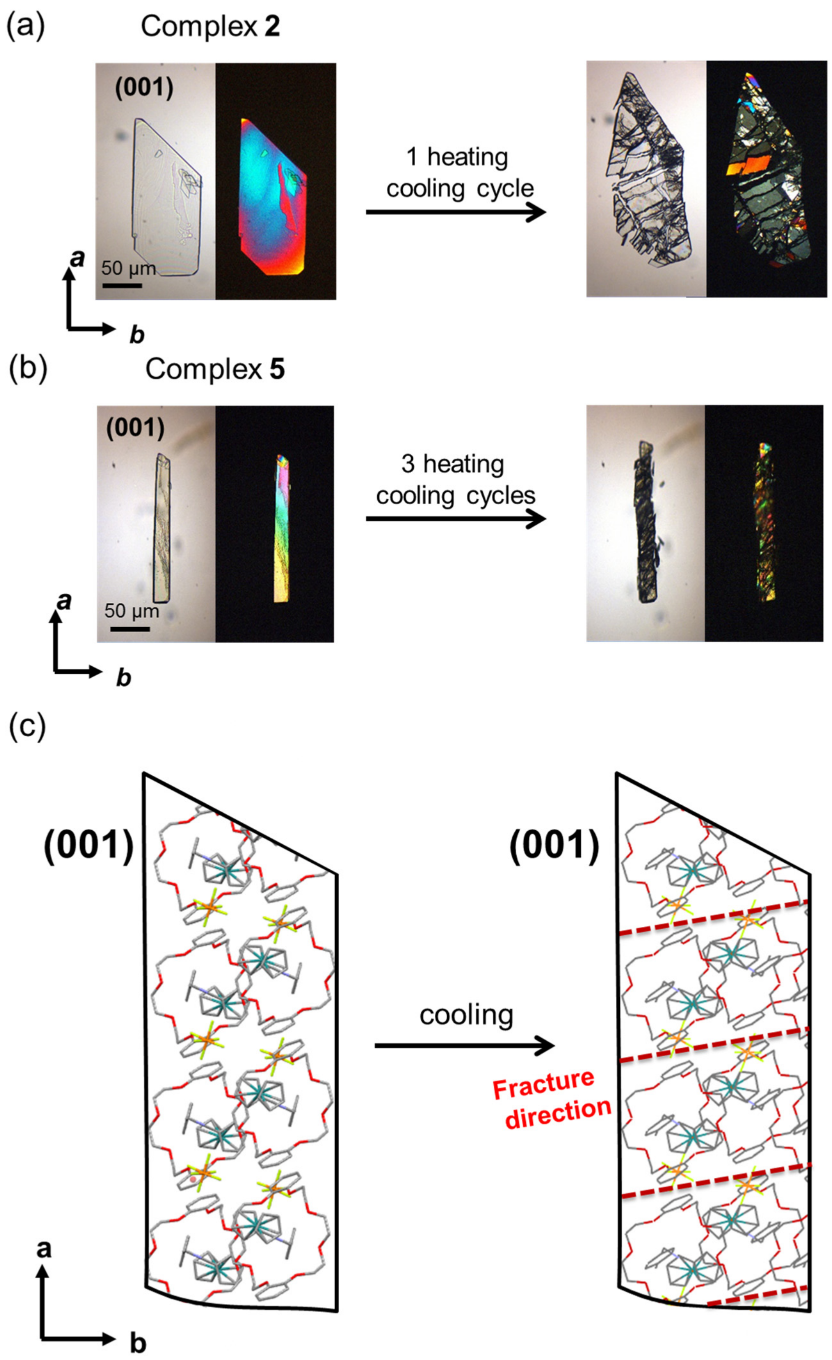

Supplementary Figure S15. Degradation of crystals of complexes **2** and **5**. (a) Optical micrographs of a single crystal of complex **2** before and after phase transition. See also the Supplementary Movie. (b) Optical micrographs of a single crystal of complex **5** before and after phase transition. (c) Packing structures of high and low temperature structures of complex **2**. Fracture direction is illustrated.

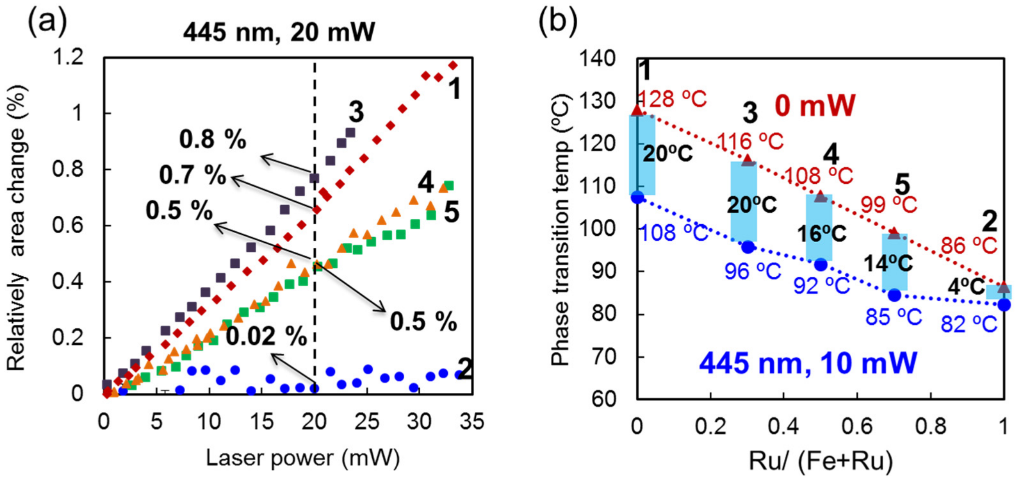

Supplementary Figure S16. Photo-responsive deformation. (a) Laser power dependence of the change in the relative area of the (001) facet of the single crystal induced by focused 445 nm laser irradiation. (b) The reduction of phase transition temperature induced by 445 nm laser irradiation.

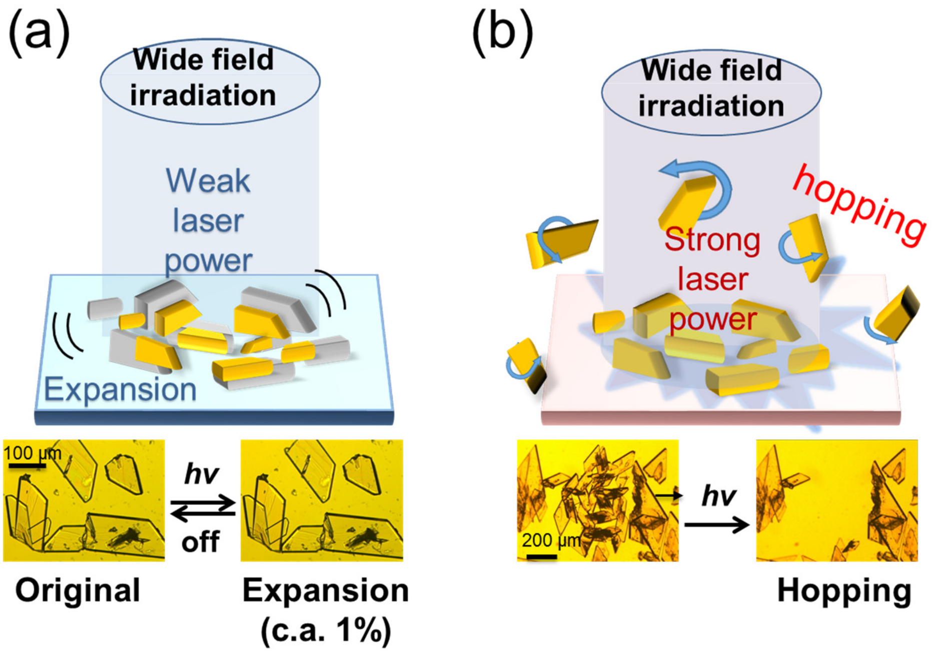

Supplementary Figure S17. Photo-triggered salient effects of complex **1**. (a) Reversible expansion and contraction of crystals of complex **1** are controlled by turning wide field laser irradiation (445 nm, 60 mW) on and off at 30 ºC. (b) Hopping of crystals of complex **1** is triggered by wide field irradiation (445 nm, 70 mW) at 30 ºC. See also the Supplementary Movie.

.

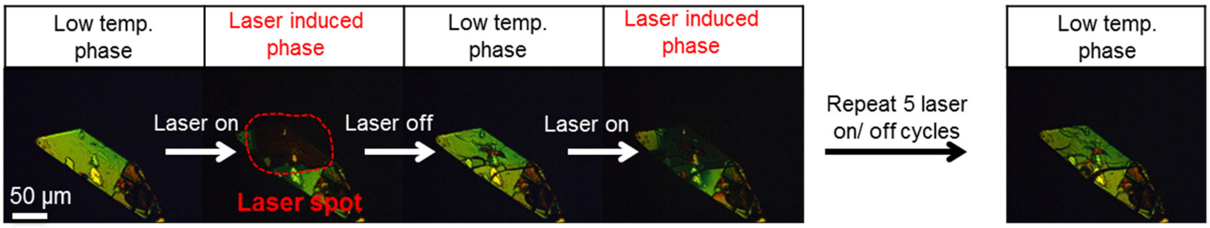

Supplementary Figure S18. Photo-triggered crystal-to-crystal phase transition of complex **3.** Thermal phase transition is controlled by focused laser (405 nm, 25 mW) at 30 ºC.

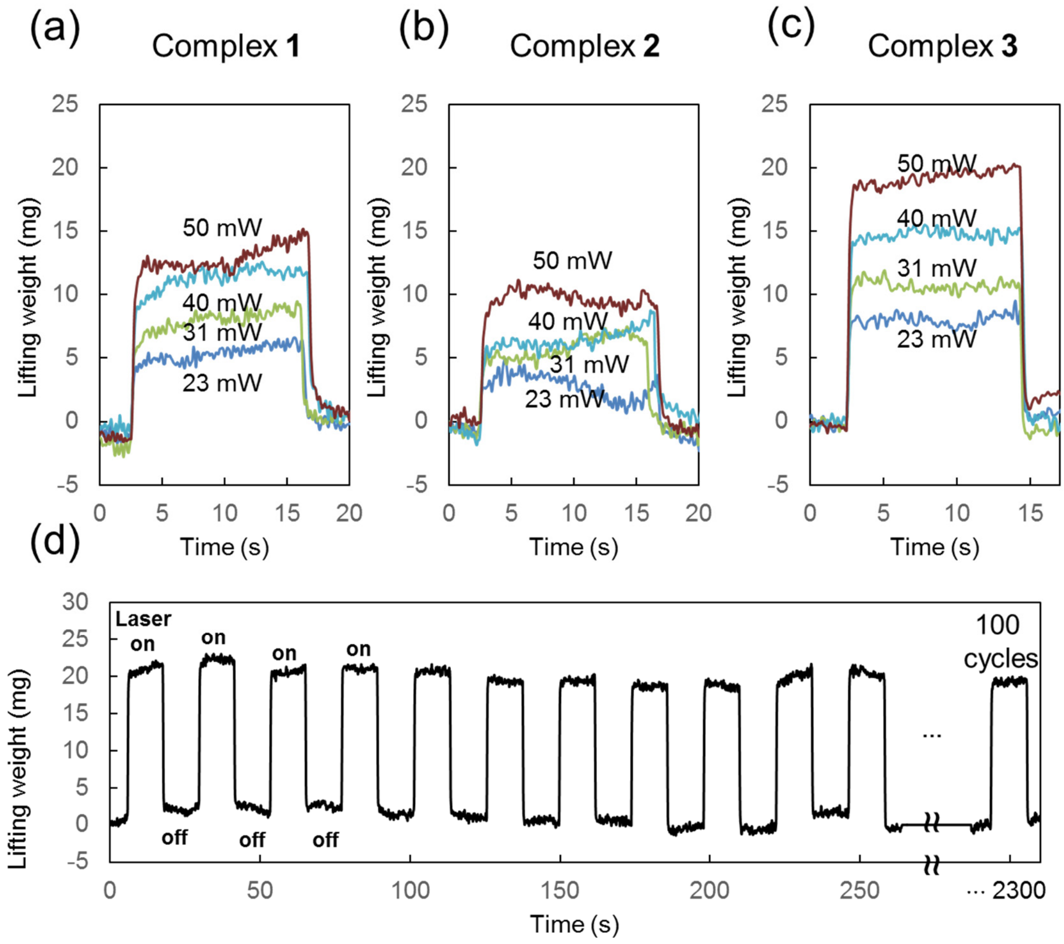

Supplementary Figure S19. Time dependence of force detection. Size expansion and contraction of the pseudorotaxane crystals are controlled by turning the 405-nm laser (50 mW) on and off at 30 ºC. (a) Time dependence of force detection for a crystal of complex **1** with a maximum lifting weight of 15 mg**.** (b) Complex **2** with a maximum lifting weight of 10 mg. (c) Complex **3** with a maximum lifting weight of 20 mg. These measurements were carried out under the same condition. (d) Repeating experiment of force detection for a crystal of complex **3**.

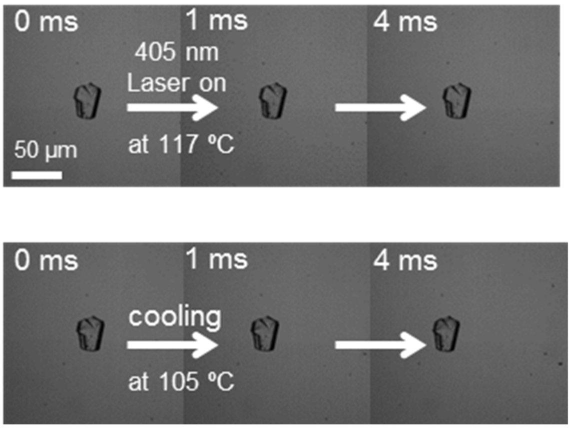

Supplementary Figure S20. Optical micrographs of a single crystal of complex **3** upon phase transition. These images were observed using a high-speed camera (1000 fps). See also the Supplementary Movie.

Supplementary Table S1. Crystal data and details of structure refinement of complex **2** at various temperatures.

Formula: $C_{43}H_{54}F_6RuNO_8P$

Molecular weight: 958.91

Crystal system: Triclinic

Space group: P1bar (No.2)

**Temp.**	**-73 °C**	**30 °C**	**45 °C**	**60 °C**	**75 °C**	**80 °C**	**84 °C**
a/ Å	10.2148(9)	10.2892(14)	10.2906(7)	10.2997(11)	10.3090(15)	10.311(2)	10.1738(18)
b/ Å	11.1265(8)	11.2261(15)	11.2476(8)	11.2779(12)	11.3223(17)	11.346(3)	11.836(2)
c/ Å	19.5452(12)	19.526(3)	19.5049(13)	19.492(2)	19.469(3)	19.453(5)	18.566(3)
α/deg	87.620(4)	87.659(3)	87.6689(15)	87.678(2)	87.658(3)	87.655(5)	86.473(3)
β/deg	78.083(4)	79.694(3)	80.0677(14)	80.431(2)	80.869(3)	81.034(5)	86.063(3)
γ/deg	89.044(5)	88.922(3)	88.8640(14)	88.815(2)	88.754(3)	88.711(5)	88.370(3)
V/ $Å^3$	2171.6(3)	2217.0(5)	2221.7(3)	2230.6(4)	2241.5(6)	2245.7(9)	2225.5(7)
Z	2	2	2	2	2	2	2
μ(MoKα) /cm^{-1}	4.76	4.66	4.65	4.63	4.61	4.60	4.64
F(000)	992	992	992	992	992	992	992
D/g cm^{-3}	1.436	1.436	1.433	1.428	1.421	1.418	1.431
crystal size /mm	0.59 x 0.10 x 0.03	0.25 x 0.20 x 0.10	0.25 x 0.20 x 0.10	0.25 x 0.20 x 0.10	0.25 x 0.20 x 0.10	0.25 x 0.20 x 0.10	0.25 x 0.20 x 0.10
Unique reflections	18976	36555	36737	36874	36761	36479	33649
Used reflections [I>2.0σ (I)]	7574	9173	9208	9255	9281	9298	9127
R	0.0505	0.0581	0.0597	0.0607	0.0622	0.0620	0.0660
*R*w	0.1117	0.1464	0.1476	0.1496	0.1484	0.1613	0.1591
GOF	1.082	1.003	0.983	0.985	0.977	0.931	0.957

Supplementary Table S2. Crystal data of low temperature structures of complexes **1** to **5** at -73 ºC.

**Temperature: -73 ºC**					
	Complex **1**	Complex **3**	Complex **4**	Complex **5**	Complex **2**
Formula in synthesis	$C_{43}H_{54}F_6Fe$ NO_8P	$C_{43}H_{54}F_6$ $Ru_{0.3}Fe_{0.7}$ NO_8P	$C_{43}H_{54}F_6$ $Ru_{0.5}Fe_{0.5}$ NO_8P	$C_{43}H_{54}F_6$ $Ru_{0.7}Fe_{0.3}$ NO_8P	$C_{43}H_{54}F_6Ru$ NO_8P
Calcd. formula from X-ray crystallography	$C_{43}H_{54}F_6Fe$ NO_8P	$C_{43}H_{54}F_6$ $Ru_{0.20}Fe_{0.80}$ NO_8P	$C_{43}H_{54}F_6$ $Ru_{0.35}Fe_{0.65}$ NO_8P	$C_{43}H_{54}F_6$ $Ru_{0.58}Fe_{042}$ NO_8P	$C_{43}H_{54}F_6Ru$ NO_8P
molecular weight	913.69	927.26	936.3	945.35	958.91
crystal system	Triclinic	Triclinic	Triclinic	Triclinic	Triclinic
space group	P1bar (No.2)	P1bar (No.2)	P1bar (No.2)	P1bar (No.2)	P1bar (No.2)
a/ Å	10.2368(6)	10.2230(4)	10.2150(7)	10.2242(5)	10.2148(9)
b/ Å	11.0792(6)	11.1013(6)	11.1053(7)	11.1205(5)	11.1265(8)
c/ Å	19.4057(10)	19.4604(10)	19.5046(13)	19.5026(11)	19.5452(12)
α/deg	87.149(13)	87.237(3)	87.272(2)	87.4130(10)	87.620(4)
β/deg	78.123(12)	78.141(2)	78.132(2)	78.1010(10)	78.083(4)
γ/deg	89.039(12)	89.001(2)	89.057(2)	89.0010(10)	89.044(5)
V/ Å^3	2151.1	2158.82(18)	2162.8(2)	2167.48(19)	2171.6(3)
Z	2	2	2	2	2
μ (MoKα) /cm^{-1}	4.66	4.69	4.71	4.74	4.76
F(000)	956	956	974	981	992
D/g cm^{-3}	1.411	1.426	1.438	1.448	1.466
crystal size /mm	0.55 x 0.39 x 0.02	0.69 x 0.19 x 0.19	0.74 x 0.55 x 0.50	0.95 x 0.91 x 0.38	0.59 x 0.10 x 0.03
Unique reflections	73602	17749	19128	19703	18976
Used reflections [I>2.0σ (I)]	7588	7557	7602	7633	7574
R	0.0383	0.0518	0.0424	0.0451	0.0505
Rw	0.0979	0.1329	0.1243	0.1311	0.1117
GOF	1.036	1.102	1.202	1.140	1.082

Supplementary Table S3. Crystal data of low temperature structures of complexes **1** to **5** at 30 ºC.

**Temperature: 30 ºC**					
	Complex **1**	Complex **3**	Complex **4**	Complex **5**	Complex **2**
Formula in synthesis	$C_{43}H_{54}F_6Fe$ NO_8P	$C_{43}H_{54}F_6$ $Ru_{0.3}Fe_{0.7}$ NO_8P	$C_{43}H_{54}F_6$ $Ru_{0.5}Fe_{0.5}$ NO_8P	$C_{43}H_{54}F_6$ $Ru_{0.7}Fe_{0.3}$ NO_8P	$C_{43}H_{54}F_6Ru$ NO_8P
Calcd. formula from X-ray crystallography	$C_{43}H_{54}F_6Fe$ NO_8P	$C_{43}H_{54}F_6$ $Ru_{0.22}Fe_{0.78}$ NO_8P	$C_{43}H_{54}F_6$ $Ru_{0.36}Fe_{0.64}$ NO_8P	$C_{43}H_{54}F_6$ $Ru_{0.62}Fe_{038}$ NO_8P	$C_{43}H_{54}F_6Ru$ NO_8P
molecular weight	913.69	921.62	930.19	941.73	958.91
crystal system	Triclinic	Triclinic	Triclinic	Triclinic	Triclinic
space group	P1bar (No.2)	P1bar (No.2)	P1bar (No.2)	P1bar (No.2)	P1bar (No.2)
a/ Å	10.3258(4)	10.3020(5)	10.3092(4)	10.300(3)	10.2892(14)
b/ Å	11.1596(4)	11.1747(6)	11.1978(5)	11.205(3)	11.2261(15)
c/ Å	19.3998(8)	19.4330(10)	19.4810(8)	19.500(4)	19.526(3)
α/deg	87.2796(10)	87.3910(10)	87.4720(10)	87.514(5)	87.659(3)
β/deg	79.5728(9)	79.6190(10)	79.5820(10)	79.592(5)	79.694(3)
γ/deg	88.8537(10)	88.8740(10)	88.8430(10)	88.968(5)	88.922(3)
V/ Å^3	2195.93	2198.1(2)	2209.50(16)	2211.4(9)	2217.0(5)
Z	2	2	2	2	2
μ (MoKα) /cm^{-1}	4.57	4.59	4.59	4.62	4.66
F(000)	956	960	969	978	992
D/g cm^{-3}	1.382	1.392	1.398	1.414	1.436
crystal size /mm	0.30 x 0.22 x 0.10	0.23 x 0.20 x 0.15	0.24 x 0.22 x 0.12	0.23 x 0.20 x 0.18	0.25 x 0.20 x 0.10
Unique reflections	33063	34156	34713	29231	36555
Used reflections [I>2.0σ (I)]	9057	9067	9089	10030	9173
R	0.0517	0.0514	0.0476	0.0546	0.0581
*R*w	0.1334	0.1357	0.1289	0.1396	0.1464
GOF	0.939	1.029	1.026	0.996	1.003

Supplementary Table S4. Crystal data of high temperature structures of complexes **1** to **5**.

	Complex **1**	Complex **3**	Complex **4**	Complex **5**	Complex **2**
**Temp.**	**128 ºC**	**120 ºC**	**111 ºC**	**102 ºC**	**84 ºC**
Formula in synthesis	$C_{43}H_{54}F_6$ $FeNO_8P$	$C_{43}H_{54}F_6$ $Ru_{0.3}Fe_{0.7}$ NO_8P	$C_{43}H_{54}F_6$ $Ru_{0.5}Fe_{0.5}$ NO_8P	$C_{43}H_{54}F_6$ $Ru_{0.7}Fe_{0.3}$ NO_8P	$C_{43}H_{54}F_6$ $RuNO_8P$
Calcd. formula from X-ray crystallography	$C_{43}H_{54}F_6$ $FeNO_8P$	$C_{43}H_{54}F_6$ $Ru_{0.26}Fe_{0.73}$ NO_8P	$C_{43}H_{54}F_6$ $Ru_{0.4}Fe_{0.6}$ NO_8P	$C_{43}H_{54}F_6$ $Ru_{0.62}Fe_{038}$ NO_8P	$C_{43}H_{54}F_6$ $RuNO_8P$
molecular weight	913.69	922.87	929.76	941.73	958.91
crystal system	Triclinic	Triclinic	Triclinic	Triclinic	Triclinic
space group	P1bar (No.2)	P1bar (No.2)	P1bar (No.2)	P1bar (No.2)	P1bar (No.2)
a/ Å	10.211 (4)	10.226(9)	10.160(12)	10.220(5)	10.1738(18)
b/ Å	11.919(6)	11.971(11)	11.905(13)	11.914(6)	11.836(2)
c/ Å	18.540(7)	18.552(17)	18.560(2)	18.557(9)	18.566(3)
α/deg	85.670(3)	86.056(18)	86.180(2)	86.118(10)	86.473(3)
β/deg	86.500(3)	86.370(18)	86.110(3)	86.150(11)	86.063(3)
γ/deg	87.660(4)	87.864(19)	88.270(3)	88.582(12)	88.370(3)
V/ Å^3	2244.30	2260.0(4)	2234.0(4)	2248.9(19)	2225.5(7)
Z	2	2	2	2	2
μ (MoKα) /cm^{-1}	4.69	4.44	4.54	4.54	4.64
F(000)	956	961	966	978	992
D/g cm^{-3}	1.352	1.356	1.382	1.391	1.431
crystal size /mm	0.20 x 0.20 x 0.08	0.25 x 0.20 x 0.16	0.30 x 0.25 x 0.13	0.24 x 0.20 x 0.17	0.25 x 0.20 x 0.10
Unique reflections	11708	28590	28193	27291	33649
Used reflections [I>2.0σ (I)]	6842	9077	8932	8815	9127
R	0.1097	0.0767	0.0683	0.0637	0.0660
Rw	0.2798	0.1939	0.1802	0.1705	0.1591
GOF	1.042	0.966	0.966	1.015	0.957

Supplementary Table S5. Intra-/intermolecular distances and angles of low temperature structures of complexes **1** to **5** at -73 ºC.

	Complex **1**	Complex **3**	Complex **4**	Complex **5**	Complex **2**
Fe:Ru	10:0	7:3	5:5	3:7	0:10
**Distance (Å)**					
Ring A-Ring B	3.733	3.730	3.733	3.741	3.740
Ring A-Ring A'	3.845	3.849	3.855	3.854	3.843
Ring B-Ring B'	10.464	10.454	10.483	10.472	10.454
Ring A-Ring C	9.274	9.300	9.303	9.300	9.310
Ring B-Ring C	11.324	11.331	11.332	11.334	11.337
Ring C-Ring C'	19.582	19.666	19.659	19.680	19.680
PF_6-PF_6'	15.361	15.352	15.352	15.343	15.338
N-Ring A	3.726	3.729	3.730	3.727	3.715
N-Ring B	5.071	5.063	5.066	5.069	5.056
N-Ring C	6.284	6.302	6.300	6.301	6.319
N-M	4.364	4.401	4.426	4.434	4.467
Cp1- Ring A	9.041	9.100	9.140	9.160	9.225
Cp1- Ring B	9.312	9.348	9.384	9.401	9.451
Cp1- Ring C	5.156	5.184	5.195	5.215	5.254
Cp2- Ring A	7.207	7.217	7.223	7.209	7.211
Cp2- Ring B	7.389	7.384	7.388	7.375	7.363
Cp2- Ring C	5.695	5.707	5.704	5.714	5.739
Cp1-Cp2	3.290	3.382	3.433	3.509	3.611
M-Ring A	8.025	8.060	8.084	8.086	8.105
M-Ring B	8.242	8.257	8.277	8.277	8.289
M-Ring C	5.213	5.219	5.217	5.221	5.222
**Angle (º)**					
A & (001)planes	71.59	71.71	71.68	71.62	71.86
B & (001) planes	65.12	65.14	65.31	65.35	65.23
C& (001) planes	66.72	66.72	66.94	66.80	66.63
A & C planes	7.08	7.38	7.30	7.50	8.03
A & B planes	6.77	6.83	6.70	6.60	7.05
B & C planes	3.43	3.83	3.73	3.87	3.87

Supplementary Table S6. Intra-/intermolecular distances and angles of low temperature structures of complexes **1** to **5** at 30 ºC.

	Complex **1**	Complex **3**	Complex **4**	Complex **5**	Complex **2**
Fe:Ru	10:0	7:3	5:5	3:7	0:10
**Distance (Å)**					
Ring A-Ring B	3.780	3.779	3.786	3.793	3.791
Ring A-Ring A’	3.882	3.889	3.896	3.902	3.912
Ring B-Ring B’	10.593	10.589	10.601	10.621	10.613
Ring A-Ring C	9.230	9.196	9.209	9.211	9.193
Ring B-Ring C	11.299	11.255	11.268	11.268	11.244
Ring C-Ring C’	19.450	19.461	19.496	19.505	19.498
PF_6-PF_6’	15.379	15.348	15.356	15.344	15.306
N-Ring A	3.791	3.717	3.720	3.721	3.708
N-Ring B	5.056	5.047	5.055	5.059	5.042
N-Ring C	6.242	6.241	6.247	6.246	6.241
N-M	4.365	4.395	4.415	4.435	4.458
Cp1- Ring A	9.053	9.097	9.136	9.175	9.226
Cp1- Ring B	9.282	9.319	9.354	9.383	9.431
Cp1- Ring C	5.190	5.197	5.211	5.228	5.231
Cp2- Ring A	7.183	7.180	7.185	7.184	7.167
Cp2- Ring B	7.309	7.298	7.302	7.294	7.265
Cp2- Ring C	5.753	5.751	5.762	5.771	5.779
Cp1-Cp2	3.286	3.366	3.461	3.487	3.594
M-Ring A	8.022	8.047	8.068	8.085	8.092
M-Ring B	8.193	8.205	8.224	8.233	8.233
M-Ring C	5.258	5.256	5.260	5.262	5.251
**Angle (º)**					
A & (001)planes	72.73	72.85	72.96	73.10	73.26
B & (001) planes	65.34	65.37	65.61	65.57	65.74
C& (001) planes	67.06	67.15	67.07	67.18	67.07
A & C planes	8.24	8.52	8.59	8.94	9.06
A & B planes	7.89	8.05	7.87	8.18	8.20
B & C planes	3.60	3.70	3.64	3.77	3.52

Supplementary Table S7. Intra-/intermolecular distances and angles of high temperature structures of complexes **1** to **5**.

	Complex **1**	Complex **3**	Complex **4**	Complex **5**	Complex **2**
Fe:Ru	10:0	7:3	5:5	3:7	0:10
**Distance (Å)**					
Ring A-Ring B	4.692	4.702	4.705	4.765	4.761
Ring A-Ring A'	3.810	3.813	3.811	3.821	3.785
Ring B-Ring B'	11.087	11.115	11.092	11.180	11.144
Ring A-Ring C	8.775	8.775	8.782	8.780	8.785
Ring B-Ring C	10.961	10.971	10.955	10.964	10.954
Ring C-Ring C'	19.038	19.004	18.973	18.967	18.892
PF_6-PF_6'	14.324	14.287	14.214	14.188	14.092
N-Ring A	3.713	3.695	3.706	3.713	3.723
N-Ring B	5.061	5.054	5.029	5.023	5.007
N-Ring C	6.002	6.022	6.030	6.050	6.054
N-M	4.389	4.430	4.438	4.463	4.478
Cp1- Ring A	9.208	9.248	9.288	9.340	9.418
Cp1- Ring B	8.972	9.020	9.046	9.076	9.130
Cp1- Ring C	5.069	5.072	5.047	5.055	5.084
Cp2- Ring A	7.141	7.146	7.138	7.151	7.151
Cp2- Ring B	6.889	6.887	6.862	6.842	6.837
Cp2- Ring C	5.757	5.803	5.796	5.834	5.834
Cp1-Cp2	3.282	3.374	3.426	3.493	3.595
M-Ring A	8.090	8.113	8.136	8.169	8.194
M-Ring B	7.828	7.848	7.856	7.855	7.871
M-Ring C	5.198	5.216	5.191	5.218	5.204
**Angle (°)**					
A & (001)planes	87.81	87.61	87.80	87.24	87.35
B & (001) planes	68.69	69.59	69.83	69.98	70.09
C& (001) planes	70.60	70.48	70.44	70.07	69.75
A & C planes	53.04	54.05	54.62	55.79	55.50
A & B planes	45.03	46.13	47.08	48.87	48.76
B & C planes	9.65	8.90	8.30	7.42	7.07

Supplementary Table S8. Thermodynamic parameters of crystals accompanied by phase transition estimated from DSC.

Complex	Fe:Ru	Endothermic peaks				Exothermic peaks			
		Temp. (°C)	ΔH (kJ mol^{-1})	ΔS (J K^{-1} mol^{-1})	***x**	Temp. (°C)	ΔH (kJ mol^{-1})	ΔS (J K^{-1} mol^{-1})	***x**
**1**	10:0	128	7.7	19	9.8	115	8.1	20	11.1
**3**	7:3	118	6.1	16	6.9	106	6.9	18	8.7
**4**	5:5	108	5.9	16	6.9	92	6.5	17	7.7
**5**	3:7	99	6.3	17	7.7	78	6.7	18	8.7
**2**	0:10	86	6.1	17	7.7	55	5.8	16	6.9

*The number of energetically equivalent microscopic states x were estimated from from ΔS values using $\Delta S = R \ln(x)$. R is the gas constant.
