# Supplementary material for: Dynamic Pseudorotaxane Crystals Containing Metallocene Complexes
Source: Sci Rep. 2017 Oct 27;7:14195. doi: 10.1038/s41598-017-14505-7 (PMC5660174; doi:10.1038/s41598-017-14505-7)
Supplement: Supplementary file 1 — Supplement CIF files [file 41598_2017_14505_MOESM1_ESM.zip › 168049_1_rebuttal_5173052_qwjhhq.docx]

**Reviewer comments:**

**Reviewer #1 (Remarks to the Author):**

Comments:

The manuscript presents thermo- and photoresponsive dynamic pseudorotaxane crystals composed of axle molecules containing ferrocene or ruthenocene groups threaded through dibenzo[24]crown-8 ether rings. The crystallographic work was done well. Thermally induced phase transition and photo-induced switching of pseudorotaxane crystals were investigated. This work is somewhat interesting, and so it may be suitable to be published in Scientific Reports after minor revisions.

1. In page 5 (the last line) and 6 (lines 1-3), the complexes 3-5 actually are physical mixture, is it possibility to obtain complexes 3-5 through synthesis by controlling the ratios of raw materials and character their structures by single-crystal X-ray diffraction?

**Authors**: The complexes **3-5** obtained from this recrystallization process are not physical mixtures, but chemically mixed crystals. By dissolving complexes **1** and **2** in dichloromethane, a dynamic equilibrium between complexed and uncomplexed axle/ring molecules provides the mixed crystals via slow evaporation of ethyl ether.

We estimated the exact Fe/Ru ratios by single-crystal X-ray crystallography, as shown in formula in Tables S3-5. This technique is based on a substitutional disorder provided by occupation by different types of atoms at the same site. After refinement, the accurate ratio of Fe/Ru value was obtained from “res file”. These values were consistent with the mixing ratios in the synthesis.

2. In page 23-24, the molar ratio of the reactance is 0.45:0.58 in the synthesis of [ruthenocenylmethyl(4-methylphenyl) ammonium]^+^(PF_6_)^-^, but 1:1 in products. If the molar ratio of the reactance was changed to 1:1, how about the result?

**Authors**: In this step, according to our experience, slightly overdose of NH_4_PF_6_ increases the product yield and purity. The unreacted NH_4_PF_6_ and the byproduct NH_4_Cl can be easily washed with H_2_O. If the molar ratio was changed to 1:1, the anion conversion might not be completed.

3. Please conform some numbers. In page 23, p-xylyaminomethylruthenocene (163.6 g, 0.45 mmol)? In page 24, preparation of [ruthenocenylmethyl(4-methylphenyl) ammonium⋅DB24C8] ^+^(PF_6_)^-^ rotaxane crystal, the theory molar of the product is 0.028mmol, but the product molar is 70 mmol.

**Authors**: This is our mistake. In page 23, p-xylyaminomethylruthenocene was corrected from 163.6 g to 163.6 mg. Also, in page 24, the product molar was corrected from 70 mmol to 0.020 mmol.

4. Please check the NMR data, some of them are not presented correctly.

**Authors**: We carefully rechecked 1H NMR spectrum of the pseudorotaxane complex and found wrong assignments. In particular, peaks at 4.19 and 4.58 ppm (d’ and e’ in Fig. S8) are typical signals corresponding to -NCH2- in the complex. Corrected assignments are shown in Supplementary Fig. S8 and the experimental section in page 24.

To avoid duplication of information, previous Figs. S6 and S11 (1H NMR of the pseudorotaxane) and previous Table S1 (summary) were removed in the revised version.

We appreciate your careful check.

**Reviewer #2 (Remarks to the Author):**

The authors reported the five metallocene–containing dynamic pseudorotaxane crystalline materials that respond to thermal– and light–stimuli. The five complexes are appropriately characterized via X-ray single crystal analysis and optical microscopy. The structure – crystal deformation relationship is discussed in detail. This study is useful in designing smart crystalline materials such as actuators.

I have several concerns regarding the following issues:

1. The photo-irradiation experiments show that the transition temperatures decrease upon laser irradiation. Furthermore, photomechanical conversion is clearly observed in the compounds. These results are of interest to readers of the journal. However, the mechanism of them is very unclear. Is the mechanism purely photo-effect or photo-thermal effect? The authors should clearly address this issue in detail.

**Authors**: These phenomena are based on photo-assisted thermal effect. We reported the mechanism behind these photo-irradiation experiments in *Nat. Commum*, **2016**, 7, 13321. The effect of laser irradiation on the temperature of crystal was analyzed through a thermographic measurement. We concluded that the photoinduced crystal-to-crystal phase transition was related to increase in a crystal temperature. Interestingly, according to the single crystal X-ray crystallography, the photoinduced molecular structure is different from the thermal induced structure. The effect of the laser irradiation on the length of the *c* axis can be attributed to the photoexcitation of the ferrocenyl group, resulting in the expansion of thickness and a positive force can be detected as an evidence of the photomechanical conversion. In short, photoirradiation would not only heat the crystals, but also provide an extra expansion motion in *c*-axis direction.

In page 16 last paragraph, following paragraph was added to explain the mechanism briefly.

“The photoinduced expansion/contraction and the decrease in the phase transition temperatures can be accounted for by the photo-thermal conversion of the crystals. In our previous study, the effect of laser irradiation on the surface temperature of the pseudorotaxane crystals was analysed through thermographic measurement, which showed that the crystal temperature rapidly increased by 10-15 °C by 445nm laser irradiation^43^. This causes the photoactivation of the ferrocenyl group in the pseudorotaxanes, leading to the elongation of the crystal lattice in 3D as well as the photo-assisted thermal phase transitions.”

2. It would be useful if the authors discuss the driving force of the thermal phase transition. As a minimum requirement, the authors should estimate the number of energetically equivalent microscopic states x from ΔS values using ΔS = R ln(x).

**Authors**: The number of energetically equivalent microscopic states x were estimated from ΔS values, and these values are added in the Table S8. We appreciate your fruitful suggestions to improve our manuscript.

**Reviewer #3 (Remarks to the Author):**

In this paper, Prof. Horie reported a thermo- and photoresponsive dynamic pseudorotaxane crystals based on the host-guest complex between dibenzo[24]crown-8 ether rings and ferrocene or ruthenocene derivatives. The crystal-to-crystal thermal phase transition of these ruthenocene-, ferrocene-containing pseudorotaxanes or mixed ones were systematically studied. Single-crystal X-ray crystallography at various temperatures demonstrated the details of the structural changes of those pseudorotaxanes, which made the results very interesting. The photoresponsive dynamic properties of pseudorotaxanes were also investigated, which could provide a lifting force 6,400-times than the weight of the crystal itself upon the phase transition. This study is very interesting, and particularly, single-crystal X-ray crystallography gave the details of structures, which help to understand well the changes of dynamic interlocked molecules in the crystal state. The manuscript was also prepared well.

Therefore, publication is recommended. Some questions are shown below:

1. In Figure 1a, the "Rotaxane complexes" should be corrected into "pseudorotaxane complexes".

**Authors**: This was corrected accordingly.

2. The mixed ruthenocene- and ferrocene-crystals were studied. How was their accurate ratio in the crystal determined?

**Authors**: We estimated the exact Fe/Ru ratios by single-crystal X-ray crystallography, as shown in formula in Tables S2-4. This technique is based on a substitutional disorder provided by occupation by different types of atoms at the same site. After refinement, the accurate ratio of Fe/Ru value was obtained from “res file”. These values were consistent with the mixing ratios in the synthesis.

In page 6 first paragraph, following sentences were added.

“Fe:Ru ratios were estimated by single-crystal X-ray crystallography based on a substitutional disorder provided by occupation by different types of atoms at the same site. These values were consistent with the mixing ratios in the synthesis.”

3. In Figure 2b, the unit cell volume V of complex 1 and 2 decreased upon continuous heating after reaching the crystal phase transition. How does it happen, and what is the crystal structure over higher temperature?

**Authors**: Upon heating beyond the phase transition temperatures, discontinuous changes in the unit cell parameters were observed. The shrinkage of unit cell volume can be correlated to the decrease in *a-* and *c-* axes directions. The most significant decrease in *c*-axis is due to the twist of tolyl group.

We are also interested in the higher temperature structures. According to the polarizing optical microscopy, these crystals seem to have one or more phase transitions. However, single-crystal X-ray crystallography for these crystals at such high temperatures is very difficult because of decomposition of the crystals within about two hours. We will report if we succeed to observe further reliable structures in the future.

4. When the photoresponsive experiment was conducted, how did the author eliminate the heat effect introduced by the laser irradiation?

**Authors**: We coordinate heat with light rather than eliminating the heat effect. In order to clarify the relationship between laser light and heat, some experiments were conducted. The result was reported in *Nat. Commum*, **2016**, 7, 13321.

In page 16 last paragraph, following paragraph was added to explain the mechanism briefly.

“The photoinduced expansion/contraction and the decrease in the phase transition temperatures can be accounted for by the photo-thermal conversion of the crystals. In our previous study, the effect of laser irradiation on the surface temperature of the pseudorotaxane crystals was analysed through thermographic measurement, which showed that the crystal temperature rapidly increased by 10-15 °C by 445nm laser irradiation^43^. This causes the photoactivation of the ferrocenyl group in the pseudorotaxanes, leading to the elongation of the crystal lattice in 3D as well as the photo-assisted thermal phase transitions.”

We appreciate your comments to improve our manuscript.
